# MODIFI: protocol for randomised feasibility study of eye-movement desensitisation and reprocessing therapy (EMDR) for functional neurological disorder (FND)

**DOI:** 10.1136/bmjopen-2023-073727

**Published:** 2023-06-02

**Authors:** Sarah R Cope, Jared G Smith, Sharif El-Leithy, Serena Vanzan, Caitlin Pentland, Susannah Pick, Dawn Golder, Patricia Hogwood, Kati Turner, Jo Billings, Mark J Edwards

**Affiliations:** 1 South West London and St George’s Mental Health NHS Trust, Tooting, UK; 2 Population Health Research Institute, St George’s University of London, London, UK; 3 Clinical Research Unit, South West London and St George's Mental Health NHS Trust, London, UK; 4 Traumatic Stress Service, South West London and St George's Mental Health NHS Trust, London, UK; 5 Department of Psychological Medicine, Institute of Psychiatry Psychology and Neuroscience, London, UK; 6 FND Hope UK, London, UK; 7 Independent Researcher, London, UK; 8 Department of Psychiatry, University College London, London, UK; 9 Department of Basic and Clinical Neuroscience, Institute of Psychiatry, Psychology & Neuroscience, King's College London, London, UK

**Keywords:** neurology, adult psychiatry, clinical trials

## Abstract

**Introduction:**

Functional neurological disorder (FND) refers to an involuntary loss of control over and/or aberrant perception of the body. Common presenting symptoms are functional (non-epileptic) seizures, and functional motor disorder, for example, walking difficulties, weakness or tremor. Greater access to effective treatments would lead to reduced distress and disability; and reduce unnecessary healthcare costs.

This study will examine eye-movement desensitisation and reprocessing therapy (EMDR) as a treatment for FND. EMDR is an evidence-based treatment for post-traumatic stress disorder (PTSD), but its use for other conditions is growing. An FND-specific EMDR protocol will be tested, and if the intervention proves feasible with promising clinical outcomes, progression to a substantive study could take place.

**Methods and analysis:**

Fifty adult patients diagnosed with FND will be recruited. It will be a single-blind randomised controlled trial with two arms: EMDR (plus standard neuropsychiatric care; NPC) and standard NPC. The two groups will be compared at baseline (T0), 3 months (T1), 6 months (T2) and 9 months (T3). Measures of feasibility include safety, recruitment, retention, treatment adherence and acceptability. Clinical outcome measures will assess health-related functioning/quality of life, ratings of FND symptoms and severity, depression, anxiety, PTSD, dissociation, service utilisation and other costs. Improvement and satisfaction ratings will also be assessed. Feasibility outcomes will be summarised using descriptive statistics. Exploratory analyses using (linear/logistic) mixed-effect models will examine the rate of change in the groups’ clinical outcome measures across the four time-points.

After the intervention period, a sample of participants, and clinicians, will be invited to attend semistructured interviews. The interviews will be analysed using reflexive thematic analysis.

**Ethics and dissemination:**

This study has been approved by the NHS West Midlands—Edgbaston Research Ethics Committee. Study findings will be published in open access peer-reviewed journals, presented at conferences, and communicated to participants and other relevant stakeholders.

**Trial registration:**

NCT05455450 (www.clinicaltrials.gov).

**Strengths and limitations of this study**

- This is a pragmatic randomised controlled trial embedded within an existing clinical service.
- The eye-movement desensitisation and reprocessing therapy is tailored for functional neurological disorder presentations.
- There has been patient and public involvement (PPI) input from the design stage, and there is continued PPI involvement.
- The study will use a validated self-report measure to assess post-traumatic stress disorder, rather than a clinical interview.

## Introduction

Functional neurological disorder (FND) is a disorder at the interface between neurology and psychiatry. It refers to an involuntary loss of control over and/or aberrant perception of the body. Presenting symptoms can be wide ranging with the most common being functional (non-epileptic) seizures (FS) and functional motor disorder (mFND), for example, walking difficulties, weakness, tremor. Risk and perpetuating factors for FND include traumatic experiences, affective disorders and experiencing chronic or acute illness. FND is one of the most common diagnoses made in neurology, for example, 16% of new patients in general neurology^1^; causes similar disability and impairment in quality of life as Parkinson’s disease and multiple sclerosis, and high unemployment.^2^ Lack of provision of assessment and treatment is associated with significant unnecessary costs, for example, unnecessary referrals, investigations, and emergency department attendances.^3 4^

Accessing treatment is often difficult, and effective treatments for FND are still being established. Healthcare Improvement Scotland published guidance in 2012, recommending a stepped-care approach, whereby patients are assessed and diagnosed by a neurologist, and referred for relevant interventions (eg, physiotherapy, psychology, psychiatry, occupational therapy) as required.^5^ A cornerstone of FND treatment is effective communication of the diagnosis, and guidelines regarding management of FND have been published.^6 7^ There have been consensus recommendations published for physiotherapy for mFND and occupational therapy.^8 9^ The best evidence for mFND specifically comes from a feasibility study evaluating a specialist physiotherapy intervention compared with physiotherapy in the community, which reported positive outcomes in terms of recruitment, retention, acceptability and clinically meaningful effect sizes.^10^ A multicentre NIHR-funded randomised controlled trial (RCT) evaluating this is underway (International Standard Randomised Controlled Trials Number ISRCTN56136713). However, this intervention is not suitable for a large proportion of patients with mFND (only 32% of eligible patients met the inclusion criteria; most common reasons for exclusion were dominant persistent pain and psychological factors requiring treatment). There have been reports of beneficial outcomes following cognitive behavioural therapy (CBT) in uncontrolled studies for patients with mFND, but no controlled and adequately powered studies have been carried out.^11^ For FS specifically, the multicentre CODES trial compared CBT plus standard medical care (SMC) to SMC alone. Although this study did not find a significant difference in monthly seizure frequency between the groups, they did find significant improvements for CBT on secondary measures (psychosocial functioning, psychological distress and health-related quality of life).^12^ Previous studies evaluating CBT for FS have reported significant improvements in seizure frequency.^13 14^ More research regarding effective psychological treatments other than CBT for FND is needed, to inform delivery of treatment.

Cross-sectional studies suggest that lifetime traumatic/adverse experiences are higher in FND populations, when compared with healthy controls; in particular for those with FS, who also have higher incidences of post-traumatic stress disorder (PTSD).^15^ The occurrence of severe life events immediately prior to symptom onset is significantly more frequent in those with mFND compared with psychiatric controls^16^ and it has been proposed that there is a trauma subtype of FND.^17^ Traumatic/adverse life events, including physical events such as injury or illness, are a risk factor and can be a trigger for developing FND. Mechanistic models of FND have been developed focusing on different levels of explanation from the neurobiological to the psychosocial. Neurobiological models have used predictive coding models of perception and movement control to suggest that symptoms in FND relate to the development of abnormal priors which are activated by misdirected attention towards the body.^18^ This links closely with cognitive models suggesting that learnt patterns of behaviour are triggered by abnormal threat processing.^19^ Emotional dysregulation, abnormal interoceptive processing and alexithymia can all be integrated with such models, building a complex picture of the biological and psychological processes that underpin FND.^20^ This provides a scientific foundation for the development and application of specific psychological interventions to treat people with FND.

Eye-movement desensitisation and reprocessing therapy (EMDR) is an evidence-based treatment for PTSD, but its use for other conditions is growing, including treatment of somatic symptoms such as persistent pain and tinnitus.^21–24^ EMDR follows a standard protocol (see table 1).^25^ Within EMDR, a target memory is brought to mind, while the clinician creates a distracting task that means the person’s attention is divided between the memory and the present-focused task. Traditionally, eye-movements are used, but other tasks that create dual attention can be used, for example, tapping. The working memory (WM) hypothesis suggests that focusing on the memory, while engaging in a competing task, results in the memory becoming less vivid and distressing. A recent systematic review, identifying 11 studies testing the WM hypothesis, concluded that bringing a distressing memory to mind, while engaging in a secondary task, results in reduced vividness and emotionality of the memory, and is associated with symptom reduction.^26^

**Table 1:** The eight phases of EMDR according to the standard protocol (Shapiro 2018)

Phase	Description
I–II	Taking of patient history, assessment of suitability for EMDR and preparation for the therapy.
III	Assessment of a target image whereby the patient is asked to bring a target memory to mind, identify the most upsetting image or moment and identify the negative cognition about themselves that goes with that moment. They are also asked to identify a positive cognition and rate their belief in that cognition. Additionally, they are asked to rate their subjective distress, identify the associated emotions and locate where they feel the distress in their body.
IV	Desensitisation phase: the patient is asked to bring the target memory to mind, with the negative cognition, notice where they are feeling the distress in their body and follow the clinician’s fingers with their eyes (or other alternating task that taxes working memory). After each set of eye movements, the patient is asked what they noticed, and importantly, without discussion, they are told to ‘go with that’ alongside the eye movements. Once the distress has reduced sufficiently (this may involve multiple sessions), the clinician proceeds to the installation phase.
V	Installation phase: positive cognition is installed, aided by eye-movements (or alternative).
VI	Target any remaining distress in the body.
VII	Closure of the session.
VIII	Assess previously targeted material and whether or not further processing is required.

EMDR, eye-movement desensitisation and reprocessing therapy.

A systematic review of EMDR as a treatment for FND reported three case studies/series with all five cases presented having comorbid PTSD, of which four cases were successfully treated.^27–30^ EMDR has been reported as a useful adjunctive therapy for two FND cases without PTSD, whereby both cases achieved resolution of FND symptoms and less distress.^31^ EMDR can focus on specific past adverse experiences that are contributing to pathology, memories associated with when FND symptoms began, current FND symptoms and future predictions regarding symptoms. Theoretically, targeting distressing memories/images associated with FND could weaken cognitive representations of symptoms, and reduce threat associated with symptoms, meaning that representations of the symptoms are less easily triggered, resulting in fewer symptoms and less distress. EMDR is a therapy that can be tailored to the heterogeneous presentations of FND.

This study aims to evaluate the feasibility and acceptability of conducting a full-scale trial of EMDR for people diagnosed with FND. Feasibility will be assessed by examining recruitment rate, intervention adherence and retention. Acceptability will be examined through attendance rates, satisfaction ratings, therapy fidelity ratings and qualitative interviews with participants and treating therapists. Assessment of safety across the two arms will be compared. Examination of the completeness of outcome measures and variance in outcomes will be used to inform the design and power calculation of a future definitive trial.

### Study objectives

1. Test the acceptability and feasibility of an FND-specific EMDR intervention protocol, delivered in-person or virtually. For a substantive RCT, the intervention will be subject to amendment based on the results of this trial.
2. Investigate the value of a range of outcome measures, to determine the outcome measure with greatest effect size to enable a sample size calculation for a substantive RCT.
3. Carry out semistructured interviews with participants and therapists to explore experiences of EMDR and the trial; informing the intervention and design of a substantive trial.

## Methods and analysis

This protocol is reported in accordance with the Standard Protocol Items: Recommendations for Intervention Trials 2013 statement.^32^

### Study design

This feasibility study is a single-blind RCT with two arms: EMDR (plus standard neuropsychiatric care (NPC)) and standard NPC only. The two groups will be compared at baseline (T0), 3 months (T1), 6 months (T2) and 9 months (T3). Fifty adult patients with a diagnosis of FND, confirmed by a neurologist according to standardised diagnostic criteria, will be recruited via a UK neuropsychiatry service. The research assistant (RA) and project statistician will be blind to treatment allocation. After the intervention period, semistructured interviews will be carried with a proportion of participants and clinicians to explore their experiences and views about the trial.

### Study setting

This is a single-site study being carried out at a neuropsychiatry service, based at St. George’s Hospital, Tooting, London, UK. The service is part of the South-West London and St. George’s Mental Health NHS Trust (SWLSTG).

### Public and patient involvement (PPI)

The research design has been informed by a PPI meeting (June 2020), where all five participants had lived experience of FND. Three PPI representatives join regular Trial Management Group (TMG) meetings, review participant literature and will coproduce the interview schedules and contribute to the design of a substantive study. Two PPI representatives join the Trial Steering Committee (TSC).

### Eligibility criteria

#### Inclusion criteria

1. Predominant diagnosis of FS and/or mFND, with diagnosis confirmed by neurologist.
2. Aged 18 years or over.
3. Capacity to consent.
4. Willingness to attend regular psychological therapy sessions.
5. Reporting at least one traumatic event on the International Trauma Exposure Measure (ITEM).

#### Exclusion criteria

1. Non-English speaking.
2. Current ongoing adversity that is likely to interfere with psychological therapy, for example, domestic violence, homelessness, unresolved compensation claim/litigation.
3. Predominant diagnosis of borderline personality disorder (comorbid diagnosis is acceptable, as long as FND is the predominant difficulty).
4. Predominant diagnosis of chronic pain condition (comorbid diagnosis is acceptable, as long as FND is the predominant difficulty), for example, fibromyalgia.
5. Predominant diagnosis of chronic fatigue syndrome (comorbid diagnosis is acceptable, as long as FND is the predominant difficulty).
6. Diagnosis of a psychotic disorder.
7. Diagnosis of dissociative identity disorder or score in clinical range on ‘identity disturbance’ subscale of Multiscale Dissociation inventory.
8. Uncontrolled epileptic seizures.
9. Diagnosis of an eating disorder.
10. Current severe self harm or strong suicidal ideation that requires secondary care mental health services input.
11. Current alcohol or drug harmful use or dependence.
12. Current diazepam use exceeding the equivalent of 10 mg per day.
13. Currently attending individual psychological therapy focused on FND or other specialist FND-specific treatment such as inpatient/outpatient multidisciplinary treatment or intensive FND-specific physiotherapy.

### Study interventions

#### EMDR plus standard NPC

The intervention group will be offered up to 16 EMDR sessions, and a 1-month follow-up session, as well as attending standard outpatient neuropsychiatric appointments. Participants will be given the choice of attending EMDR face-to-face or virtually via a video-consultation platform. Sessions will normally be attended weekly, with treatment completed within 6 months. Sessions will be 60–90 min long, in accordance with NICE guidance for PTSD.^33^ A minimum of eight sessions was chosen based on patient feedback and previous research.^34^ Optimum session duration and number will be examined as part of the trial. The optional follow-up session will occur 1 month after treatment completion.

EMDR for FND is a collaborative and individualised approach, following the standard EMDR protocol, but tailored for FND presentations. Treatment has three broad stages: assessment, psychoeducation, target selection and preparation for processing; processing of targets; and ending of therapy. The initial sessions incorporate education regarding FND, anxiety and dissociation; formulating collaboratively with the participant regarding the development of FND symptoms; and giving a rationale for EMDR. In collaboration with the participant, target memories/images will be chosen, such as: (1) distressing memories associated with the time when symptoms began; (2) distressing memories from past events that may be relevant to their FND symptoms; (3) FND symptoms themselves, when present in session, or an image of them; and (4) distressing images about the future, for example, image of having symptoms in front of others. EMDR therapy will follow the therapy protocol developed by the Chief Investigator (CI) for this study. Table 2 shows an overview of treatment, with a guide regarding how many sessions per stage.

**Table 2:** Overview of EMDR for FND

Session(s)	Overview of content
1–3	History taking and preparation for therapy (phase I–II of standard EMDR protocol) Formulating their FND presentation and increasing their understanding of FND Collaboratively selecting target memories/images
4–15*	Processing of EMDR targets using EMDR’s three-pronged approach of past, present and future targets
Final session	End therapy
	An optional follow-up session can be offered 1 month after completing therapy (this does not count as one of the 8–16 sessions)

*Total number of sessions can be between 8–16. If completing by session 8, there will be fewer sessions in this phase of the intervention.

#### Standard NPC

NPC is treatment-as-usual and will consist of 1–3 routine outpatient appointments with an assigned neuropsychiatrist in the trial period. Participants will not begin any FND-specific individual psychological therapy, inpatient/outpatient multidisciplinary treatment or intensive specialist FND-specific physiotherapy. If a participant begins FND-specific individual psychological or physiotherapy treatment in the trial period, they would not be able to complete the trial. Data from their last assessment point will be used for analysis. Participants can still attend psychoeducational interventions focused on FND that are administered by the service and remain part of the trial. Their assigned neuropsychiatrist can refer for psychological therapy outside of the service for any comorbid conditions, for example, therapy for depression.

### Training, supervision and fidelity checks

The study RA has received training to deliver screening interviews and collect data. The trial EMDR-trained psychological therapists have attended training on the FND-specific EMDR protocol, delivered by the chief investigator (SC). They receive regular clinical supervision from SC, as well as external supervision from an EMDR consultant (a requirement of offering EMDR). Therapists will complete a session record form after every session, and these will be reviewed in supervision to enhance fidelity.

All sessions of EMDR will be video-recorded and the recordings stored on the secure SWLSTG NHS server. The therapists will share access to the recordings with SC for supervision purposes, and excerpts will be shown in EMDR supervision. Randomly selected recordings of processing sessions will be rated for fidelity, using the EMDR Fidelity Rating Scale Version 2—Adverse Life Experiences Processing subscale, by an EMDR Consultant.^35^ These scores will be analysed to evaluate fidelity.

### Primary objectives and outcome measures

A mixed-methods approach will be used to establish feasibility and acceptability. The feasibility criteria and progression criteria are summarised in table 3.

**Table 3:** Feasibility criteria and progression criteria for MODIFI

Criterion	Critical feasibility outcome	Other feasibility and acceptability data relevant to the criterion	Proposed threshold on critical outcome
Recruitment rate	Percentage potentially eligible participants attending screening interview	Number of potentially eligible participants identified during neuropsychiatric assessment Number of participants who consent and are randomised Reasons for non-eligibility	Above 70% attending a screening interview of those approached to participate, and if attendance is less than 70%, ways to increase the screening interview attendance rate will be considered.* If 50%–70% attend a screening interview of those approached to participate, and if attendance is less than 70%, ways to increase the screening interview attendance rate will be considered.† If below 50% attend screening interview of those approached to participate, feasibility will not be demonstrated.‡
Intervention adherence	Percentage of participants randomised to EMDR+NPC who complete therapy (completion=attendance of 8 or more sessions, maximum session number=16)	Qualitative interviews with participants who attend EMDR Therapy session record forms Average number of sessions attended per course of therapy	Feasibility will be demonstrated if above 70% complete therapy (attend eight or more EMDR sessions).* If 50%–70% participants complete therapy, ways to improve engagement will be considered.† If <50% participants complete therapy, feasibility will not be demonstrated.‡
Outcome measurements completion	Percentage of participants who complete outcome measures at all time points	Retention of participants (rates of withdrawal across both arms) Reasons for withdrawal Qualitative interviews with participants	Feasibility will be demonstrated if above 70% of participants complete outcome measures at each time point.* If 50%–70% of participants complete outcome measures at all time points, ways to improve retention and completion of outcome measures will be considered.† If <50% of participants complete outcome measures at all time points, feasibility will not be demonstrated.‡

*Continue to main study without modifications.

†Future definitive trial is feasible with modifications.

‡Future definitive trial is not feasible.

EMDR, eye-movement desensitisation and reprocessing therapy; NPC, neuropsychiatric care.

Assessment of safety (adverse/serious adverse events) will be compared between the two arms. Therapy satisfaction and therapy fidelity will also be examined. The outcome measures used will be evaluated and the primary outcome measure identified, and the required sample size for a substantive RCT will be calculated.

The nested qualitative study will explore participants’ and treating therapists’ experiences and views. Information from these interviews will inform design and trial materials for a substantive study, including the FND-specific EMDR protocol. As the feasibility trial includes the option of attendance of appointments virtually, take up of this option will be measured.

### Outcome measures

In terms of assessing FND, there is no single validated outcome measure for FND symptoms available.^36^ Ecological Momentary Assessment (EMA) using the m-Path App will be used to assess FND symptoms. Participants will rate a maximum of two symptoms, chosen at the beginning of the trial period, for example, seizures, tremor, limb weakness, tingling/numbness, gait disturbance, for a 2-week period at each time point. For each of the two symptoms chosen, they will answer five questions daily (frequency, severity, interference, associated distress, associated preoccupation), and the mean for each item will be calculated for each 2-week period at each time point.

The schedule of enrolment, interventions and assessments for participants is illustrated in table 4. For descriptions of outcome measures, please see online supplemental material.

10.1136/bmjopen-2023-073727.supp1 Supplementary data

**Table 4:** Schedule of enrolment, interventions and assessments for participants

	Screening	Baseline (T0)	3 Months (T1)	6 Months (T2)	9 Months (T3)	Post-trial period
Enrolment						
Consent to contact obtained by clinical staff (neuropsychistrist)	x					
Contacted by RA to arrange screening interview	x					
International Trauma Exposure Measure (ITEM)^37^	x					
Multiscale Dissociation Inventory (MDI)^38^	x		x	x	x	
Informed consent	x					
Demographics recorded	x				x*	
History of psychological therapy		x				
Current medication		x				
Randomisation allocation		x				
Interventions						
EMDR		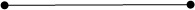				
NPC		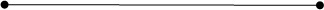				
Assessments						
WHO Disability Assessment Schedule (WHODAS 2.0)^46^		x	x	x	x	
EQ-5F-5L^47^		x	x	x	x	
Ecological momentary assessment of FND symptoms via m-Path App		x	x	x	x	
PHQ-9^48^		x	x	x	x	
GAD-7^49^		x	x	x	x	
International Trauma Questionnaire (ITQ)^50^		x	x	x	x	
Adult Service Use Schedule (AD-SUS)^51^		x			x	
Beliefs related to diagnosis and intervention		x			x	
Clinical Global Impression—Improvement Scale (CGI-I)^52^ rated by participant					x	
CGI-I rated by participant-nominated person					x	
Measure of satisfaction					x	
Review/reporting of patient AEs/SAEs		x	x	x	x	
Qualitative Interviews						
Informed consent for qualitative interview (select participants)						x
Qualitative interviews with select sample of participants and trial therapists						x

*Participants will be asked at T3 (end of trial period) whether there have been any changes regarding medication, relationship status and employment status and any changes recorded.

EMDR, eye movement desensitisation and reprocessing therapy; FND, functional neurological disorder; NPC, neuropsychiatric care; RA, research assistant.

### Recruitment and timeline for participants

#### Recruitment

Potential participants will initially be screened as part of routine neuropsychiatric appointments. The psychiatrist will verbally introduce the trial to potentially eligible participants and ask for permission for the RA to contact them. Potentially eligible participants’ names will be passed to the CI, who will check that they likely meet the eligibility criteria. The RA will provide potential participants with a summary of the study and will send the Participant Information Sheet and informed consent form for them to review (see online supplemental material). They will have the opportunity to ask any questions they may have. If willing to participate, the RA will obtain informed consent and arrange a screening interview.

The screening interview will be used to establish eligibility in terms of inclusion criterion “Reporting at least one traumatic event on the International Trauma Exposure Measure (ITEM)”, and exclusion criterion “Diagnosis of dissociative identity disorder or score in clinical range on ‘identity disturbance’ subscale of Multiscale Dissociation inventory (MDI)”.^37 38^ The (ITEM) will be used to assess previous adverse experiences. If they meet this inclusion criterion, potential participants will then complete the MDI, which will screen for clinical levels of dissociative ‘identity disturbance’. Potential participants need to score in the non-clinical range of the subscale ‘Identity Disturbance’ on the MDI to take part (score <15). Eligible participants will complete additional baseline measures, as well as completing demographic information, medical history and listing any previous psychological therapies attended for any difficulty (not just FND).

**Figure 1:**
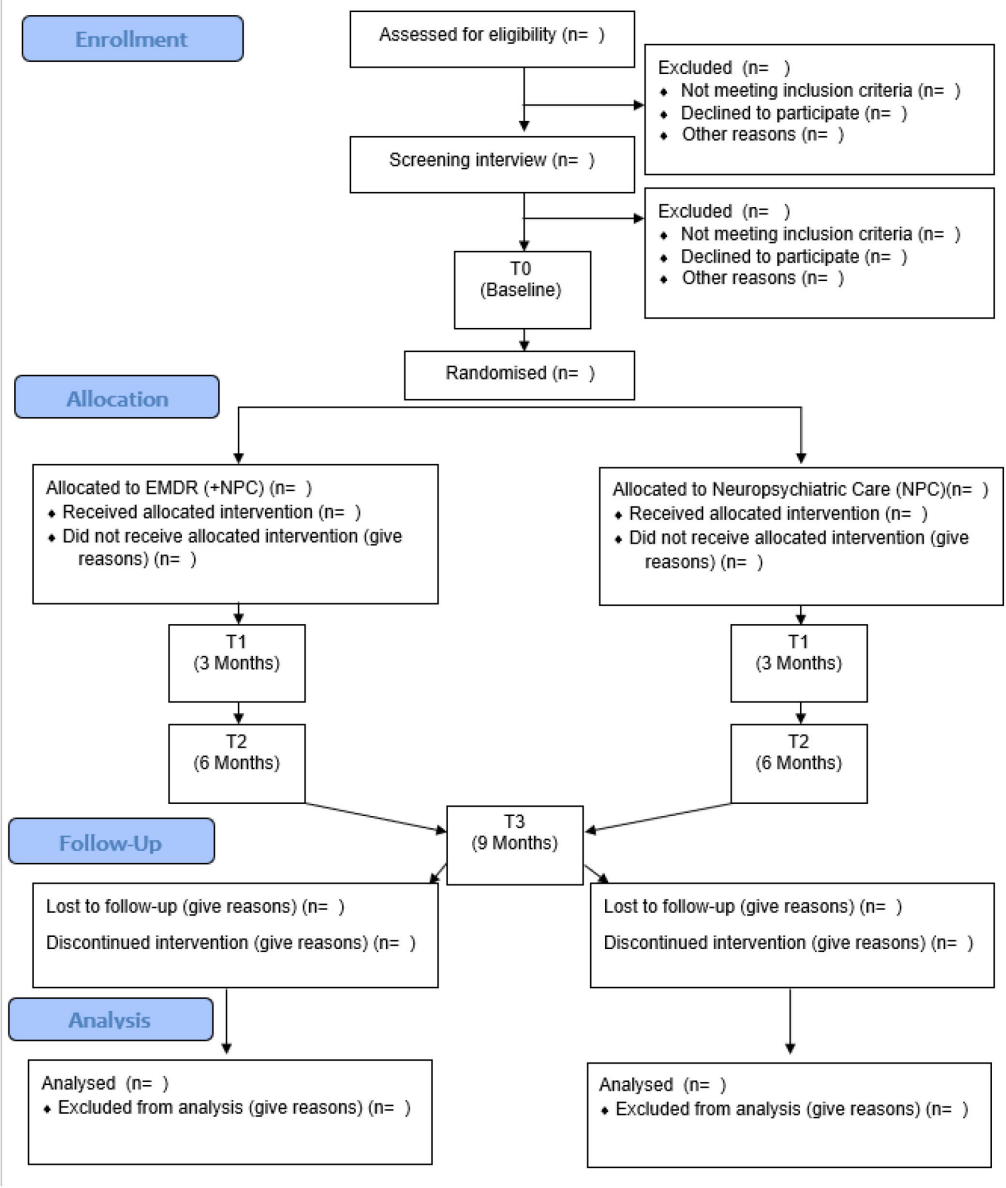
Consolidated Standards of Reporting Trials flow diagram for MODIFI study. NPC, neuropsychiatric care.

The flow of participants is illustrated in figure 1.

### Incentives

Participants will be reimbursed for research-related travel costs, up to the value of £20 per appointment (for those who choose to attend appointments in-person, rather than virtually). A non-contingent £25 incentive will be offered to participants for taking part 9 months after informed consent, unrelated to whether they complete the trial or not.

### Allocation and blinding

Consenting participants will be randomised into EMDR (plus NPC) or NPC in a 1:1 ratio. A stratified block randomisation (using randomly permuted blocks of sizes 2 and 4) will be used to ensure similar numbers of patients with and without PTSD symptoms (ie, meeting or not meeting PTSD diagnostic criteria as determined by diagnostic algorithm of the International Trauma Questionnaire (ITQ)) are (relatively) equal across arms. Randomisations will be carried out by the trial manager using the randomisation function on REDCap.

### Blinding

The trial is a single-blind trial with the RA and statistician remaining blind to treatment allocation. It is not possible to blind participants or treating clinicians to randomisation outcome.

### Analysis and statistical methods

#### Sample size

This is a feasibility trial; as such, a power calculation is neither possible nor necessary. Rather, the sample size is pragmatic. Target recruitment is 50 patients in total (25 in each arm), which is consistent with those recommended for pilot and feasibility studies to provide sufficiently reliable estimates of feasibility outcomes, for example, recruitment, adherence and attrition rates and adequate precision of means and variances to inform a fully powered RCT.^39–41^

#### Statistical analysis plan

Data analysis will follow a statistical analysis plan, formally agreed with the trial steering committee prior to analysis, and centred on describing key process measures to decide if a definitive trial is feasible. Participant throughput will be summarised in an extended CONSORT diagram (Eldridge *et al* 2016).^42^

Feasibility outcomes will be summarised using descriptive statistics, with 95% CIs provided to permit assumptions when planning the main trial. Data relating to (serious) adverse events, assessment, screening and recruitment logs will be used to produce accurate estimates of safety, eligibility, recruitment and consent rates in the study population. To determine the adequacy of study inclusion and exclusion criteria, and the generalisability of the trial to the FND population, baseline sociodemographic and clinical characteristics will be compared between study participants and ineligible and non-consenting patients. Intervention adherence (eg, EMDR session attendance) and satisfaction of care data will be used to contribute to the evaluation of the acceptability of allocated intervention/treatment arms and mean EMDR fidelity scores for rated sessions calculated to assess intervention fidelity. At each time point, retention rates will be estimated for each of the patient reported/clinical outcome measures, with consideration given to differential dropout between the arms of the trial, to identify potential (attrition) bias in treatment completion and/or data collection. EMA completion rates in each 2-week assessment period will be calculated with respect to daily assessment. All feasibility outcomes will be compared with relevant full-trial progression criteria.

Baseline characteristics will be reported according to treatment arm. Continuous variables will be reported as mean (SD) if normally distributed or median (interquartile range) if non-normal, while categorical variables will be presented as frequency (%). Subsequent analyses will summarise the proposed patient-reported and clinical outcomes (eg, quality of life and depression measures, (2-week mean) EMA symptom ratings) at each time point for each trial arm using appropriate descriptive statistics (eg, group mean, SD). To provide an indication of potential changes in scores/frequencies between the four time points, linear/logistic mixed-effects regression models will be employed performed on an intention-to-treat basis (accounting for data assumed to be missing at random). These random intercept (mixed) models will include intervention group, time and intervention group-by-time interaction. There will be no emphasis on hypothesis testing, however, which is reserved for the future main trial. Rather, pre-to-postintervention standardised effect sizes (Hedges’ g, relative risk) will be computed (SDs will be computed from estimated model standard errors) with associated CIs calculated to explore imprecision around effect sizes (Durlak 2009).^43^ Due to the small sample size, important covariates (eg, baseline score on relevant measure, gender, age) may be included in models if the two arms happen to be highly imbalanced. Additional analyses (using mixed effect models) focused on ‘per-protocol’ outcomes and the potential value of the (intensively) collected EMA data on FND symptoms will also be administered (see online supplemental material for additional detail).

A descriptive assessment of healthcare utilisation stratified by treatment arm will also be presented. The Adult Service Use Schedule (AD-SUS) will be used to record previous 6 months of health and social care resource use at baseline (T0) and 9 months of health and social care resource use at 9 months (T3). The acceptability of the AD-SUS will be assessed and key items of resource use for a future RCT will be identified. EQ-5D-5L utility scores will also be calculated. A cost-effectiveness analysis will not be conducted.

#### Qualitative analysis plan

A subsample of participants in EMDR+NPC (n=8) and NPC (n=6) arms of the trial will be invited for in-depth semistructured interviews after the intervention period. A sampling framework will be used that ensures participants are included that are representative of the sociodemographic characteristics and clinical profile (FS and FMD, presence of PTSD symptoms). Interviews will focus on the acceptability and feasibility of participating in a future larger trial of EMDR and explore experiences of recruitment practices, informed consent procedures, randomisation and range of outcomes measures. For participants in EMDR+NPC arm, interviews will also gauge the acceptability and perceived value of the EMDR intervention, suitability of number and frequency of sessions, ways of optimising engagement; and perceived benefits/limitations of the intervention as well as any recommendations for improvement. Final interview guides will be coproduced by the team, including PPI representatives.

Semistructured interviews will also be carried out with both treating EMDR therapists. They will explore therapists' views concerning the research design and EMDR intervention protocol, including their experiences of training and delivering EMDR to patients with FND, as well as their perceptions of its deliverability within the NHS.

Interviews will be carried out by an RA, recorded and transcribed verbatim, with transcripts cross-checked against the original recordings to ensure accuracy. They will be analysed using reflexive thematic analysis.^44 45^

### Trial status

Enrolment of the first participant occurred on 19 December 2022. The trial is ongoing and we anticipate completing recruitment by November 2023.

## Ethics and dissemination

### Research ethical approval

The research was reviewed by the NHS West Midlands—Edgbaston Research Ethics Committee with a favourable opinion (Reference: 22/WM/0178), and Health Research Authority approval has been received (both dated 27 September 2022).

### Informed consent

Participants will provide informed consent prior to attending the screening appointment. This includes consenting to be recorded if randomised to EMDR+NPC. For the subsample of participants in the nested qualitative study, additional consent will be obtained at the point of being invited for interview. Please see online supplemental material for additional detail.

### Confidentiality and management of participant data

All data will be pseudoanonymised and input on to the trial database, which will be saved on the secure NHS server. The Trial Master File will be backed up weekly on an encrypted hard drive. No paper copies will be stored. The data collection and management will be in line with GDPR Data Protection Act (2018). For details regarding data collection, data handling, and record keeping, refer to the Data Management Plan found in online supplemental material.

### Monitoring, audit and inspection

The trial will be monitored by the TMG and TSC. A Data Management Committee is not required as this is a feasibility study. The study will be self-monitored following a Monitoring Plan protocol. Please see online supplemental material for detailed information.

### Access to the final trial dataset

The CI and statistician will have access to the final trial dataset. If anyone else requires access, a request will need to be made via the TSC.

### Post-trial care

Participants who take part in the trial will have access to support from the neuropsychiatry service. Their care will be overseen by their allocated neuropsychiatrist and appropriate referrals made, if needed.

### Dissemination

Trial findings will be published in a peer-reviewed journal or platform within 24 months from study completion. Authorship will be determined in accordance with the ICMJE guidelines and other contributors will be acknowledged. Participants will be notified via email after the results have been published. Trial registries will be updated during the study and the trial protocol and key outcomes will be made publicly available within 12 months of study completion.

## Supplementary Material

Reviewer comments

Author's manuscript
