# Supplementary material for: MODIFI: protocol for randomised feasibility study of eye-movement desensitisation and reprocessing therapy (EMDR) for functional neurological disorder (FND)
Source: BMJ Open. 2023 Jun 2;13(6):e073727. doi: 10.1136/bmjopen-2023-073727 (PMC10255051; doi:10.1136/bmjopen-2023-073727)
Supplement: Supplementary data [file bmjopen-2023-073727supp001.pdf]

MODIFI Protocol – Supplementary Material

Contents

Outcome Measures 2
Additional details regarding Consent, Blinding, and Confidentiality 5
Additional details regarding Analysis and Statistical Methods 8
Recording and Reporting of Events 10
Monitoring, Audit & Inspection 12
Data Management Plan (DMP) 15
Participant Information Sheet
Participant Consent Form

Cope SR, *et al. BMJ Open* 2023; 13:e073727. doi: 10.1136/bmjopen-2023-073727

Outcome Measures

I. Health-Related Quality of Life/Functioning:

World Health Organisation Disability Assessment Schedule (WHODAS 2.0) (WHO, 2010) is a 36-item generic assessment instrument for health and disability. The questionnaire asks about difficulties due to health conditions and asks participants to rate these on a scale with 5 levels: None, Mild, Moderate, Severe, Extreme or cannot do.

EQ-5D-5L (Herdman et al., 2011) consists of a descriptive system assessing five dimensions: mobility, self-care, usual activities, pain/discomfort and anxiety/depression. Each dimension has 5 levels: no problems, slight problems, moderate problems, severe problems and extreme problems. It also has a self-rated health (0-100) visual analogue scale, where the endpoints are labelled 'The worst health you can imagine' (0) and 'The best health you can imagine' (100).

II. FND:

There is no single validated outcome measure for FND symptoms available (Pick et al., 2020). Ecological Momentary Assessment (EMA) using the m-Path App, which is well supported and maintained across all smartphone platforms, will be used to assess FND symptoms.

Participants will rate a maximum of 2 symptoms, chosen at the beginning of the trial period, e.g. seizures, tremor, limb weakness, tingling/numbness, and gait disturbance. A paper version will be available to participants if necessary. Participants will be encouraged to choose the 1 or 2 symptom(s) that currently have the worst impact on their quality of life.

Each participant would answer the following questions once a day over each 2-week time period for a maximum of two symptoms:

Select the symptom you are referring to [seizures, tremor, limb weakness, tingling/numbness, jerks, gait disturbance]. With this symptom in mind, please rate the following:

a. If the symptom is episodic (not present all the time), rate the number of times (frequency) it has occurred in the previous 24 hours, or answer not applicable (n/a) if your symptom is continuous

b. Over the last 24 hours, please rate the severity of the symptom, from 0 (none) – 10 (extremely severe). If the symptom has not occurred, rate 0 for this question.

c. Over the last 24 hours, please rate the extent the symptom has interfered with your daily functioning, from 0 (not at all) – 10 (extreme interference)

d. Over the last 24 hours, please rate the extent the symptom has distressed you, from scale 0 (none) – 10 (extreme distress)

e. Over the last 24 hours, please rate the extent the symptom has preoccupied you (been on your mind), from 0 (not at all) – 10 (all of the time)

For each of the 2 symptoms chosen, the mean for each item will be calculated for each 2-week period at each time point.

III. Depression and anxiety:

PHQ-9 (Kroenke & Spitzer, 2002) is a 9-item measure assessing presence and severity of depression. Each item is rated on a 4-point scale ranging from 'Not at all' to 'Nearly every day'.

GAD-7 (Spitzer et al., 2006) is a 7-item measure assessing presence and severity of anxiety. Each item is rated on a 4-point scale ranging from 'Not at all' to 'Nearly every day'.

IV. PTSD:

International Trauma Exposure Measure (ITEM) (Hyland et al., 2021) is a checklist developed to capture traumatic life events, and their associated features, in a manner consistent with the description of a trauma in the ICD-11 diagnostic manual. It assesses exposure to different traumatic life events across different developmental periods (childhood, adolescence, adulthood, and lifetime); frequency of exposure to one's most distressing traumatic event; and the main emotion associated with one's most distressing traumatic event.

International Trauma Questionnaire (ITQ) (Cloitre et al., 2018) is a valid and reliable measure that assesses PTSD and Complex PTSD in line with the ICD-11.

V. Dissociation:

Multiscale Dissociation Inventory (MDI) (Briere, 2002) is a 30-item self-report measure of dissociative experiences in the previous month. It measures six different types of dissociation: disengagement, depersonalisation, derealisation, emotional constriction, memory disturbance, and identity dissociation.

VI. Service utilisation and other cost variables:

Adult Service Use Schedule (AD-SUS) (Tyrer et al., 2014). The AD-SUS was developed by an economist for previous work in similar populations and was adapted for the needs of this evaluation. It assesses employment, healthcare utilisation and medication.

VIII. Improvement:

Clinical Global Impression – Improvement Scale (CGI-I) rated by participant (single item, 7-point scale) (Guy, 2000)

CGI-I rated by person nominated by participant (e.g. family member, partner, close friend, carer) (single item, 7-point scale)

IX. Beliefs about diagnosis and intervention:

Agreement with diagnosis of FND (single item, 11-point scale)

Preference regarding treatment (EMDR + NPC, NPC or no preference)

Belief in having been given the right treatment (single item, 11-point scale)

X. Measure of satisfaction

Satisfaction rating of treatment (single-item, 11-point scale)

Cope SR, *et al. BMJ Open* 2023; 13:e073727. doi: 10.1136/bmjopen-2023-073727

Additional details regarding Consent, Blinding, and Confidentiality

The RA will assess risk and capacity to provide consent throughout the screening and assessment procedures, with input from the Chief Investigator, who is a clinical psychologist. The limits of confidentiality will be made clear to participants. There will be a standard protocol for assessing and managing safeguarding and/or risk disclosures, which will follow safeguarding relevant policies and procedures of SWLSTG Mental Health NHS Trust.

Those not eligible to take part in the research due to not endorsing experiencing any adverse events or experiencing high identity disturbance, and who are interested in the option of attending psychological therapy, will be offered an appointment with the CI (who is also the team's lead clinical psychologist) to discuss treatment options local to them or within the service. This will mean if non-eligible participants are distressed by non-inclusion, the RA will be able to offer them an appointment with a clinical member of staff.

Anonymised information on participants who are not randomised will include demographic data, diagnoses, type and duration of FND symptoms, the reason not eligible for trial participation, or if they are eligible but declined.

Consent

Participants will consent by one of the following:

Face to face with the RA. The participant and RA will sign the consent form. The RA will scan the original and save a copy in the electronic site file, upload a copy to the patient's electronic notes, and give a copy of the form to the participant. The paper form will be shredded.

Remote consent, whereby the consent form is shared electronically and the participant signs electronically and returns the form to the RA, who will then sign the form themselves and send a fully signed copy to the participant. The RA will file the original in the Trial Master File (TMF) and upload a copy to the patient's electronic notes.

The RA who will be responsible for taking written informed consent will have received training in Good Clinical Practice (GCP) alongside internal additional training by the study team.

Additional consent provisions for collection and use of participant data

All trial participants will be asked whether they would be willing to be contacted at a later stage for participating in further studies related to the trial. This consent to contact will include the qualitative interview. It will be made clear that this is optional and that declining consent to be contacted for participation in additional studies will not prevent them from taking part in the trial.

For the qualitative interview, it will also be made clear that not everyone will be contacted, since we will only be recruiting a sub-sample. If a participant indicates willingness, the RA will contact them directly once all participants have completed the study period, and all

Cope SR, *et al. BMJ Open* 2023; 13:e073727. doi: 10.1136/bmjopen-2023-073727

quantitative data has been collected. Written consent will be obtained from those consenting to interview, and the form will be stored as above.

EMDR Therapists will also provide written informed consent to participate in interviews. The signed form will be filed in the site file, and a copy of the form will be given to the therapist.

Additional details regarding blinding

Participants will be reminded, when the RA meets with them to complete measures, that the RA must not be told which arm they have been randomised to. The TM will input which arm (Group 1 or Group 2) each participant was allocated to after all participants have completed their time in the study and the data has been inputted, but they will not disclose whether Group 1/2 refers to EMDR+NPC or NPC, so that the statistician will remain blind to treatment allocation whilst carrying out data analysis.

Long-term follow-up

As part of informed consent, participants will be asked to consent to open-label follow-up to potentially provide additional data for a prospective substantive RCT.

Withdrawal criteria

It will be made clear to participants that they have the right to withdraw from the study at any time for any reason, without the need to justify their decision, and that it will not affect their routine care. The investigator also has the right to withdraw participants from the study in the event of clinical contra-indications. Should a participant withdraw from therapy only but not from the study, efforts will be made to continue to obtain follow-up data, with the permission of the participant. Should a participant withdraw from the study, we will still use any previous data collected from that participant up to the point of withdrawal, but will make no further attempt to contact or collect data

Data protection and patient confidentiality

Clinical confidentiality

During the screening interview, potential participants will be advised of the limits of confidentiality (i.e. that the researcher will have a duty to inform health professionals if the participant discloses information any safeguarding or risk issues). It is also possible that disclosure of significant criminal acts potentially requiring action will occur during assessment and therapy sessions. The research team will follow the protocol for Assessing and Managing Safeguarding and Risk Disclosures. The RA and trial therapists will seek supervision and ensure appropriate action is taken as soon as possible. The limits of confidentiality and possibility of action arising from certain disclosures will be noted in the Participant Information Sheet (PIS). Potential participants will be offered at least 24 hours to consider all the information provided before written consent is obtained. Therapists will discuss the limits of confidentiality with participants allocated to the EMDR+NPC at the start of therapy, and at any appropriate subsequent points during the therapy.

Cope SR, *et al. BMJ Open* 2023; 13:e073727. doi: 10.1136/bmjopen-2023-073727

Data confidentiality

Research data will be confidential unless a participant discloses information that indicates that they or another person are at risk of harm (see clinical confidentiality section above). All participants will be informed of this during the written informed consent process.

All data will be pseudonymised. Each participant will be assigned a unique participant number when they are referred to the trial, which will remain the same for the duration of their involvement in the trial. This number will be recorded on all eligibility measures, forms and databases used to record data on participants. An electronic record sheet linking participant identity, contact details, and trial identification number will be created and saved on the secure Trust drive. The password will only be shared with the research team. All data will be kept secure at all times and maintained in accordance with General Data Protection Regulation (GDPR, 2018) requirements and archived according to clinical trial GCP regulations. Participant consent forms will be retained electronically, kept confidential and stored securely.

Only the RA, TM and chief investigator will have access to the trial database. The statistician will receive a copy of the database once data collection is complete and the database is locked.

Therapy notes will be recorded on Rio, the electronic clinical record system used by SWLSTG, in line with Trust procedures. The RA will access the system when a participant referral is received, but will not need to access the system thereafter, so will not view participants' clinical records after recruitment to the study.

Recordings

All therapy sessions (with participant consent) will be recorded to monitor the fidelity of the intervention delivery. When sessions are held online via MS Teams, recording will take place via the platform. When sessions are held in-person, clinicians will record each session by using MS Teams too (they will position their laptop so both participant and clinician can be viewed). Each recording file will be named with participant identifier and date, and will be stored on the secure SWLSTG NHS server. Recordings of the therapy will be accessible to the participant's therapist, the CI, and excerpts will be shown in clinical supervision. A random selection of processing sessions will be shown to an EMDR Consultant in order to rate the sessions for fidelity. The video recordings are only being used to enhance fidelity to the therapy protocol, and they will be deleted after a random selection of them has been rated for fidelity.

Interviews will be held remotely via MS Teams or in-person. Recordings will take place via MS Teams in both instances with the video recording function turned off (only audio will be captured). All recordings will be labelled and stored securely on the Trust server. All audio recordings will be deleted after transcription has been completed. The transcriptions will be stored in the TMF, and stored for a minimum of 10 years in line with the other essential data.

Additional details regarding Analysis and Statistical Methods

Per-protocol analyses of outcome measures

Exploratory analyses using mixed effect models will examine the rate of change in intervention and control groups on outcome measures across four time-points, adjusting for relevant baseline scores and variables of interest (predominant symptom, presence of PTSD, levels of dissociation), and investigate changes on a per-protocol basis (focused on intervention adherence; i.e. including only participants who attended at least 8 sessions and with post-treatment data).

EMA data

The potential value of the (intensively) collected EMA data on FND symptoms will also be considered. EMA completion rates in each 2-week assessment period will be calculated with respect to daily assessment (total days with a completed assessment divided by total number of scheduled assessments); rates will be calculated for all assessments combined and for each assessment separately (symptom frequency, severity, and symptom-associated interference, preoccupation and distress) and the proportion of participants completing at least 33% of the total number of EMAs will be recorded.

Within participant day-to-day variability of repeated FND symptom ratings (e.g., frequency, severity) and Cronbach's alphas for EMA items (symptom severity, interference, distress, preoccupation) will be calculated at each time point to explore reliability and internal consistency of the EMA App, while exploratory mixed effect modelling analyses will adopt a three-level structure (repeated measurements at each EMA in addition to time point and participant). To explore relationships of EMA App data with other clinical measures, bivariate correlations (Pearson r or Spearman rho, according to data distribution) will be completed between 2-week mean EMA symptom ratings and corresponding WHODAS scores and between change in 2-week mean EMA symptom ratings and patient and clinician-rated CGI-I scales/change in WHODAS across corresponding time points. These analyses are expected to inform the analytic plan for a future larger study.

Procedure(s) to account for missing or spurious data

All available data will be included in the data listings and tabulations. If data exhibit missing or spurious values, aspects related to their collection, recording, and analysis will be investigated. The frequency (percentage) of participants with missing outcome values at each time point (retention rates), in addition to the reasons for missingness (where available), will be summarised. For (continuous/categorical) secondary outcomes, intention to treat (ITT) (linear/logistic) mixed effect models with maximum likelihood estimation will examine the rate of change in intervention and control groups on outcome measures across three post-treatment time-points, accounting for data missing at random. Given the feasibility status of the study, a full sensitivity analysis testing various missing data assumptions is not viable. Nevertheless, we will administer additional analyses imputing values for missing data using a conservative last observation carried forward (LOCF) procedure (in these analyses, where data is missing from assessment, a first observation carried backwards (FOCB) procedure will also be adopted). Safety and adverse/unwanted effects will be reported on an

as-treated basis (as opposed to an ITT approach), as these are best considered using the most accurate information.

Recording and Reporting of Events

The recording and reporting of AEs will follow the "Recording and reporting of (S)AEs protocol".

Operational definitions for AEs and SAEs

Adverse Events (AEs) are defined by the Health Research Authority (HRA) as any untoward medical occurrence, unintended disease or injury, or untoward clinical signs in participants, whether or not related to the treatment. In addition, issues specific to psychological therapies will also be monitored, namely: clinically significant increases in mental health or physical health problems; risky or problematic behaviours; harm to self/others, including suicide attempts; harm from others; and emergency room visits or crises. Clinically significant increases will be operationalised as an unresolved exacerbation requiring increased involvement from the care team, e.g. alterations to treatment plan. Distress or complaints associated with therapy, completion of assessment measures, or any other trial procedure would also constitute AEs.

The causes for the AEs will also be recorded and monitored. For each AE, the following potential reasons will be identified:

- Victimisation: aggressive behaviour, sexual abuse/assault, physical abuse/assault, emotional abuse/psychological maltreatment, exploitation, and other victimisation
- Mental health/psychological difficulties: excessive use of substances, exacerbation of mental health difficulties, suicidal ideation, and other psychological difficulties
- Trial procedures: group allocation, assessments, or therapy
- Physical health, including exacerbation of presenting FND symptoms, new neurological or other physical symptoms, and COVID-19 infection.
- Accidents or natural disasters
- Other.

AEs will be initially assessed at three levels of severity; mild, moderate and severe, which reflect the impact of the event on the person at the time. Please note there is a distinction between "severe" and "serious". Seriousness is the criteria for defining regulatory reporting obligations:

Serious Adverse Events (SAEs) are defined as:

- Death and life-threatening events (Category A)
- Incidents which acutely jeopardise the health or psychological wellbeing of the individual, resulting in immediate hospital admission and/or persistent or significant disability or incapacity (category B)
- Resulting in injury requiring immediate medical attention (category C).

Cope SR, *et al. BMJ Open* 2023; 13:e073727. doi: 10.1136/bmjopen-2023-073727

All AEs and SAEs will be reported to the TM and CI. A summary of (S)AEs will be presented at each Trial Management Group (TMG) and Trial Steering Committee (TSC) meeting. Urgent actions concerning participant and staff safety, communication with others, and clinical care will be immediately addressed by the CI and reported to the TMG.

AEs will be categorised for severity and seriousness by the TM and CI. SAEs will be further reviewed for relatedness to trial procedures and unexpectedness by the CI initially, and additionally by the chair of the TSC.

Relatedness and unexpectedness of an event to the intervention will be judged based on the following:

1. Related: the event resulted from administration of any of the research procedures, judged according to a temporal relationship (i.e., Serious Related Events; SREs);
2. Unexpected: the event is unexpected or unexplained given the participant's clinical course, previous conditions and history, and concomitant treatments (i.e., Unexpected Serious Related Events; USREs)

Recording and reporting of (S)AEs

Best practice, professional guideline and local NHS policies for monitoring mental state and risk will be followed throughout the participants' involvement in the trial and will be facilitated by the trial being embedded in the Neuropsychiatry Service. The safety of the intervention will be monitored closely during therapy sessions and through contacts with the Neuropsychiatry Service.

The occurrence of AEs will be monitored actively and systematically and recorded by the RA, therapists, and clinical team. All AEs and participant withdrawals will be recorded and monitored by the trial team. If indicated, the TM and CI will review the clinical notes and contact clinicians for any important additional information. At the completion of the trial, all clinical notes will additionally be checked, for the total duration of enrolment, for any previously undisclosed record of AEs. This extra procedure is to ensure completeness of records and to address the possibility of an increased likelihood of disclosure of AEs in the EMDR+NPC condition, as a result of greater frequency of contact and the therapeutic relationship. In the final reports of the trial, the numbers, types and severity of AEs by trial condition, as well as discontinuations, will be reported.

Notification of deaths

All deaths will be reported to the sponsor within one month regardless of whether the death is related to the trial, disease or an unrelated event.

Monitoring, Audit & Inspection

A trial manager is in post to coordinate the day-to-day running of the trial.

Trial Management Group (TMG):

Dr Sarah Cope – Chief investigator

Prof. Mark Edwards – Co-investigator

Dr Sharif El-Leithy – Co-investigator

Dr Jared Smith – Co-investigator

Dawn Golder – PPI representative

Dr Patricia Hogwood – PPI representative

Kati Jane Turner – PPI representative

Dr Serena Vanzan – Trial Manager

Caitlin Pentland – Research Assistant

A Sponsor representative will observe each meeting.

TMG Terms of Reference

1. To review and provide feedback on the study protocol, outcome measures and recruitment documents (including any amendments)
2. To support the progress of the trial towards its interim and overall objectives and against the study timescale
3. To agree on a trial monitoring plan
4. To advise on participant recruitment and retention
5. To facilitate data analysis
6. To support the troubleshooting of any hindrances to the timely completion of the trial
7. To review the study outcomes
8. To discuss future developments of the study
9. To report to the Trial Steering Committee (TSC)

Trial Steering Committee (TSC)

The TSC will oversee the study on behalf of the of the trial Sponsor and Funder and ensure that the study is conducted within appropriate NHS and professional ethical guidelines. It will provide advice on all appropriate aspects of the project; will oversee progress of the trial, adherence to the protocol, participant safety and the consideration of new information of relevance to the research question; will ensure the rights, safety and well-being of the

participants are given the most important considerations; will ensure appropriate ethical and other approvals are obtained in line with the project plan; will agree proposals for substantial protocol amendments and provide advice to the Sponsor and Funder regarding approvals of such amendments.

The TSC comprises of:

Dr Tim Nicholson– Independent Chairman

Prof Richard Brown – Independent Expert

Dr Sarah Cope – Chief investigator

Dr Serena Vanzan – Trial Manager

Steve Portelly – Independent PPI representative

Kirsty Griffin – Independent PPI representative

A Sponsor representative will observe each TSC meeting.

TSC Terms of Reference

1. To review and approve the study protocol and recruitment documents (including any amendments)
2. To monitor and supervise the progress of the trial towards its interim and overall objectives and against the study timescale
3. To ensure all trial activities are conducted in line with GCP guidelines
4. To inform the funder annually on the progress of the trial
5. To advise the funder on publicity and the presentation of all aspects of the trial

Protocol compliance

A study related deviation is a departure from the ethically approved study protocol or other study document or process (e.g. consent process or administration of study intervention) or from Good Clinical Practice (GCP) or any applicable regulatory requirements. Any deviations from the protocol will be documented in a protocol deviation form and filed in the study master file.

The reporting procedure for any potential protocol breaches will be reported to the CI and sponsor. Any potential breach will be assessed by the CI and their supervisor(s), and reported as below where this may be a potential serious breach.

Notification of Serious Breaches to GCP and/or the protocol

A “serious breach” is a breach of the protocol or of the conditions or principles of Good Clinical Practice which is likely to affect to a significant degree: (a) the safety or physical or mental integrity of the trial subjects; or (b) the scientific value of the research.

Cope SR, *et al. BMJ Open* 2023; 13:e073727. doi: 10.1136/bmjopen-2023-073727

In the event that a serious breach is suspected, the Sponsor will be contacted within 1 working day. In collaboration with the CI, the serious breach will be reviewed by the Sponsor and, if appropriate, the Sponsor will report it to the approving REC committee and the relevant NHS host organisation within seven calendar days.

Amendments

In the event of the sponsor wishing to make substantial amendments to the REC application or any supporting documentation, they will submit a valid notice of amendment to the REC for consideration.

The SWLSTG NHS and R&D departments will be notified.

The sponsor in collaboration with CI and investigators who contributed to the protocol will determine whether an amendment to the protocol is required and is substantial.

The CI will seek guidance from the local REC office. Changes that are deemed to be substantial will be communicated to the REC and SWLSTG NHS R&D department via the notification system in IRAS.

Any amendments will constitute a new approved version and will have a new label and date in order to distinguish it from the past protocol.

Data Management Plan (DMP)

Randomised feasibility study of eye movement desensitisation and reprocessing therapy (EMDR) for functional neurological disorder (FND) (MODIFI) COPS1001

DMP SIGN-OFF SHEET

**Trial Forms**	**Version Number**	**Version Date**
MODIFI_Research protocol_v3.1_23Nov2022	3.1	23/11/2022
MODIFI_Participant invite letter_v1.2_23Nov2022	1.1	23/11/2022
MODIFI_Consent Form_v1.3_23Nov2022	1.3	23/11/2022
MODIFI_PIS_v3.1_23Nov2022	3.1	23/11/2022
MODIFI_GP letter_v2.0_07Sep2022	2.0	07/09/2022

I have reviewed the trial's DMP and approved the use of the above documents

**Name**	**Position**	**Signature**	**Date**
Sarah Cope	Chief Investigator	[illegible]	04/11/2022
Serena Vanzan	Trial Manager	[illegible]	03/11/2022
Caitlin Pentland	Research Assistant	Caitlin Pentland	09/11/2022
Jared Smith	Trial Statistician	[illegible]	31/01/2023

Comments

Cope SR, *et al. BMJ Open* 2023; 13:e073727. doi: 10.1136/bmjopen-2023-073727

Randomised feasibility study of eye movement desensitisation and reprocessing therapy (EMDR) for functional neurological disorder (FND) (MODIFI)

COPS1001

1. Trial information

Trial type	Non-CTIPM Intervention - Feasibility
Study Sites	Single-site (SWLSTG)
Total sample size	50
Total duration of study<br><br>(months)	Trial start date _01/06/2022__<br><br>Planned duration for recruitment (months)___12____<br><br>Planned duration of follow-up (months)___9____<br><br>Total Duration ___36____Months
Study objective and design	The study aims to evaluate the possibility of delivering, and potential benefit, of EMDR for FND.<br>We will recruit 50 participants who have specific functional neurological symptoms: weakness, walking difficulties, jerks, shaking, and/or seizures from a Neuropsychiatry Service. Participants will be randomly allocated to EMDR, and routine medical appointments, or routine medical appointments alone. Those allocated to EMDR will be offered 8-16 weekly therapy sessions, completed within 6 months, and a follow-up session 1 month after therapy has ended. Participants will be able to choose whether they attend therapy in-person or via an online video conferencing platform. Participants will complete questionnaires regarding their health-related functioning, FND, mental health, and healthcare utilisation. These questionnaires will be completed at the beginning, and at 3 months, 6 months, and 9 months.<br>Some participants will attend interviews about their experiences of treatment.
Primary outcome measure	This is a feasibility study so no primary outcome measure has been chosen, as one of the study's aims is to investigate the value of a range of outcome measures, to determine the outcome measure with greatest symptom improvement and the required sample size, for a

	substantive RCT.
Secondary outcome measures	Health-Related Quality of Life/Functioning<br>FND<br>Depression and anxiety<br>PTSD<br>Dissociation<br>Service utilisation and other cost variables<br>Improvement<br>Beliefs about diagnosis and intervention<br>Measure of satisfaction
Interim analysis	Yes/**no**, if yes when_____________(Months from baseline)

2. Trial personnel and contact details

This section details the name, their position in the trial, email address, telephone/fax number for all staff involved in the trial including the sponsor. The trial coordinator/trial manager, the investigators, study staff involved in the data management (including computing staff responsibilities for maintaining hardware and software), the monitors and anyone else associated with the trial at each site.

2.1 Sponsor site personnel (add or remove accordingly)

Role	Name	Organisation	Contact Details
Sponsor	SWLSTG R&D	SWLSTG	ResearchDevelopment@swlstg.nhs.uk
Chief Investigator	Sarah Cope	SWLSTG	Sarah.Cope@swlstg.nhs.uk
Trial Manager	Serena Vanzan	SWLSTG	Serena.Vanzan@swlstg.nhs.uk
Research Assistant	Caitlin Pentland	SWLSTG	Caitlin.Pentland@swlstg.nhs.uk
Co-Investigator and Trial statistician	Jared Smith	SGUL	jasmith@sgul.ac.uk
Co-Investigator	Sharif El Leithy	SWLSTG	Sharif.Elleithy@swlstg.nhs.uk
Co-Investigator	Mark Edwards	KCL	mark.j.edwards@kcl.ac.uk
Primary contact for DM issues	Serena Vanzan	SWLSTG	Serena.Vanzan@swlstg.nhs.uk
Secondary contact for DM issues	Sarah Cope	SWLSTG	Sarah.Cope@swlstg.nhs.uk

3. Milestones

3.1 Study Milestones

Milestone	Date/Estimated Date
Date funding confirmed	22.03.2021
Date and version number of approved protocol	05.10.2022, v3.0
Date and version number of final protocol amendment(s)	03.11.2022, Am01
Date/version number of final approved CRF	No approval required
Release date/version number of final database	01.12.2022
Date DMP signed off	31.01.2023
Date of first participant first visit (FPFV)	19.12.2022
Date last participant last visit (LPLV)	

3.2 Proposed Data Milestones

Milestone	Date/Estimated Date
Data entry to commence	w/s 07.11.2022
Date of interim data partial lock if applicable	n/a
Data entry completed	Est Jun 2024
Last query resolved in the system	Est Aug 2024
Date of database final lock	Est Aug 2024

4. Data collection & data entry system

4.1 *Detail how data will be collected and entered from each site, whether to **complete the paper CRFs** or how to enter data electronically from each site.*
CRFs and outcome measures (questionnaires) will be completed electronically via the software REDCap, either by the RA (CRFs) or the participant (questionnaires). Where the participant will prefer paper questionnaires, these will be provided and returned via post, and the RA will enter the responses onto REDCap. If the participant requires face-to-face appointments, the RA will complete the CRFs and questionnaires either directly on REDCap or, if this is not possible, on paper and will then transfer the data onto REDCap.
Participants will also answer a few questions about their symptoms via the m-Path app.

4.2 *Provide details on the system used for data entry*
The REDCap online software will be used for collecting CRF and quantitative data. The m-Path app will be used for collecting information about participants' main symptoms (participant-completed).

4.3 *Outline who will be conducting the data entry from each site?*
The RA and the participants themselves.

4.4 *Outline whether single or double data entry will be carried out for all sites*
Single.

Cope SR, *et al. BMJ Open* 2023; 13:e073727. doi: 10.1136/bmjopen-2023-073727

4.5 *If double data entry is required describe the process and whether a 100% of CRFs or a sample of CRFs will be double entered. Outline how the two entries will be compared, who will carry out the comparison and how the results will be dealt with.*
N/a

4.6 *Outline the checks undertaken for outcome measures*
The TM will perform regular self-monitoring visits in which the data entered for a proportion of participants will be checked for completeness, accuracy and timely completion. The dataset from the m-Path app will be downloaded and backed up weekly by the RA, and will be inspected by the TM during monitoring visits.

4.7 *Provide details on how the data will be centralised*
N/a – one site.

5. Data checks & data validation for each site

5.1 *Outline who will perform data cleaning, missing data checks, consistency checks, range checks and logic, and how these are checked at each site?*
The TM will perform regular self-monitoring visits in which the data entered for a proportion of participants will be checked for completeness, accuracy and timely completion. In addition, value range restrictions will be applied in REDCap and the m-Path app so that no values outside the available range can be entered when completing closed questions.

5.2 *Describe how regularly the data will be checked*
Monthly.

5.3 *Provide contact person for each site for data queries if data managed centrally*
N/a

5.4 *Describe the data flow from each site to central data centre, and who will conduct the overall data check*
N/a – one site.

5.5 *Detail the **flow of the data** from the field to the final storage for each site*
Data will be collected directly on study database (REDCap) or m-Path app. Any data from paper version of the CRFs and questionnaires will be entered on REDCap by the RA within 24 hours of collection.

6. AE and SAE data handling

Outline how the AE data will be collected by each site and collated for all sites at the end of the trial.
N/a – one site.

7. Partial and/or final data check and database lock

Detail if an interim analysis is planned in the trial protocol, stating the time point and whether the database will be partially locked for the interim analysis.
N/a

Detail the process for partial and final data checks and data lock, outline who checks the data and who will sign off for partial/final data lock form(s)?

Cope SR, *et al. BMJ Open* 2023; 13:e073727. doi: 10.1136/bmjopen-2023-073727

The database will be locked after the following actions have been confirmed as completed by the TM:

1. All CRF data have been collected and entered onto REDCap.
2. All queries identified during regular data checks and self-monitoring visits have been resolved/clarified.
3. All missing information has been confirmed as being not available (as opposed to not entered).
4. All monitoring visits (incl. close-out) have been performed, and outstanding actions completed.
5. A final data quality check has been performed.

The TM will be responsible for locking the REDCap database (notifying the CI and Sponsor), send the REDCap and m-Path datasets to the trial statistician (with treatment allocation coded to prevent unblinding), and save a copy of the full databases in the TMF.

8. Data security and transmission between sites

Provide details on the data security procedures for transmitting data between sites
To comply with SWLSTG's Information Governance regulations, the datasets will be sent via an encrypted email to the trial statistician.

9. Data export & analysis

Explain how data will be exported and who the data should be sent to for data analysis.
The REDCap database with demographic and questionnaire data and data from m-Path app will be exported in CSV format and sent via encrypted email to Jared Smith, Trial Statistician and Co-investigator.

10. Data Back-up and archiving

Describe procedures in place to ensure data protection including back-up system (if you don't do this you could lose the data!)
The REDCap database and m-Path data will be downloaded and backed up weekly on a Trust external hard drive. The TMF will be saved on the Trust's shared drive, which is backed up every night, in a restricted folder dedicated to the study. In addition, the TMF will also be backed up once a week on the Trust encrypted external hard drive.
The study will be archived by the R&D team on the SWLSTG R&D shared drive.

South West London and St George's Mental Health NHS Trust

Participant Information Sheet

MODIFI: A feasibility study of eye MOvement DesensItisation and reprocessing therapy (EMDR) for FunctIonal neurological disorder (FND)

We would like to invite you to take part in this research. Before you decide whether you want to, it is important for you to read the following information carefully and discuss it with others if you wish. Please ask us if there is anything that is not clear or if you would like more information.

Brief summary

This research aims to evaluate the possibility of delivering, and potential benefit, of eye movement desensitisation and reprocessing therapy (EMDR) as a psychological therapy for functional neurological disorder (FND). This is a feasibility study which means that it will not have enough participants to demonstrate statistically whether EMDR is helpful or not, but it will test out the study procedures (such as recruitment and therapy attendance) and look at whether it seems to benefit participants or not. If the study shows that it is feasible and potentially beneficial, a larger trial will be designed. A larger trial will be able to find out whether receiving EMDR, in addition to routine neuropsychiatric care, is more helpful to patients than neuropsychiatric care alone. In order to receive funding for a larger trial, this feasibility study needs to be successful.

The feasibility study will recruit 50 participants with specific functional neurological symptoms (weakness, walking difficulties, jerks, shaking, and/or seizures) from the Neuropsychiatry Service at St. George's Hospital. Participants will be allocated randomly (i.e. by chance, using a computer programme) to either EMDR and routine neuropsychiatry appointments; or to routine neuropsychiatry appointments alone.

Background

Functional Neurological Disorder (FND) is a problem with the functioning of the nervous system and how the brain and body send and receive signals, rather than due to neurological disease or injury. FND causes a range of neurological symptoms, such as seizures, shaking, weakness, and paralysis. The symptoms are associated with significant distress and disability. Treatment for FND in the United Kingdom is limited, and the evidence-base for treatment is poor, despite it being a common condition.

A psychological therapy called cognitive-behavioural therapy (also commonly known as CBT) has been found to be beneficial, but it does not help everyone. EMDR is a psychological therapy that has combined ideas from several therapies, including cognitive-behavioural, psychodynamic, and experiential. The basic idea behind EMDR is that psychological distress originates from upsetting memories in a person's past, and that targeting those key memories will result in a reduction of distress. EMDR is a well-established treatment for PTSD, but there is increasing evidence that is also helpful for many other difficulties, including depression, anxiety disorders, tinnitus, and chronic pain. There is a small amount of case study evidence that EMDR can be useful for treating FND, but proper scientific testing is needed.

EMDR should only be delivered by suitably qualified psychologists or psychological therapists, who have undergone the EMDR training required and who receive EMDR supervision. Within EMDR, key memories or images are identified that could be targeted in therapy. Each EMDR processing session follows a standardised protocol. As part of this protocol, a patient is asked to bring the target image to mind, with an identified negative cognition, notice where they are feeling the distress in their body, and follow the clinician's fingers with their eyes. After each set of eye movements, they are asked what they noticed, and importantly, without discussion, they are told to "go with that" alongside the eye movements. It may involve multiple sessions for the distress to reduce sufficiently. It should be

South West London and St George's Mental Health NHS Trust

noted that alternative "bilateral stimulation" tasks, instead of eye movements, can be used, such as alternate tapping or audio-tones. There are several theories regarding why the eye movements, or dual-attention on another task, may facilitate emotional processing, but like other psychological therapies, the specific mechanisms of how it works are not clear. It may be that the eye movements (or other task) allow the person to stay in an optimal zone for emotional processing (i.e. not too distressed). Another theory is that engaging in two simultaneous tasks taxes working memory, which has limited capacity. Therefore "bilateral stimulation" whilst focusing on the target memory/image results in less space for the distressing memory/image, and it is stored back in memory less vividly with reduced disturbance.

Why have I been chosen?

We are recruiting people diagnosed with FND by a neurologist, who have been referred to the Neuropsychiatry Service. During the appointment you attended with a neuropsychiatrist, they identified that you experienced specific functional neurological symptoms, such as weakness, walking difficulties, jerks, shaking, and/or seizures, and that you were likely suitable to take part in this study. They asked you whether you would be willing to be contacted about the study, and following your consent, passed your contact details on to the research team.

What will I have to do?

If you are interested in taking part in the study, please let the research team know by emailing modifi.recruitment@swlstg.nhs.uk uk or telephoning +44 20 3513 5191. The research assistant will contact you to arrange a convenient time to attend a screening assessment in order to check you are able to participate. The screening assessment can be carried out in-person at St. George's or Springfield Hospital or remotely via MS Teams, whichever is best for you.

If you choose to attend the screening assessment, it will last between 30 minutes and 2 hours. During the appointment, you will be able to ask any questions. You will be asked to sign a consent form indicating that you agree to take part in the study. Agreement to take part will include you agreeing to therapy sessions to be video recorded if you are allocated to the group of participants attending EMDR alongside your routine neuropsychiatry appointments (please see below section on data protection and confidentiality for details).

You will then be asked to complete a questionnaire that asks you whether you have experienced any specific potentially traumatic life events in your lifetime, such as being assaulted, experiencing a life-threatening illness, being in an accident or being bullied. To take part in this study, you will need to have experienced at least 1 potentially traumatic event in your lifetime. You will not be asked to discuss the event(s) in the screening assessment, only indicate whether or not any have happened to you. Following this you will be asked to complete another questionnaire that examines any instances of dissociation you may have had over the previous 4 weeks. Dissociation is a broad term that refers to experiences of disconnection or lack of integration in your experience, such as your body feeling like it was someone else's, things around you suddenly seeming strange, or feeling like two or more people were fighting or arguing inside of yourself. There is a particular type of dissociation called identity disturbance and we will test for this as part of the assessment, as we will not be inviting anyone with this condition to take part in the research.

If the screening assessment tells us that you are eligible to take part in the study, we will ask you to complete additional questionnaires.

If you are not eligible to take part in the study and are interested in attending psychological therapy, you will be offered an appointment with Dr Cope, Principal Clinical Psychologist in the Neuropsychiatry Service, who will be able to discuss your current difficulties and psychological therapy options with you.

South West London and St George's Mental Health NHS Trust

What would taking part involve?

Completing questionnaires

Once it is confirmed you are eligible to take part in the study, you will be asked to complete additional questionnaires regarding your health-related functioning, mental health, and use of healthcare services. You will also be asked to complete questions on your demographics (e.g. relationship status, living arrangements, employment status), your medical history, current medication, and any previous psychological therapy you have attended, e.g. type, number of sessions, and focus of intervention (if known).

You will also be asked to identify the FND symptoms, e.g. seizures, tremor, limb weakness, tingling/numbness, gait disturbance (maximum two symptoms) that have the worst impact on your quality of life. You will then be given instructions regarding rating each symptom daily for 2 weeks. This can be done via an App or on paper if you prefer.

These are called "baseline measures", and you will be asked to complete questionnaires at 3 more time points: 3 months, 6 months and 9 months after your screening appointment. The research assistant will contact you at these time points to arrange completion of the questionnaires.

Randomisation (Allocation to treatment group)

Following your consent and completion of the baseline measures, you will be allocated to either EMDR plus routine appointments with your allocated neuropsychiatrist (EMDR+NPC), or routine neuropsychiatric appointments alone (NPC). You will be allocated at random by a computer programme: the research team will not be able to predict or change which group you will be assigned to. You will be told which group you have been randomly allocated to by the Trial Manager. The research assistant will not be told which group you have been allocated to, and it is important that you do not let them know which group you are in, at any point in the study. This is because if they know which group you are allocated to, it may influence how they support you in the completion of the questionnaires completed at different time points in the research.

Treatment groups

You will be invited to routine appointments with your assigned neuropsychiatrist (NPC). It is likely you will be invited to 1-3 routine neuropsychiatric appointments during the research. Each of these routine medical appointments lasts for 30 minutes. Your neuropsychiatrist may refer you to educational FND groups run in the service or for treatments for any other difficulties you may have.

If you are allocated to EMDR+NPC, you will be contacted by a psychological therapist to arrange your first EMDR appointment shortly after completing your screening. Your EMDR appointments can be held in-person at St. George's Hospital or virtually via MS Teams (your choice). You will be offered up to 16 sessions of EMDR, with each session lasting between 60 and 90 minutes (treatment duration will depend on your individual needs). The sessions will need to be completed within 6 months, so it is important that you are available and willing to meet with your therapist weekly. A minimum number of 8 attended EMDR sessions will count as "completed treatment". An optional follow-up session will be offered 1 month after treatment completion. Each of your sessions will be video recorded. This is to check the therapist is following the treatment protocol and for the therapist to review sessions in their clinical supervision.

Whether you are allocated to EMDR+NPC or NPC alone, we ask that during the study period (9 months), you do not start attending any other individual psychological therapies focused on FND or specific FND treatments, such as inpatient treatment or specialist intensive physiotherapy for FND. Your neuropsychiatrist or other doctors involved in your care can still refer you to these treatments during the trial period (if needed); we just ask you not to start any of them during the time you are part of the study if possible. If you do start attending individual psychological therapy for FND which is not part of the study, or the specific FND treatments mentioned, you will not be able to continue to take part in the study, and any data collected up until that point will be used for analysis. This is because

Cope SR, *et al. BMJ Open* 2023; 13:e073727. doi: 10.1136/bmjopen-2023-073727

we would like to compare EMDR+NPC to NPC alone, and if you receive another specific FND treatment in the same time period, we will not be able to know whether any changes are due to the treatment we are offering or the treatments you are receiving elsewhere.

Travel expenses and payment for participation

You will be reimbursed for research-related travel costs, up to the value of £20 per appointment (if you choose to attend appointments in-person, rather than virtually). Receipts/proof of travel will need to be provided.

If you are enrolled onto the trial, you will be offered a £25 incentive 9-months after consenting to participate, irrespective of whether you complete the trial or not.

Do I have to take part?

No. It is up to you to decide whether to take part. If you choose not to take part, it will not affect any routine care that you receive.

How long does the study last?

If you take part, your participation in the trial will end after 9 months, once the final assessment measures have been completed. If you take part, you will also be asked whether you would be willing to be contacted after completion of the study to potentially provide additional data for a prospective full-scale study, and/or be invited to participate in future studies.

When all participants have completed the study, some people will be invited to attend interviews regarding their experiences of the study. If you are invited to attend an interview, this will be entirely voluntary, and you will be given further information beforehand so that you can decide whether you would like to be interviewed or not.

Following your participation in the trial, your clinical care will continue to be provided by the neuropsychiatry service, if needed, and will be assessed on your individual basis.

What are the possible benefits of taking part?

We cannot guarantee any specific treatment benefits, but it is hoped that those allocated to EMDR + NPC will benefit from the therapy. Taking part in this research will contribute to the evidence-base regarding treatments for FND. Contributing to research on FND treatments has the potential to help many people with FND, as the results of this study will likely inform treatment provision for FND.

What are the possible disadvantages and risks of taking part?

Attending mental health treatments like neuropsychiatric appointments and/or psychological therapy like EMDR may result in an increase in distress and/or deterioration in physical or mental health. However, these potential changes are usually temporary. They may occur as a result of discussing difficult events, emotions and symptoms in the appointments. If you are randomly selected for EMDR+NPC, it is possible that during your EMDR sessions, focusing on a distressing memory may cause you to experience an increase in the physical sensations or emotions associated with that memory. However, this is likely to be temporary and it is hoped that any potential discomfort will be outweighed by the potential benefits of EMDR. Irrespective of which treatment group you are allocated to, if you experience an increase in distress and/or deterioration in your physical or mental health, you will be supported by the neuropsychiatry healthcare professional(s) involved in your care, and if needed, signposted or referred to relevant organisations or support resources.

South West London and St George's Mental Health NHS Trust

What if I don't want to carry on with the research project?

It is up to you to decide whether to take part or not; choosing not to take part will not disadvantage you in any way or affect any treatment you are receiving outside of the study. You are free to withdraw at any time without giving a reason. If you withdraw, it will not affect any routine care that you receive. If you withdraw early from the study, any data you provided up until that point will be used for analysis.

What if something goes wrong?

If you have a concern about any aspect of this study, you should contact the trial manager Dr Serena Vanzan or chief investigator Dr Sarah Cope [contact number 020 8725 3786, email modifi@swlstg.nhs.uk; sarah.cope@swlstg.nhs.uk]. If you are not satisfied following this, you can contact the Sponsor's Office by emailing ResearchDevelopment@swlstg.nhs.uk. If you remain unhappy and wish to complain formally, you can do this by contacting complaints@swlstg.nhs.uk. Further details can be obtained from https://www.swlstg.nhs.uk/contact-us/complaints. Or you can contact the Trust's Patient Advice and Liaison Service (PALS). Tel: 0203 513 6150 (Monday - Friday 9.30am to 4.30pm) or email pals@swlstg.nhs.uk.

In the event that something does go wrong and you are harmed during the research and this is due to someone's negligence then you may have grounds for a legal action for compensation against SWLSTG NHS Trust, but you may have to pay your legal costs. The normal National Health Service complaints mechanisms will still be available to you.

Will my taking part in this study be kept confidential?

All the information that we collect about you during the course of the research will be kept strictly confidential. The only limits to this confidentiality are that if you share information that suggests you or someone else is at risk of harm, or if you disclose a serious crime has been committed. If this happens, the researcher or clinician will need to share this information with those directly involved in your care. The researcher or clinician would try to discuss this with you before confidentiality is broken.

You will not be able to be identified in any ensuing reports or publications. All data will be pseudonymised. This means that you cannot be personally identified. Each participant will be assigned a unique screening number upon referral to the study. This number will be written on all eligibility measures and databases. A unique trial identification number will then be issued following your consent to take part. This number will be written on all clinical assessment forms/datasheets and databases used to record data on participants. All data will be kept secure at all times and maintained in accordance with General Data Protection Regulation (GDPR, 2018) requirements and archived according to clinical trial Good Clinical Practice regulations. Only researchers working with the Chief Investigator (Dr. Cope) will have access to the data. The data custodian for this project will be Dr. Cope. The data will be retained for 10 years after completion of the study.

How will we use information about you?

We will need to use information from your medical records for this research project. This information will include your:

- NHS number
- Hospital number
- Name
- Contact details
- Date of birth
- Ethnicity
- Gender
- Relationship status
- Employment status
- Presence of dependent(s)
- Presence of a carer

Cope SR, *et al. BMJ Open* 2023; 13:e073727. doi: 10.1136/bmjopen-2023-073727

BMJ Publishing Group Limited (BMJ) disclaims all liability and responsibility arising from any reliance placed on this supplemental material which has been supplied by the author(s)

South West London and St George's Mental Health NHS Trust

- Receipt of state benefits
- Current medication
- Medical history

People will use this information to do the research or to check your records to make sure that the research is being done properly. People who do not need to know who you are will not be able to see your name or contact details. Your data will have a code number instead. We will keep all information about you safe and secure.

Once we have finished the study, we will keep some of the data so we can check the results. We will write our reports in a way that no-one can work out that you took part in the study.

What are your choices about how your information is used?

You can stop being part of the study at any time, without giving a reason, but we will keep information about you that we already have.

- We need to manage your records in specific ways for the research to be reliable. This means that we won't be able to let you see or change the data we hold about you.
- If you agree to take part in this study, you will have the option to take part in future research using your data saved from this study. If you consent to this we may:
    - Use your data already collected for this study in future research.
    - Contact you regarding taking part in future research relating to this current study.

Where can you find out more about how your information is used?

You can find out more about how we use your information

- at www.hra.nhs.uk/information-about-patients/
- by contacting the trial team at modifi@swlstg.nhs.uk
- by sending an email to SWLSTG's Data Protection Officer, John Hughes, at john.hughes@swlstg.nhs.uk
- by ringing us on +44 20 3513 5191

Informing General Practitioner (GP)

If you choose to participate, your GP will be informed that you are participating in the study.

What will happen to the results of the study?

We will disseminate the results from this study in conferences, peer-reviewed journals, social media, and the website for the patient-led charity FND Hope.

We will email you a copy of the paper that presents the findings from this study once the paper has been published.

Who is organising and funding the research?

SWLSTG is the study sponsor. This project is funded by the National Institute for Health and Care Research (NIHR) under its Research for Patient Benefit (RfPB) Programme (Grant Reference Number NIHR202277).

South West London and St George's Mental Health NHS Trust

How have patients and the public been involved in this study?

The research design has been informed by a Public and Patient Involvement (PPI) meeting, where all participants had lived experience of FND. The research team has people with lived experience of FND. Two people are on the Trial Steering Committee and three other people are working with other members of the research team to make sure that the lived experience perspective is reflected in the research tools, methodology, analysis and dissemination. If the trial proves feasible, they will contribute to the design of a substantive study. The study has received guidance and support on PPI and lived experience involvement from the lived experience PPI lead for Research & Development at SWLSTG.

Who has reviewed the study?

All proposals for research using human subjects are reviewed by an Ethics Committee before they can proceed. This proposal was reviewed by West Midlands - Edgbaston Research Ethics Committee. This study has also been reviewed by the Health Research Authority.

Further information and contact details

Please contact modifi@swlstg.nhs.uk or telephone +44 20 3513 5191 if you require further information about this study.

The Chief Investigator is Dr Sarah Cope (Principal Clinical Psychologist) and her contact details are:

Neuropsychiatry Service

2nd Floor Grosvenor Wing,

St. George's Hospital,

Blackshaw Road,

SW17 0QT

(T) 020 8725 3786

(E) sarah.cope@swlstg.nhs.uk

Thank you for reading this information sheet and for your consideration regarding taking part in this research study.

South West London and St George's Mental Health NHS Trust

MODIFI: Randomised feasibility study of eye MOvement DesensItisation and reprocessing therapy (EMDR) for FunctIonal neurological disorder (FND)

Informed Consent Form

(Version 1.3, 23Nov2022)

Participant Number:______________________

Chief Investigator: Dr Sarah Cope

	Please initial box
1. I confirm that I have read the information sheet dated................... (version...........) for the above study. I have had the opportunity to consider the information, ask questions and have had these answered satisfactorily.	☐
2. I understand that my participation is voluntary and that I am free to withdraw at any time without giving any reason, without my medical care or legal rights being affected.	☐
3. I understand that relevant sections of my medical notes and data collected during the study, may be looked at by individuals from regulatory authorities or from SWLSTG NHS Trust, where it is relevant to my taking part in this research. I give permission for these individuals to have access to my records.	☐
4. I understand my personal data (such as name and contact details) will be kept for a maximum of 5 years following the end of the study and anonymised data (gathered during the trial) could be securely shared with appropriate research teams for further analysis (only with permission from SWLSTG NHS Trust).	☐
5. I understand that the information collected about me will be used to support other research in the future and may be shared anonymously with other researchers.	☐
6. I agree to my General Practitioner being informed of my participation in the study.	☐

South West London and St George's Mental Health NHS Trust

7. I understand that the information held and maintained by South West London & St. George's Mental Health NHS Trust may be used to help contact me or provide information about my health status. ☐

8. I understand that if I am allocated to EMDR plus neuropsychiatric care, my sessions of EMDR will be video-recorded for the purposes of checking the therapist is following the treatment protocol. ☐

9. I agree to be contacted for future research. ☐

10. I agree to take part in the above study. ☐

Name of Participant	Date	Signature
Name of Person taking consent	Date	Signature

Please make sure you have initialled the boxes if you agree.

When completed - 1 copy for patient, 1 original copy for Trial Master File and 1 copy for hospital records

Cope SR, *et al. BMJ Open* 2023; 13:e073727. doi: 10.1136/bmjopen-2023-073727
