# Supplementary material for: MODIFI: protocol for randomised feasibility study of eye-movement desensitisation and reprocessing therapy (EMDR) for functional neurological disorder (FND)
Source: BMJ Open. 2023 Jun 2;13(6):e073727. doi: 10.1136/bmjopen-2023-073727 (PMC10255051; doi:10.1136/bmjopen-2023-073727)
Supplement: Author's manuscript [file bmjopen-2023-073727.draft_revisions.pdf]

BMJ Open is committed to open peer review. As part of this commitment we make the peer review history of every article we publish publicly available.

When an article is published we post the peer reviewers' comments and the authors' responses online. We also post the versions of the paper that were used during peer review. These are the versions that the peer review comments apply to.

The versions of the paper that follow are the versions that were submitted during the peer review process. They are not the versions of record or the final published versions. They should not be cited or distributed as the published version of this manuscript.

BMJ Open is an open access journal and the full, final, typeset and author-corrected version of record of the manuscript is available on our site with no access controls, subscription charges or pay-per-view fees (http://bmjopen.bmj.com).

If you have any questions on BMJ Open's open peer review process please email info.bmjopen@bmj.com

MODIFI: Randomised feasibility study of eye movement desensitisation and reprocessing therapy (EMDR) for functional neurological disorder (FND)

Journal:	*BMJ Open*
Manuscript ID	bmjopen-2023-073727
Article Type:	Protocol
Date Submitted by the Author:	15-Mar-2023
Complete List of Authors:	Cope, Sarah; South West London and Saint George's Mental Health NHS Trust,<br>Smith, Jared; St George's University of London, Population Health Research Institute; South West London and Saint George's Mental Health NHS Trust, Clinical Research Unit<br>El-Leithy, Sharif; South West London and Saint George's Mental Health NHS Trust, Traumatic Stress Service<br>Vanzan, Serena; South West London and Saint George's Mental Health NHS Trust, Clinical Research Unit<br>Pentland, Caitlin; South West London and Saint George's Mental Health NHS Trust, Clinical Research Unit<br>Pick, Susannah; Institute of Psychiatry Psychology and Neuroscience, Department of Psychological Medicine<br>Golder, Dawn; FND Hope<br>Hogwood, Patricia; N/A<br>Turner, Kati; St George's University of London, PHRI<br>Billings, Jo; University College London, Department of Psychiatry<br>Edwards, Mark; King's College London, Department of Basic and Clinical Neuroscience, Institute of Psychiatry, Psychology & Neuroscience
Keywords:	NEUROLOGY, Adult psychiatry < PSYCHIATRY, Clinical trials < THERAPEUTICS

SCHOLARONE™
Manuscripts

MODIFI: Randomised feasibility study of eye movement desensitisation and reprocessing therapy (EMDR) for functional neurological disorder (FND)

By Sarah R. Cope[1*], Jared G. Smith[2,3], Sharif El-Leithy[4], Serena Vanzan[3], Caitlin Pentland[3], Susannah Pick[5], Dawn Golder[6], Patricia Hogwood, Kati Jane Turner, Jo Billings[7], & Mark J. Edwards[8]

[1]Neuropsychiatry Service, South West London & St. George's Mental Health Trust, St. George's Hospital, Blackshaw Road, SW17 0QT, UK

[2]Population Health Research Institute, St George's, University of London, Cranmer Terrace, Tooting, London, SW17 0RE, UK

[3]Clinical Research Unit, South West London & St George's Mental Health Trust, Springfield University Hospital, 61 Glenburnie Road, London, SW17 7DJ, UK

[4]Traumatic Stress Service, South West London & St. George's Mental Health NHS Trust, Springfield University Hospital, 61 Glenburnie Rd, London SW17 7DJ

[5]Department of Psychological Medicine, Institute of Psychiatry, Psychology & Neuroscience, King's College London, London, SE5 8AF

[6]FND Hope UK Registered charity in England & Wales 1173607 & Scotland SC048333

[7]Department of Psychiatry, University College London, Wing A, 6th Floor, Maple House, 149 Tottenham Court Road, London W1T 7NF, UK

[8]Department of Basic and Clinical Neuroscience, Institute of Psychiatry, Psychology & Neuroscience, King's College London, London, SE5 8AF

[*]Corresponding author: sarah.cope@swlstg.nhs.uk

Protocol: Version v3.1 23/11/2022

Word Count (excluding title page and abstract p. i-ii, Ethics Statements p. 14-15, tables & figures): 3997

Key Words: Functional neurological disorder, functional motor disorder, functional seizures, psychological therapy, eye movement desensitisation and reprocessing therapy, randomised controlled trial

For peer review only

For peer review only - http://bmjopen.bmj.com/site/about/guidelines.xhtml

Abstract

Introduction:

Functional neurological disorder (FND) refers to an involuntary loss of control over and/or aberrant perception of the body. Common presenting symptoms are functional (non-epileptic) seizures, and functional motor disorder, e.g. walking difficulties, weakness, or tremor. Greater access to effective treatments would lead to reduced distress and disability; and reduce unnecessary healthcare costs.

This study will examine eye-movement desensitisation and reprocessing therapy (EMDR) as a treatment for FND. EMDR is an evidence-based treatment for post-traumatic stress disorder (PTSD), but its use for other conditions is growing. An FND-specific EMDR protocol will be tested, and if the intervention proves feasible with promising clinical outcomes, progression to a substantive study could take place.

Methods and Analysis:

Fifty adult patients diagnosed with FND will be recruited. It will be a single-blind randomised controlled trial with two arms: EMDR (plus standard neuropsychiatric care; NPC) and standard NPC. The two groups will be compared at baseline (T0), 3 months (T1), 6 months (T2), and 9 months (T3). Measures of feasibility include safety, recruitment, retention, treatment adherence and acceptability. Clinical outcome measures will assess health-related functioning/quality of life, ratings of FND symptoms and severity, depression, anxiety, PTSD, dissociation, service-utilisation and other costs. Improvement and satisfaction ratings will also be assessed. Feasibility outcomes will be summarised using descriptive statistics. Exploratory analyses using (linear/logistic) mixed effect models will examine the rate of change in the groups' clinical outcome measures across the four time-points.

After the intervention period, a sample of participants, and clinicians, will be invited to attend semi-structured interviews. The interviews will be analysed using reflexive thematic analysis.

Ethics and Dissemination:

This study has been approved by the NHS West Midlands - Edgbaston Research Ethics Committee. Study findings will be published in open access peer-reviewed journals, presented at conferences, and communicated to participants and other relevant stakeholders.

Trial Registration:

NCT05455450 (www.clinicaltrials.gov)

Strengths and Limitations:

- This is a pragmatic randomised controlled trial embedded within an existing clinical service.
- The EMDR treatment is tailored for FND presentations.
- There has been Patient and Public Involvement (PPI) input from the design stage, and there is continued PPI involvement.
- The study will use a validated self-report measure to assess PTSD, rather than a clinical interview.

For peer review only

Introduction

Functional neurological disorder (FND) is a disorder at the interface between neurology and psychiatry. It refers to an involuntary loss of control over and/or aberrant perception of the body. Presenting symptoms can be wide-ranging with the commonest being functional (non-epileptic) seizures (FS) and functional motor disorder (mFND), e.g. walking difficulties, weakness, tremor. Risk and perpetuating factors for FND include traumatic experiences, affective disorders and experiencing chronic or acute illness. FND is one of the commonest diagnoses made in neurology, e.g. 16% of new patients in general neurology[1]; causes similar disability and impairment in quality of life as Parkinson's disease and multiple sclerosis, and high unemployment. [2] Lack of provision of assessment and treatment is associated with significant unnecessary costs, e.g. unnecessary referrals, investigations, and emergency department attendances.[3,4]

Accessing treatment is often difficult, and effective treatments for FND are still being established. Healthcare Improvement Scotland published guidance in 2012, recommending a stepped-care approach, whereby patients are assessed and diagnosed by a neurologist, and referred for relevant interventions (e.g. physiotherapy, psychology, psychiatry, occupational therapy) as required.[5] A cornerstone of FND treatment is effective communication of the diagnosis, and guidelines regarding management of FND have been published.[6,7] There have been consensus recommendations published for physiotherapy for mFND and occupational therapy.[8,9] The best evidence for mFND specifically comes from a feasibility study evaluating a specialist physiotherapy intervention compared to physiotherapy in the community, which reported positive outcomes in terms of recruitment, retention, acceptability, and clinically meaningful effect sizes.[10] A multicentre NIHR-funded RCT evaluating this is underway (International Standard Randomised Controlled Trials Number ISRCTN56136713). However, this intervention is not suitable for a large proportion of patients with mFND (only 32% of eligible patients met the inclusion criteria; most common reasons for exclusion were dominant persistent pain and psychological factors requiring treatment). There have been reports of beneficial outcomes following cognitive behavioural therapy (CBT) in uncontrolled studies for patients with mFND, but no controlled and adequately powered studies have been carried out.[11] For FS specifically, the multicentre CODES trial compared CBT plus standard medical care (SMC) to SMC alone. Although this study did not find a significant difference in monthly seizure frequency between the groups, they did find significant improvements for CBT on secondary measures (psychosocial functioning, psychological distress, and health-related quality of life).[12] Previous studies evaluating CBT for FS have reported significant improvements in seizure frequency.[13,14] More research regarding effective psychological treatments other than CBT for FND is needed, to inform delivery of treatment.

Cross-sectional studies suggest that lifetime traumatic/adverse experiences are higher in FND populations, when compared to healthy controls; in particular for those with FS, who also have higher incidences of post-traumatic stress disorder (PTSD).[15] The occurrence of severe life events immediately prior to symptom onset is significantly more frequent in those with mFND compared to psychiatric controls[16] and it has been proposed that there is a trauma subtype of FND.[17] Traumatic/adverse life events, including physical events such as injury or illness, are a risk factor and can be a trigger for developing FND. Mechanistic models of FND have been developed focusing on different levels of explanation from the neurobiological to the psychosocial. Neurobiological models have used predictive coding models of perception and movement control to suggest that symptoms in FND relate to the development of abnormal priors which are activated by misdirected attention towards the body.[18] This links closely with cognitive models suggesting that learned patterns of behaviour are triggered by abnormal threat processing.[19] Emotional dysregulation, abnormal interoceptive processing and alexithymia can all be integrated with such models, building a complex

For peer review only - http://bmjopen.bmj.com/site/about/guidelines.xhtml

For peer review only

picture of the biological and psychological processes that underpin FND.[20] This provides a scientific foundation for the development and application of specific psychological interventions to treat people with FND.

Eye-movement desensitisation and reprocessing therapy (EMDR) is an evidence-based treatment for PTSD, but its use for other conditions is growing, including treatment of somatic symptoms such as persistent pain and tinnitus.[21–24] EMDR follows a standard protocol (see Table 1).[25] Within EMDR, a target memory is brought to mind, whilst the clinician creates a distracting task that means the person's attention is divided between the memory and the present-focused task. Traditionally, eye-movements are used, but other tasks that create dual attention can be used, e.g. tapping. The working memory (WM) hypothesis suggests that focusing on the memory, whilst engaging in a competing task, results in the memory becoming less vivid and distressing. A recent systematic review, identifying 11 studies testing the WM hypothesis, concluded that bringing a distressing memory to mind, whilst engaging in a secondary task, results in reduced vividness and emotionality of the memory, and is associated with symptom reduction.[26]

Table 1: The 8 phases of EMDR according to the Standard Protocol (Shapiro 2018)

Phase	Description
**1 - 2**	Taking of patient history, assessment of suitability for EMDR, and preparation for the therapy.
**3**	Assessment of a target image whereby the patient is asked to bring a target memory to mind, identify the most upsetting image or moment, and identify the negative cognition about themselves that goes with that moment. They are also asked to identify a preferred cognition and rate their belief in that cognition. Additionally, they are asked to rate their subjective distress, identify the associated emotions, and locate where they feel the distress in their body.
**4**	Desensitisation phase: the patient is asked to bring the target memory to mind, with the negative cognition, notice where they are feeling the distress in their body, and follow the clinician's fingers with their eyes (or other alternating task that taxes working memory). After each set of eye movements, the patient is asked what they noticed, and importantly, without discussion, they are told to "go with that" alongside the eye movements. Once the distress has reduced sufficiently (this may involve multiple sessions), the clinician proceeds to the installation phase.
**5**	Installation phase: Preferred cognition is installed, again aided by eye-movements (or alternative).
**6**	Target any remaining distress in the body
**7**	Closure of the session
**8**	Assess previously targeted material and whether or not further processing is required

A systematic review of EMDR as a treatment for FND reported three case studies/series with all five cases presented having comorbid PTSD, of which four cases were successfully treated .[27–30] EMDR has been reported as a useful adjunctive therapy for two FND cases without PTSD, whereby both cases achieved resolution of FND symptoms and less distress.[31] EMDR can focus on specific past

For peer review only - http://bmjopen.bmj.com/site/about/guidelines.xhtml

adverse experiences that are contributing to pathology, memories associated with when FND symptoms began, current FND symptoms, and future predictions regarding symptoms. Theoretically, targeting distressing memories/images associated with FND could weaken cognitive representations of symptoms, and reduce threat associated with symptoms, meaning that representations of the symptoms are less easily triggered, resulting in fewer symptoms and less distress. EMDR is a therapy that can be tailored to the heterogeneous presentations of FND.

This study aims to evaluate the feasibility and acceptability of conducting a full-scale trial of EMDR for people diagnosed with FND. Feasibility will be assessed by examining recruitment rate, intervention adherence, and retention. Acceptability will be examined through attendance rates, satisfaction ratings, therapy fidelity ratings, and qualitative interviews with participants and treating therapists. Assessment of safety across the two arms will be compared. Examination of the completeness of outcome measures and variance in outcomes will be used to inform the design and power calculation of a future definitive trial.

Study Objectives:

i. Test the acceptability and feasibility of an FND-specific EMDR intervention protocol, delivered in-person or virtually. For a substantive RCT, the intervention will be subject to amendment based on the results of this trial.
ii. Investigate the value of a range of outcome measures, to determine the outcome measure with greatest effect size to enable a sample size calculation for a substantive RCT.
iii. Carry out semi-structured interviews with participants and therapists to explore experiences of EMDR and the trial; informing the intervention and design of a substantive trial.

Methods and analysis

This protocol is reported in accordance with the Standard Protocol Items: Recommendations for Intervention Trials 2013 statement.[32]

Study Design

This feasibility study is a single-blind randomised controlled trial (RCT) with two arms: EMDR (plus standard neuropsychiatric care (NPC)) and standard NPC only. The two groups will be compared at baseline (T0), 3 months (T1), 6 months (T2), and 9 months (T3). Fifty adult patients with a diagnosis of FND, confirmed by a neurologist according to standardised diagnostic criteria, will be recruited via a UK neuropsychiatry service. The research assistant (RA) and project statistician will be blind to treatment allocation. After the intervention period, semi-structured interviews will be carried with a proportion of participants and clinicians to explore their experiences and views about the trial.

Study Setting

This is a single-site study being carried out at a neuropsychiatry service, based at St. George's Hospital, Tooting, London, UK. The service is part of the South-West London and St. George's Mental Health NHS Trust (SWLSTG).

For peer review only - http://bmjopen.bmj.com/site/about/guidelines.xhtml

Public and Patient Involvement (PPI)

The research design has been informed by a PPI meeting (June 2020), where all five participants had lived experience of FND. Three PPI representatives join regular Trial Management Group (TMG) meetings, review participant literature, and will co-produce the interview schedules and contribute to the design of a substantive study. Two PPI representatives join the Trial Steering Committee (TSC).

Eligibility Criteria

Inclusion criteria:

(1) Predominant diagnosis of FS and/or mFND, with diagnosis confirmed by neurologist;

(2) Aged 18 years or over;

(3) Capacity to consent;

(4) Willingness to attend regular psychological therapy sessions;

(5) Reporting at least one traumatic event on the International Trauma Exposure Measure (ITEM).

Exclusion criteria:

(1) Non-English speaking;

(2) Current ongoing adversity that is likely to interfere with psychological therapy, e.g. domestic violence, homelessness, unresolved compensation claim/litigation;

(3) Predominant diagnosis of borderline personality disorder*;

(4) Predominant diagnosis of chronic pain condition*, e.g. fibromyalgia;

(5) Predominant diagnosis of chronic fatigue syndrome*;

(6) Diagnosis of a psychotic disorder;

(7) Diagnosis of dissociative identity disorder or score in clinical range on “identity disturbance” subscale of Multiscale Dissociation inventory;

(8) Uncontrolled epileptic seizures;

(9) Diagnosis of an eating disorder;

(10) Current severe self harm or strong suicidal ideation that requires secondary care mental health services input;

(11) Current alcohol or drug harmful use or dependence;

(12) Current diazepam use exceeding the equivalent of 10mg per day;

(13) Currently attending individual psychological therapy focused on FND or other specialist FND-specific treatment such as inpatient/outpatient multi-disciplinary treatment or intensive FND-specific physiotherapy

*Comorbid diagnosis is acceptable, as long as FND is the predominant difficulty

For peer review only

Study Interventions

EMDR plus standard neuropsychiatric care

The intervention group will be offered up to sixteen EMDR sessions, and a one-month follow-up session, as well as attending standard outpatient neuropsychiatric appointments (NPC). Participants will be given the choice of attending EMDR face-to-face or virtually via a video-consultation platform. Sessions will normally be attended weekly, with treatment completed within six months. Sessions will be 60-90 minutes long, in accordance with NICE guidance for PTSD.[33] A minimum of eight sessions was chosen based on patient feedback and previous research.[34] Optimum session duration and number will be examined as part of the trial. The optional follow-up session will occur one month after treatment completion.

EMDR for FND is a collaborative and individualised approach, following the standard EMDR protocol, but tailored for FND presentations. Treatment has three broad stages: Assessment, psychoeducation, target selection, and preparation for processing; Processing of targets; Ending of therapy. The initial sessions incorporate education regarding FND, anxiety and dissociation; formulating collaboratively with the participant regarding the development of FND symptoms; and giving a rationale for EMDR. In collaboration with the participant, target memories/images will be chosen, such as: (1) Distressing memories associated with the time when symptoms began; (2) Distressing memories from past events that may be relevant to their FND symptoms; (3) FND symptoms themselves, when present in session, or an image of them; (4) Distressing images about the future, e.g. image of having symptoms in front of others. EMDR therapy will follow the Therapy Protocol developed by the CI for this study. Table 2 shows an overview of treatment, with a guide regarding how many sessions per stage.

Table 2: Overview of EMDR for FND

Session(s)	Overview of Content
**1-3**	History taking and preparation for therapy (Phase 1-2 of standard EMDR protocol)<br>Formulating their FND presentation and increasing their understanding of FND<br>Collaboratively selecting target memories/images
**4-15***	Processing of EMDR targets using EMDR's three-pronged approach of past, present and future targets
**Final session**	End therapy
	An optional follow-up session can be offered 1 month after completing therapy (this does not count as one of the 8-16 sessions)
***Total number of sessions can be between 8-16. If completing by session 8, there will be fewer sessions in this phase of the intervention.**	

Standard Neuropsychiatric Care (NPC)

NPC is treatment-as-usual and will consist of 1-3 routine out-patient appointments with an assigned neuropsychiatrist in the trial period. Participants will not begin any FND-specific individual psychological therapy, inpatient/outpatient multi-disciplinary treatment or intensive specialist FND-

For peer review only - http://bmjopen.bmj.com/site/about/guidelines.xhtml

specific physiotherapy. If a participant begins FND-specific individual psychological or physiotherapy treatment in the trial period, they would not be able to complete the trial. Data from their last assessment point will be used for analysis. Participants can still attend psycho-educational interventions focused on FND that are administered by the service and remain part of the trial. Their assigned neuropsychiatrist can refer for psychological therapy outside of the service for any comorbid conditions, e.g. therapy for depression.

Training, supervision and fidelity checks

The study RA has received training to deliver screening interviews and collect data. The trial EMDR-trained psychological therapists have attended training on the FND-specific EMDR protocol, delivered by the Chief Investigator (SC). They receive regular clinical supervision from SC, as well as external supervision from an EMDR Consultant (a requirement of offering EMDR). Therapists will complete a session record form after every session, and these will be reviewed in supervision to enhance fidelity.

All sessions of EMDR will be video-recorded and the recordings stored on the secure SWLSTG NHS server. The therapist will share access to the recordings with SC for supervision purposes, and excerpts will be shown in EMDR supervision. Six randomly selected recordings of processing sessions will be rated for fidelity, using the EMDR Fidelity Rating Scale Version 2 – Adverse Life Experiences Processing subscale, by an EMDR Consultant.[35] These scores will be analysed to evaluate fidelity.

Primary Objectives and Outcome Measures

A mixed methods approach will be used to establish feasibility and acceptability. The feasibility criteria and progression criteria are summarised in Table 3.

Table 3: Feasibility criteria and progression criteria for MODIFI

**Criterion**	**Critical feasibility outcome**	**Other feasibility and acceptability data relevant to the criterion**	**Proposed threshold on critical outcome**
Recruitment rate	% potentially eligible participants attending screening interview	Number of potentially eligible participants identified during neuropsychiatric assessment<br><br>Number of participants who consent and are randomised<br><br>Reasons for non-eligibility	*Above 70% attending a screening interview of those approached to participate, and if attendance is less than 70%, ways to increase the screening interview attendance rate will be considered.<br><br>**If 50-70% attend a screening interview of those approached to participate, and if attendance is less than 70%, ways to increase the screening interview attendance rate will be considered.<br><br>***If below 50% attend screening interview of those approached to participate, feasibility will not be demonstrated

For peer review only - http://bmjopen.bmj.com/site/about/guidelines.xhtml

Intervention adherence	% participants randomised to EMDR+NPC who complete therapy (Completion = attendance of 8 or more sessions, maximum session number =16)	Qualitative interviews with participants who attend EMDR<br><br>Therapy session record forms<br><br>Average number of sessions attended per course of therapy	*Feasibility will be demonstrated if above 70% complete therapy (attend 8 or more EMDR sessions)<br><br>**If 50-70% participants complete therapy, ways to improve engagement will be considered<br><br>***If less than 50% participants complete therapy, feasibility will not be demonstrated.
Outcome measurements completion	% participants who complete outcome measures at all time points	Retention of participants (rates of withdrawal across both arms)<br><br>Reasons for withdrawal<br><br>Qualitative interviews with participants	*Feasibility will be demonstrated if above 70% of participants complete outcome measures at each time point.<br><br>**If 50-70% of participants complete outcome measures at all time points, ways to improve retention and completion of outcome measures will be considered<br><br>***If less than 50% of participants complete outcome measures at all time points, feasibility will not be demonstrated

* = Continue to main study without modifications; ** = Future definitive trial is feasible with modifications; *** = Future definitive trial is not feasible.

Assessment of safety (adverse/serious adverse events) will be compared between the two arms. Therapy satisfaction and therapy fidelity will also be examined. The outcome measures used will be evaluated and the primary outcome measure identified, and the required sample size for a substantive RCT will be calculated.

The nested qualitative study will explore participants' and treating therapists' experiences and views. Information from these interviews will inform design and trial materials for a substantive study, including the FND-specific EMDR protocol. As the feasibility trial includes the option of attendance of appointments virtually, take up of this option will be measured.

Outcome measures

In terms of assessing FND, there is no single validated outcome measure for FND symptoms available.[36] Ecological Momentary Assessment (EMA) using the m-Path App will be used to assess FND symptoms. Participants will rate a maximum of two symptoms, chosen at the beginning of the trial period, e.g. seizures, tremor, limb weakness, tingling/numbness, gait disturbance, for a two-week period at each time point. For each of the two symptoms chosen, they will answer five questions daily (frequency, severity, interference, associated distress, associated preoccupation), and the mean for each item will be calculated for each two-week period at each time point.

The schedule of enrolment, interventions and assessments for participants is illustrated in Table 4. For descriptions of outcome measures, please see Supplementary Material.

For peer review only - http://bmjopen.bmj.com/site/about/guidelines.xhtml

Table 4: Schedule of enrolment, interventions and assessments for participants

	Screening	Baseline (T0)	3 months (T1)	6 Months (T2)	9 months (T3)	Post-Trial period
**Enrolment**						
**Consent to contact obtained by clinical staff (neuropsychistrist)**	x					
**Contacted by RA to arrange screening interview**	x					
**International Trauma Exposure Measure (ITEM)[37]**	x					
**Multiscale Dissociation Inventory (MDI)[38]**	x		x	x	x	
**Informed consent**	x					
**Demographics recorded**	x				x*	
**History of psychological therapy**		x				
**Current medication**		x				
**Randomisation allocation**		x				
**Interventions**						
**EMDR**		●	—	—●		
**NPC**		●	—	—	—	—●
**Assessments**						
**World Health Organisation Disability Assessment Schedule (WHODAS 2.0)[39]**		x	x	x	x	
**EQ-5F-5L[40]**		x	x	x	x	
**Ecological momentary assessment of FND symptoms via m-Path App**		x	x	x	x	
**PHQ-9[41]**		x	x	x	x	
**GAD-7[42]**		x	x	x	x	
**International Trauma Questionnaire (ITQ)[43]**		x	x	x	x	
**Adult Service Use Schedule (AD-SUS)[44]**		x			x	
**Beliefs related to diagnosis and intervention**		x			x	

For peer review only - http://bmjopen.bmj.com/site/about/guidelines.xhtml

**Clinical Global Impression - Improvement Scale (CGI-I)[45] rated by participant**				x	
**CGI-I rated by participant-nominated person**				x	
**Measure of satisfaction**				x	
**Review/reporting of patient AEs/SAEs**	x	x	x	x	
**Qualitative Interviews**					
**Informed consent for qualitative interview (select participants)**					x
**Qualitative interviews with select sample of participants and trial therapists**					x

* Participants will be asked at T3 (end of trial period) whether there have been any changes regarding medication, relationship status, and employment status and any changes recorded.

Recruitment and timeline for participants

Recruitment

Potential participants will initially be screened as part of routine neuropsychiatric appointments. The psychiatrist will verbally introduce the trial to potentially eligible participants and ask for permission for the RA to contact them. Potentially eligible participants' names will be passed to the CI, who will check that they likely meet the eligibility criteria. The RA will provide potential participants with a summary of the study and will send the Participant Information Sheet (PIS) and Informed Consent Form for them to review. They will have the opportunity to ask any questions they may have. If willing to participate, the RA will obtain informed consent and arrange a screening interview.

The screening interview will be used to establish eligibility in terms of inclusion criterion (5) Reporting at least one traumatic event on the International Trauma Exposure Measure (ITEM), and exclusion criterion (7) Diagnosis of dissociative identity disorder or score in clinical range on "identity disturbance" subscale of Multiscale Dissociation inventory (MDI). [37,38] The (ITEM) will be used to assess previous adverse experiences. If they meet this inclusion criterion, potential participants will then complete the MDI, which will screen for clinical levels of dissociative "identity disturbance". Potential participants need to score in the non-clinical range of the subscale "Identity Disturbance" on the MDI to take part (score <15). Eligible participants will complete additional baseline measures, as well as completing demographic information, medical history, and listing any previous psychological therapies attended for any difficulty (not just FND).

The flow of participants is Illustrated in Figure 1.

<insert Figure 1>

Incentives

Participants will be reimbursed for research-related travel costs, up to the value of £20 per appointment (for those who choose to attend appointments in-person, rather than virtually). A non-

For peer review only - http://bmjopen.bmj.com/site/about/guidelines.xhtml

contingent £25 incentive will be offered to participants for taking part 9 months after informed consent, unrelated to whether they complete the trial or not.

Allocation and Blinding

Consenting participants will be randomised into EMDR (plus NPC) or NPC in a 1:1 ratio. A stratified block randomisation (using randomly permuted blocks of sizes 2 and 4) will be used to ensure similar numbers of patients with and without PTSD symptoms (i.e., meeting or not meeting PTSD diagnostic criteria as determined by diagnostic algorithm of the International Trauma Questionnaire (ITQ)) are (relatively) equal across arms. Randomisations will be carried out by the Trial Manager (TM) using the randomisation function on REDCap.

Blinding

The trial is a single-blind trial with the RA and statistician remaining blind to treatment allocation. It is not possible to blind participants or treating clinicians to randomisation outcome.

Analysis and Statistical Methods

Sample size

This is a feasibility trial; as such, a power calculation is neither possible nor necessary. Rather, the sample size is pragmatic. Target recruitment is 50 patients in total (25 in each arm), which is consistent with those recommended for pilot and feasibility studies to provide sufficiently reliable estimates of feasibility outcomes, e.g. recruitment, adherence and attrition rates, and adequate precision of means and variances to inform a fully powered RCT.[46–48]

Statistical Analysis Plan

Data analysis will follow a statistical analysis plan (SAP), formally agreed with the trial steering committee prior to analysis, and centred on describing key process measures to decide if a definitive trial is feasible. Participant throughput will be summarised in an extended CONSORT diagram (Eldridge et al., 2016).

Feasibility outcomes will be summarised using descriptive statistics, with 95% confidence intervals (CIs) provided to permit assumptions when planning the main trial. Data relating to (serious) adverse events, assessment, screening, and recruitment logs will be used to produce accurate estimates of safety, eligibility, recruitment and consent rates in the study population. To determine the adequacy of study inclusion and exclusion criteria, and the generalisability of the trial to the FND population, baseline sociodemographic and clinical characteristics will be compared between study participants and ineligible and non-consenting patients. Intervention adherence (e.g. EMDR session attendance) and satisfaction of care data will be used to contribute to the evaluation of the acceptability of allocated intervention/treatment arms and mean EMDR fidelity scores for rated sessions calculated to assess intervention fidelity. At each time point, retention rates will be estimated for each of the patient reported/clinical outcome measures, with consideration given to differential dropout between the arms of the trial, to identify potential (attrition) bias in treatment completion and/or data collection. EMA completion rates in each 2-week assessment period will be calculated with respect to daily assessment. All feasibility outcomes will be compared with relevant full-trial progression criteria.

For peer review only - http://bmjopen.bmj.com/site/about/guidelines.xhtml

Baseline characteristics will be reported according to treatment arm. Continuous variables will be reported as mean (standard deviation (SD)) if normally distributed or median (inter-quartile range) if non-normal, while categorical variables will be presented as frequency (%). Subsequent analyses will summarise the proposed patient-reported and clinical outcomes (e.g. quality of life and depression measures, (2-week mean) EMA symptom ratings) at each time point for each trial arm using appropriate descriptive statistics (e.g. group mean, SD). To provide an indication of potential changes in scores/frequencies between the four time points, linear/logistic mixed effects regression models will be employed performed on an intention-to-treat (ITT) basis (accounting for data assumed to be missing at random). These random intercept (mixed) models will include intervention group, time, and intervention group-by-time interaction. There will be no emphasis on hypothesis testing, however, which is reserved for the future main trial. Rather, pre-to-post-intervention standardised effect sizes (Hedges' g, relative risk) will be computed (SDs will be computed from estimated model standard errors) with associated CIs calculated to explore imprecision around effect sizes (Durlak, 2009). Due to the small sample size, important covariates (e.g. baseline score on relevant measure, gender, age) may be included in models if the two arms happen to be highly imbalanced. Additional analyses (using mixed effect models) focussed on 'per-protocol' outcomes and the potential value of the (intensively) collected EMA data on FND symptoms will also be administered (see Supplementary Material for additional detail).

A descriptive assessment of healthcare utilisation stratified by treatment arm will also be presented. The Adult Service Use Schedule (AD-SUS) will be used to record previous 6 months of health and social care resource use at baseline (T0) and 9 months of health and social care resource use at 9 months (T3). The acceptability of the AD-SUS will be assessed and key items of resource use for a future RCT will be identified. EQ-5D-5L utility scores will also be calculated. A cost effectiveness analysis will not be conducted.

Qualitative analysis plan

A sub-sample of participants in EMDR+NPC (n=8) and NPC (n=6) arms of the trial will be invited for in-depth semi-structured interviews after the intervention period. A sampling framework will be used that ensures participants are included that are representative of the sociodemographic characteristics and clinical profile (FS and FMD, presence of PTSD symptoms). Interviews will focus on the acceptability and feasibility of participating in a future larger trial of EMDR and explore experiences of recruitment practices, informed consent procedures, randomisation and range of outcomes measures. For participants in EMDR+NPC arm, interviews will also gauge the acceptability and perceived value of the EMDR intervention, suitability of number and frequency of sessions, ways of optimising engagement; and perceived benefits/limitations of the intervention as well as any recommendations for improvement. Final interview guides will be co-produced by the team, including PPI representatives.

Semi-structured interviews will also be carried out with both treating EMDR therapists. They will explore therapists' views concerning the research design and EMDR intervention protocol, including their experiences of training and delivering EMDR to patients with FND, as well as their perceptions of its deliverability within the NHS.

Interviews will be carried out by an RA, recorded and transcribed verbatim, with transcripts cross-checked against the original recordings to ensure accuracy. They will be analysed using reflexive thematic analysis.[49,50]

Trial Status

For peer review only - http://bmjopen.bmj.com/site/about/guidelines.xhtml

Enrolment of the first participant occurred on 19th December 2022. The trial is ongoing and we anticipate completing recruitment by November 2023.

Ethics and Dissemination

Research Ethical Approval

The research was reviewed by the NHS West Midlands - Edgbaston Research Ethics Committee with a favourable opinion (Reference: 22/WM/0178), and Health Research Authority approval has been received (both dated 27th September 2022).

Informed consent

Participants will provide informed consent prior to attending the screening appointment. This includes consenting to be recorded if randomised to EMDR+NPC. For the sub-sample of participants in the nested qualitative study, additional consent will be obtained at the point of being invited for interview. Please see Supplementary Material for additional detail.

Confidentiality and management of participant data

All data will be pseudo-anonymised and inputted on to the trial database, which will be saved on the secure NHS server. The Trial Master File will be backed up weekly on an encrypted hard drive. No paper copies will be stored. The data collection and management will be in line with GDPR Data Protection Act (2018). For details regarding data collection, data handling, and record keeping, refer to the Data Management Plan found in Supplementary Material.

Monitoring, Audit and Inspection

The trial will be monitored by the TMG and TSC. A Data Management Committee is not required as this is a feasibility study. The study will be self-monitored following a Monitoring Plan protocol. Please see Supplementary Material for detailed information.

Access to the final trial dataset

The CI and statistician will have access to the final trial dataset. If anyone else requires access, a request will need to be made via the TSC.

Post trial care

Participants who take part in the trial will have access to support from the Neuropsychiatry Service. Their care will be overseen by their allocated neuropsychiatrist and appropriate referrals made, if needed.

Dissemination

Trial findings will be published in a peer-reviewed journal or platform within 24 months from study completion. Authorship will be determined in accordance with the ICMJE guidelines and other

For peer review only - http://bmjopen.bmj.com/site/about/guidelines.xhtml

contributors will be acknowledged. Participants will be notified via email after the results have been published. Trial Registries will be updated during the study and the trial protocol and key outcomes will be made publicly available within 12 months of study completion.

For peer review only

For peer review only - http://bmjopen.bmj.com/site/about/guidelines.xhtml

Contributors:

SC, MJE, JGS, SEL & SP provided significant input into the conception and design of the study. JGS completed the statistical analysis plan. JGS and SC drafted the qualitative analysis plan, and JB will supervise the qualitative aspects of the study. DG, PH, and KJT provided expert by experience input. SC trained the research assistant and trial therapists in reference to the trial protocols. SV and CP inputted into protocol modifications during the study set up phase. SC wrote the first draft of the manuscript. All authors reviewed the manuscript, and gave the final approval of the version to be published.

Sponsor:

South West London & St. George's Mental Health NHS Trust (SWLSTG)

R&D Office

Springfield University Hospital

1st Floor, Newton Building 7

61 Glenburnie Road, London SW17 7DJ

E: ResearchDevelopment@swlstg.nhs.uk

Competing interests:

SC - None declared; JS - None declared; SEL- None declared; SV – None declared; CP – None declared; SP – None declared; DG – None declared; PH – None declared; KT – None declared; JB - None declared; MJE – None declared

Funding:

This project is funded by the National Institute for Health and Care Research (NIHR) under its Research for Patient Benefit (RfPB) Programme (Grant Reference Number 202277). The views expressed are those of the author(s) and not necessarily those of the NIHR or the Department of Health and Social Care.

Sponsor roles and responsibilities

The Sponsor is responsible for providing the research team with adequate arrangements to successfully complete the trial, and to monitor its compliance with ethical guidelines and legislation. It is not involved in any aspect of the study design and management, or in its data collection, analysis or interpretation. SWLSTG Mental Health NHS Trust is the data controller for the study and all data shall return to the Sponsor at the end of the trial.

Funder roles and responsibilities

NIHR is not involved in any aspect of the study design and management, data collection, analysis, or interpretation. Core publications (Protocol and Results paper) were agreed with the NIHR before the start of the trial. NIHR will be acknowledged in any public dissemination of the research, including peer-reviewed articles, conference presentations and patient involvement activities.

For peer review only - http://bmjopen.bmj.com/site/about/guidelines.xhtml

For peer review only

Data availability statement:

Pseudoanonymised participant data is available upon reasonable request from the corresponding author Dr Sarah Cope (sarah.cope@swlstg.nhs.uk). Conditions for reuse will need to be discussed with the corresponding author on a case-by-case basis

Research Ethical Approval

The research was reviewed by the NHS West Midlands - Edgbaston Research Ethics Committee with a favourable opinion (Reference: 22/WM/0178), and Health Research Authority approval has been received (both dated 27th September 2022).

Acknowledgements:

The authors would like to thank the Trial Steering Committee (Dr Timothy Nicholson, Professor Richard J. Brown, Steve Portelly, Kirsty Griffin); staff working in the Neuropsychiatry Service (Dr Niruj Agrawal, Dr Norman Poole, Dr Andrea de Angelis, Dr Jacob Bird, Dr Gertrude Okello, Dr Charlotte Wattebot O'Brien, Dr Hendrik Hinrichsen, Dr Barnaby Proctor, Carla Vianello, Holly Zephrine, Rebecca Ewens, Cheryl Hamilton, Afsana Zaman) and St. George's Hospital (Dr Glenn Nielsen, Dr Shai Betteridge, Dr Jan Coebergh); and Maeve Crowley for her expert EMDR supervision.

For peer review only

For peer review only - http://bmjopen.bmj.com/site/about/guidelines.xhtml

References

1. Stone J, Carson A, Duncan R, et al. Who is referred to neurology clinics? - The diagnoses made in 3781 new patients. *Clin Neurol Neurosurg*. 2010;112(9):747-751. doi:10.1016/j.clineuro.2010.05.011

2. Carson A, Stone J, Hibberd C, et al. Disability, distress and unemployment in neurology outpatients with symptoms “unexplained by organic disease.” *J Neurol Neurosurg Psychiatry*. 2011;82(7):810-813. doi:10.1136/jnnp.2010.220640

3. Magee JA, Burke T, Delanty N, Pender N, Fortune GM. The economic cost of nonepileptic attack disorder in Ireland. *Epilepsy & Behavior*. 2014;33:45-48.

4. Crimlisk HL, Bhatia KP, Cope H, David AS, Marsden D, Ron MA. Patterns of referral in patients with medically unexplained motor symptoms. *J Psychosom Res*. 2000;49(3):217-219. doi:10.1016/s0022-3999(00)00167-7

5. Healthcare Improvement Scotland. Stepped care for functional neurological symptoms. *Health Improvement Scotland*. 2012;(February). http://www.healthcareimprovementscotland.org/our_work/long_term_conditions/neurological_health_services/neurological_symptoms_report.aspx

6. Gilmour GS, Nielsen G, Teodoro T, et al. Management of functional neurological disorder. *J Neurol*. 2020;267(7):2164-2172. doi:10.1007/s00415-020-09772-w

7. Stone J, Carson A, Hallett M. Explanation as treatment for functional neurologic disorders. *Handb Clin Neurol*. 2016;139:543-553. doi:10.1016/B978-0-12-801772-2.00044-8

8. Nicholson C, Edwards MJ, Carson AJ, et al. Occupational therapy consensus recommendations for functional neurological disorder. *J Neurol Neurosurg Psychiatry*. 2020;91(10):1037-1045. doi:10.1136/jnnp-2019-322281

9. Nielsen G, Stone J, Matthews A, et al. Physiotherapy for functional motor disorders: A consensus recommendation. *J Neurol Neurosurg Psychiatry*. 2015;86(10):1113-1119. doi:10.1136/jnnp-2014-309255

10. Nielsen G, Buszewicz M, Stevenson F, et al. Randomised feasibility study of physiotherapy for patients with functional motor symptoms. *J Neurol Neurosurg Psychiatry*. 2017;88(6):484-490. doi:10.1136/jnnp-2016-314408

11. O’Connell N, Watson G, Grey C, Pastena R, McKeown K, David AS. Outpatient CBT for Motor Functional Neurological Disorder and Other Neuropsychiatric Conditions: A Retrospective Case Comparison. *J Neuropsychiatry Clin Neurosci*. 2020;32(1):58-66. doi:10.1176/appi.neuropsych.19030067

12. Goldstein LH, Robinson EJ, Mellers JDC, et al. Cognitive behavioural therapy for adults with dissociative seizures (CODES): a pragmatic, multicentre, randomised controlled trial. *Lancet Psychiatry*. 2020;7(6):491-505. doi:10.1016/S2215-0366(20)30128-0

13. Goldstein LH, Chalder T, Chigwedere C, et al. Cognitive-behavioral therapy for psychogenic nonepileptic seizures: A pilot RCT. *Neurology*. 2010;74(24):1986-1994. doi:10.1212/WNL.0b013e3181e39658

14. LaFrance WC, Baird GL, Barry JJ, et al. Multicenter pilot treatment trial for psychogenic nonepileptic seizures: A randomized clinical trial. *JAMA Psychiatry*. 2014;71(9):997-1005. doi:10.1001/jamapsychiatry.2014.817

For peer review only - http://bmjopen.bmj.com/site/about/guidelines.xhtml

15. Ludwig L, Pasman JA, Nicholson T, et al. Stressful life events and maltreatment in conversion (functional neurological) disorder: systematic review and meta-analysis of case-control studies. *Lancet Psychiatry*. 2018;5(4):307-320. doi:10.1016/S2215-0366(18)30051-8

16. Nicholson TR, Aybek S, Craig T, et al. Life events and escape in conversion disorder. *Psychol Med*. 2016;46(12):2617-2626. doi:10.1017/S0033291716000714

17. Paredes-Echeverri S, Guthrie AJ, Perez DL. Toward a possible trauma subtype of functional neurological disorder: Impact on symptom severity and physical health. *Front Psychiatry*. 2022;13. doi:10.3389/fpsyt.2022.1040911

18. Edwards MJ, Bhatia KP. Functional (psychogenic) movement disorders: Merging mind and brain. *Lancet Neurol*. 2012;11(3):250-260. doi:10.1016/S1474-4422(11)70310-6

19. Brown RJ, Reuber M. Towards an integrative theory of psychogenic non-epileptic seizures (PNES). *Clin Psychol Rev*. 2016;47:55-70. doi:10.1016/j.cpr.2016.06.003

20. Pick S, Goldstein LH, Perez DL, Nicholson TR. Emotional processing in functional neurological disorder: a review, biopsychosocial model and research agenda. *J Neurol Neurosurg Psychiatry*. 2019;90(6):704-711. doi:10.1136/jnnp-2018-319201

21. Lewis C, Roberts NP, Andrew M, Starling E, Bisson JI. Psychological therapies for post-traumatic stress disorder in adults: systematic review and meta-analysis. *Eur J Psychotraumatol*. 2020;11(1). doi:10.1080/20008198.2020.1729633

22. Shapiro F. The Role of Eye Movement Desensitization and Reprocessing (EMDR) Therapy in Medicine : Addressing the Psychological and Physical Symptoms Stemming from Adverse Life Experiences. 2014;18(1):71-77.

23. Tesarz J, Leisner S, Gerhardt A, Janke S, Seidler GH, Eich W. NEUROSCIENCE SECTION Review Article Effects of Eye Movement Desensitization and Reprocessing (EMDR) Treatment in Chronic Pain Patients : A Systematic Review. Published online 2014:247-263.

24. Rikkert M, van Rood Y, de Roos C, Ratter J, van den Hout M. A trauma-focused approach for patients with tinnitus: the effectiveness of eye movement desensitization and reprocessing–a multicentre pilot trial. *Eur J Psychotraumatol*. 2018;9(1). doi:10.1080/20008198.2018.1512248

25. Shapiro F. *Eye Movement Desensitization and Reprocessing (EMDR) Therapy: Basic Principles, Protocols, and Procedures*. Third Edition. The Guildford Press; 2018.

26. Wadji DL, Martin-Soelch C, Camos V. Can working memory account for EMDR efficacy in PTSD? *BMC Psychol*. 2022;10(1):245. doi:10.1186/s40359-022-00951-0

27. Chemali Z, Meadows ME. The use of eye movement desensitization and reprocessing in the treatment of psychogenic seizures. *Epilepsy and Behavior*. 2004;5(5):784-787. doi:10.1016/j.yebeh.2004.06.003

28. Kelley SDM, Benbadis S. Eye movement desensitization and reprocessing in the psychological treatment of trauma-based psychogenic non-epileptic seizures. *Clin Psychol Psychother*. 2007;14(2):135-144. doi:10.1002/cpp.525

29. Silver SM, Rogers S, Russell M. Eye movement desensitization and reprocessing (EMDR) in the treatment of war veterans. *J Clin Psychol*. 2008;64(8):947-957. doi:10.1002/jclp.20510

30. Cope S, Mountford L, Smith J, Agrawal N. Eye Movement Desensitisation and Reprocessing Therapy (EMDR) to treat functional neurological disorder: A review. *Journal of EMDR Practice and Research*. Published online 2018.

For peer review only - http://bmjopen.bmj.com/site/about/guidelines.xhtml

31. Cope SR. EMDR as an Adjunctive Psychological Therapy for Patients With Functional Neurological Disorder: Illustrative Case Examples. *Journal of EMDR Practice and Research*. Published online 2020:EMDR-D-20-00008. doi:10.1891/emdr-d-20-00008

32. Chan AW, Tetzlaff JM, Altman DG, et al. SPIRIT 2013 Statement: Defining Standard Protocol Items for Clinical Trials. *Ann Intern Med*. 2013;158(3):200. doi:10.7326/0003-4819-158-3-201302050-00583

33. National Institute for Health and Care Excellence. *Post-Traumatic Stress Disorder (NICE Guideline 116)*.; 2018.

34. Varese F, Sellwood W, Aseem S, et al. Eye movement desensitization and reprocessing therapy for psychosis (EMDRp): Protocol of a feasibility randomized controlled trial with early intervention service users. *Early Interv Psychiatry*. 2021;15(5):1224-1233. doi:10.1111/eip.13071

35. Korn DL, Maxfield L, Nancy Smyth CJ, Stickgold R. EMDR Fidelity Rating Scale (EFRS) The Manual. *EFRS Manual Version*. 1:2017-2025. http://emdrresearchfoundation.org/emdr-fidelity-

36. Pick S, Anderson D, Asadi-pooya AA, et al. Outcome measurement in functional neurological disorder: a systematic review and recommendations. *J Neurol Neurosurg Psychiatry*.

37. Hyland P, Karatzias T, Shevlin M, et al. Does requiring trauma exposure affect rates of ICD-11 PTSD and complex PTSD? Implications for DSM–5. *Psychol Trauma*. 2021;13(2):133-141. doi:10.1037/tra0000908

38. Brière J. MDI, Multiscale dissociation inventory: Professional manual. Published online 2002. Accessed August 31, 2018. https://scholar.google.co.uk/scholar?hl=en&as_sdt=0%2C5&q=briere+2002+MDI&oq=briere+2002+

39. World Health Organisation (WHO). *Measuring Health and Disability: Manual for WHO Disability Assessment Schedule (WHODAS 2.0)*. (Ustun T, Kostanjsek S, Chatterji JR, eds.).; 2010.

40. Herdman M, Gudex C, Lloyd A, et al. Development and preliminary testing of the new five-level version of EQ-5D (EQ-5D-5L). *Quality of Life Research*. 2011;20(10):1727-1736. doi:10.1007/s11136-011-9903-x

41. Kroenke K, Spitzer RL. The PHQ-9: A New Depression Diagnostic and Severity Measure. *Psychiatr Ann*. 2002;32(9):509-515. doi:10.3928/0048-5713-20020901-06

42. Spitzer RL, Kroenke K, Williams JBW, Löwe B. A Brief Measure for Assessing Generalized Anxiety Disorder. *Arch Intern Med*. 2006;166(10):1092. doi:10.1001/archinte.166.10.1092

43. Cloitre M, Shevlin M, Brewin CR, et al. The International Trauma Questionnaire: development of a self-report measure of ICD-11 PTSD and complex PTSD. *Acta Psychiatr Scand*. 2018;138(6):536-546. doi:10.1111/acps.12956

44. Tyrer P, Cooper S, Salkovskis P, et al. Clinical and cost-effectiveness of cognitive behaviour therapy for health anxiety in medical patients: a multicentre randomised controlled trial. *Lancet*. 2014;383(9913):219-225. doi:10.1016/S0140-6736(13)61905-4

45. Guy W. Clinical Global Impressions (CGI) Scale, Modified. In: Rush JA, ed. *Handbook of Psychiatric Measures*. 1st Edition. American Psychiatric Association; 2000.

46. Sim J, Lewis M. The size of a pilot study for a clinical trial should be calculated in relation to considerations of precision and efficiency. *J Clin Epidemiol*. 2012;65(3):301-308. doi:10.1016/j.jclinepi.2011.07.011

For peer review only - http://bmjopen.bmj.com/site/about/guidelines.xhtml

47. Lancaster GA, Dodd S, Williamson PR. Design and analysis of pilot studies: recommendations for good practice. *J Eval Clin Pract*. 2004;10(2):307-312. doi:10.1111/j..2002.384.doc.x

48. Julious SA. Sample size of 12 per group rule of thumb for a pilot study. *Pharm Stat*. 2005;4(4):287-291. doi:10.1002/pst.185

49. Braun V, Clarke V. Using thematic analysis in psychology. *Qual Res Psychol*. 2006;3(2):77-101. doi:10.1191/1478088706qp063oa

50. Braun V, Clarke V. Reflecting on reflexive thematic analysis. *Qual Res Sport Exerc Health*. 2019;11(4):589-597. doi:10.1080/2159676X.2019.1628806

Figure 1: CONSORT flow diagram for MODIFI study

For peer review only

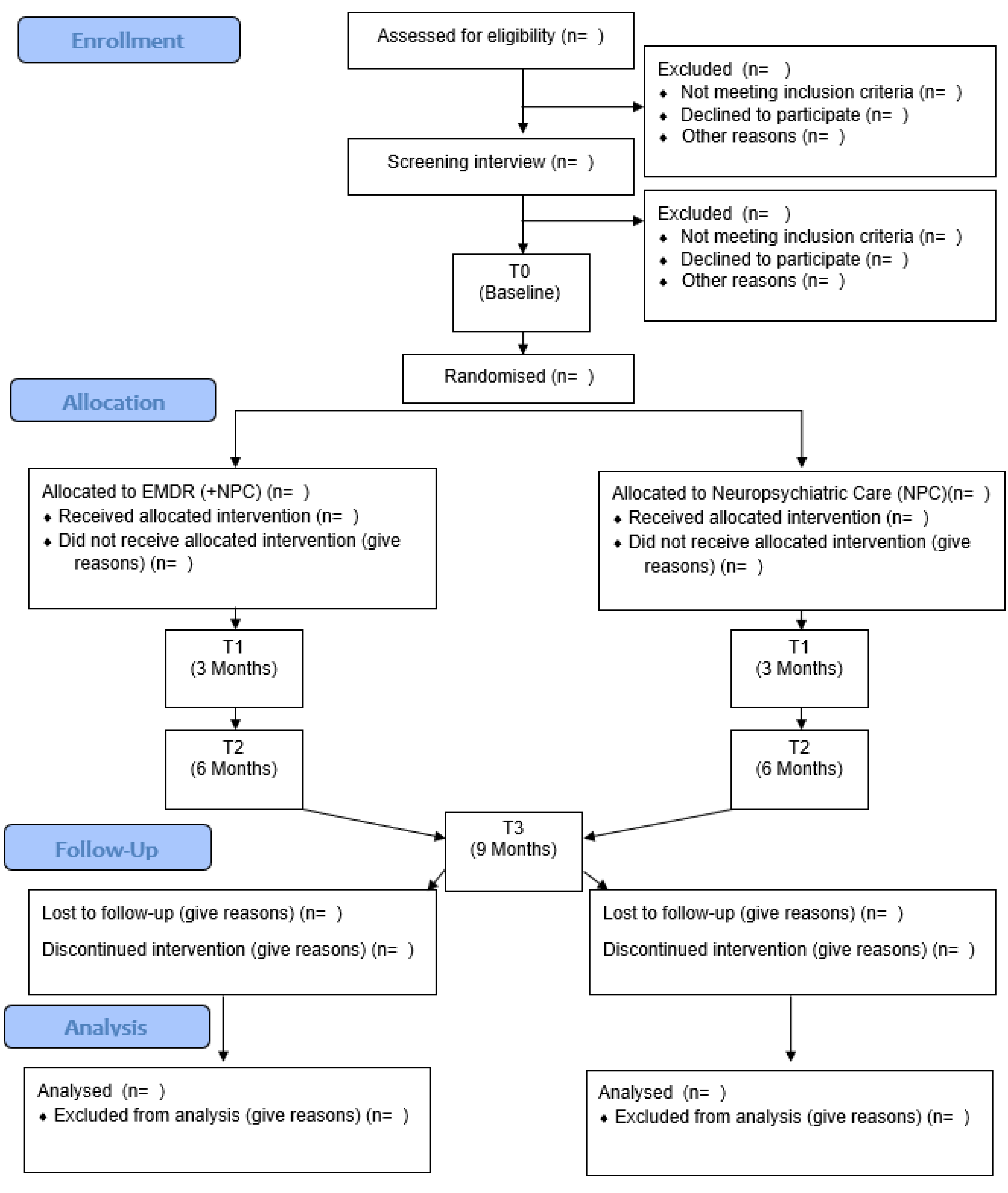
Enrollment
Assessed for eligibility (n=)
Excluded (n=)
Not meeting inclusion criteria (n=)
Declined to participate (n=)
Other reasons (n=)
Screening interview (n=)
Excluded (n=)
Not meeting inclusion criteria (n=)
Declined to participate (n=)
Other reasons (n=)
T0
(Baseline)
Randomised (n=)
Allocation
Allocated to EMDR (+NPC) (n=)
Received allocated intervention (n=)
Did not receive allocated intervention (give reasons) (n=)
Allocated to Neuropsychiatric Care (NPC)(n=)
Received allocated intervention (n=)
Did not receive allocated intervention (give reasons) (n=)
T1
(3 Months)
T1
(3 Months)
T2
(6 Months)
T2
(6 Months)
T3
(9 Months)
Follow-Up
Lost to follow-up (give reasons) (n=)
Discontinued intervention (give reasons) (n=)
Lost to follow-up (give reasons) (n=)
Discontinued intervention (give reasons) (n=)
Analysis
Analysed (n=)
Excluded from analysis (give reasons) (n=)
Analysed (n=)
Excluded from analysis (give reasons) (n=)

MODIFI Protocol – Supplementary Material

Contents

Outcome Measures 2
Additional details regarding Consent, Blinding, and Confidentiality 5
Additional details regarding Analysis and Statistical Methods 8
Recording and Reporting of Events 10
Monitoring, Audit & Inspection 12
Data Management Plan (DMP) 15
Participant Information Sheet 21

For peer review only

Outcome Measures

I. Health-Related Quality of Life/Functioning:

World Health Organisation Disability Assessment Schedule (WHODAS 2.0) (WHO, 2010) is a 36-item generic assessment instrument for health and disability. The questionnaire asks about difficulties due to health conditions and asks participants to rate these on a scale with 5 levels: None, Mild, Moderate, Severe, Extreme or cannot do.

EQ-5D-5L (Herdman et al., 2011) consists of a descriptive system assessing five dimensions: mobility, self-care, usual activities, pain/discomfort and anxiety/depression. Each dimension has 5 levels: no problems, slight problems, moderate problems, severe problems and extreme problems. It also has a self-rated health (0-100) visual analogue scale, where the endpoints are labelled 'The worst health you can imagine' (0) and 'The best health you can imagine' (100).

II. FND:

There is no single validated outcome measure for FND symptoms available (Pick et al., 2020). Ecological Momentary Assessment (EMA) using the m-Path App, which is well supported and maintained across all smartphone platforms, will be used to assess FND symptoms.

Participants will rate a maximum of 2 symptoms, chosen at the beginning of the trial period, e.g. seizures, tremor, limb weakness, tingling/numbness, and gait disturbance. A paper version will be available to participants if necessary. Participants will be encouraged to choose the 1 or 2 symptom(s) that currently have the worst impact on their quality of life.

Each participant would answer the following questions once a day over each 2-week time period for a maximum of two symptoms:

Select the symptom you are referring to [seizures, tremor, limb weakness, tingling/numbness, jerks, gait disturbance]. With this symptom in mind, please rate the following:

a. If the symptom is episodic (not present all the time), rate the number of times (frequency) it has occurred in the previous 24 hours, or answer not applicable (n/a) if your symptom is continuous

b. Over the last 24 hours, please rate the severity of the symptom, from 0 (none) – 10 (extremely severe). If the symptom has not occurred, rate 0 for this question.

c. Over the last 24 hours, please rate the extent the symptom has interfered with your daily functioning, from 0 (not at all) – 10 (extreme interference)

d. Over the last 24 hours, please rate the extent the symptom has distressed you, from scale 0 (none) – 10 (extreme distress)

e. Over the last 24 hours, please rate the extent the symptom has preoccupied you (been on your mind), from 0 (not at all) – 10 (all of the time)

For each of the 2 symptoms chosen, the mean for each item will be calculated for each 2-week period at each time point.

For peer review only - http://bmjopen.bmj.com/site/about/guidelines.xhtml

III. Depression and anxiety:

PHQ-9 (Kroenke & Spitzer, 2002) is a 9-item measure assessing presence and severity of depression. Each item is rated on a 4-point scale ranging from 'Not at all' to 'Nearly every day'.

GAD-7 (Spitzer et al., 2006) is a 7-item measure assessing presence and severity of anxiety. Each item is rated on a 4-point scale ranging from 'Not at all' to 'Nearly every day'.

IV. PTSD:

International Trauma Exposure Measure (ITEM) (Hyland et al., 2021) is a checklist developed to capture traumatic life events, and their associated features, in a manner consistent with the description of a trauma in the ICD-11 diagnostic manual. It assesses exposure to different traumatic life events across different developmental periods (childhood, adolescence, adulthood, and lifetime); frequency of exposure to one's most distressing traumatic event; and the main emotion associated with one's most distressing traumatic event.

International Trauma Questionnaire (ITQ) (Cloitre et al., 2018) is a valid and reliable measure that assesses PTSD and Complex PTSD in line with the ICD-11.

V. Dissociation:

Multiscale Dissociation Inventory (MDI) (Briere, 2002) is a 30-item self-report measure of dissociative experiences in the previous month. It measures six different types of dissociation: disengagement, depersonalisation, derealisation, emotional constriction, memory disturbance, and identity dissociation.

VI. Service utilisation and other cost variables:

Adult Service Use Schedule (AD-SUS) (Tyrer et al., 2014). The AD-SUS was developed by an economist for previous work in similar populations and was adapted for the needs of this evaluation. It assesses employment, healthcare utilisation and medication.

VIII. Improvement:

Clinical Global Impression – Improvement Scale (CGI-I) rated by participant (single item, 7-point scale) (Guy, 2000)

CGI-I rated by person nominated by participant (e.g. family member, partner, close friend, carer) (single item, 7-point scale)

IX. Beliefs about diagnosis and intervention:

Agreement with diagnosis of FND (single item, 11-point scale)

For peer review only - http://bmjopen.bmj.com/site/about/guidelines.xhtml

Preference regarding treatment (EMDR + NPC, NPC or no preference)

Belief in having been given the right treatment (single item, 11-point scale)

X. Measure of satisfaction

Satisfaction rating of treatment (single-item, 11-point scale)

For peer review only

Additional details regarding Consent, Blinding, and Confidentiality

The RA will assess risk and capacity to provide consent throughout the screening and assessment procedures, with input from the Chief Investigator, who is a clinical psychologist. The limits of confidentiality will be made clear to participants. There will be a standard protocol for assessing and managing safeguarding and/or risk disclosures, which will follow safeguarding relevant policies and procedures of SWLSTG Mental Health NHS Trust.

Those not eligible to take part in the research due to not endorsing experiencing any adverse events or experiencing high identity disturbance, and who are interested in the option of attending psychological therapy, will be offered an appointment with the CI (who is also the team's lead clinical psychologist) to discuss treatment options local to them or within the service. This will mean if non-eligible participants are distressed by non-inclusion, the RA will be able to offer them an appointment with a clinical member of staff.

Anonymised information on participants who are not randomised will include demographic data, diagnoses, type and duration of FND symptoms, the reason not eligible for trial participation, or if they are eligible but declined.

Consent

Participants will consent by one of the following:

Face to face with the RA. The participant and RA will sign the consent form. The RA will scan the original and save a copy in the electronic site file, upload a copy to the patient's electronic notes, and give a copy of the form to the participant. The paper form will be shredded.

Remote consent, whereby the consent form is shared electronically and the participant signs electronically and returns the form to the RA, who will then sign the form themselves and send a fully signed copy to the participant. The RA will file the original in the Trial Master File (TMF) and upload a copy to the patient's electronic notes.

The RA who will be responsible for taking written informed consent will have received training in Good Clinical Practice (GCP) alongside internal additional training by the study team.

Additional consent provisions for collection and use of participant data

All trial participants will be asked whether they would be willing to be contacted at a later stage for participating in further studies related to the trial. This consent to contact will include the qualitative interview. It will be made clear that this is optional and that declining consent to be contacted for participation in additional studies will not prevent them from taking part in the trial.

For the qualitative interview, it will also be made clear that not everyone will be contacted, since we will only be recruiting a sub-sample. If a participant indicates willingness, the RA will contact them directly once all participants have completed the study period, and all

For peer review only

quantitative data has been collected. Written consent will be obtained from those consenting to interview, and the form will be stored as above.

EMDR Therapists will also provide written informed consent to participate in interviews. The signed form will be filed in the site file, and a copy of the form will be given to the therapist.

Additional details regarding blinding

Participants will be reminded, when the RA meets with them to complete measures, that the RA must not be told which arm they have been randomised to. The TM will input which arm (Group 1 or Group 2) each participant was allocated to after all participants have completed their time in the study and the data has been inputted, but they will not disclose whether Group 1/2 refers to EMDR+NPC or NPC, so that the statistician will remain blind to treatment allocation whilst carrying out data analysis.

Long-term follow-up

As part of informed consent, participants will be asked to consent to open-label follow-up to potentially provide additional data for a prospective substantive RCT.

Withdrawal criteria

It will be made clear to participants that they have the right to withdraw from the study at any time for any reason, without the need to justify their decision, and that it will not affect their routine care. The investigator also has the right to withdraw participants from the study in the event of clinical contra-indications. Should a participant withdraw from therapy only but not from the study, efforts will be made to continue to obtain follow-up data, with the permission of the participant. Should a participant withdraw from the study, we will still use any previous data collected from that participant up to the point of withdrawal, but will make no further attempt to contact or collect data

Data protection and patient confidentiality

Clinical confidentiality

During the screening interview, potential participants will be advised of the limits of confidentiality (i.e. that the researcher will have a duty to inform health professionals if the participant discloses information any safeguarding or risk issues). It is also possible that disclosure of significant criminal acts potentially requiring action will occur during assessment and therapy sessions. The research team will follow the protocol for Assessing and Managing Safeguarding and Risk Disclosures. The RA and trial therapists will seek supervision and ensure appropriate action is taken as soon as possible. The limits of confidentiality and possibility of action arising from certain disclosures will be noted in the Participant Information Sheet (PIS). Potential participants will be offered at least 24 hours to consider all the information provided before written consent is obtained. Therapists will discuss the limits of confidentiality with participants allocated to the EMDR+NPC at the start of therapy, and at any appropriate subsequent points during the therapy.

For peer review only - http://bmjopen.bmj.com/site/about/guidelines.xhtml

Data confidentiality

Research data will be confidential unless a participant discloses information that indicates that they or another person are at risk of harm (see clinical confidentiality section above). All participants will be informed of this during the written informed consent process.

All data will be pseudonymised. Each participant will be assigned a unique participant number when they are referred to the trial, which will remain the same for the duration of their involvement in the trial. This number will be recorded on all eligibility measures, forms and databases used to record data on participants. An electronic record sheet linking participant identity, contact details, and trial identification number will be created and saved on the secure Trust drive. The password will only be shared with the research team. All data will be kept secure at all times and maintained in accordance with General Data Protection Regulation (GDPR, 2018) requirements and archived according to clinical trial GCP regulations. Participant consent forms will be retained electronically, kept confidential and stored securely.

Only the RA, TM and chief investigator will have access to the trial database. The statistician will receive a copy of the database once data collection is complete and the database is locked.

Therapy notes will be recorded on Rio, the electronic clinical record system used by SWLSTG, in line with Trust procedures. The RA will access the system when a participant referral is received, but will not need to access the system thereafter, so will not view participants' clinical records after recruitment to the study.

Recordings

All therapy sessions (with participant consent) will be recorded to monitor the fidelity of the intervention delivery. When sessions are held online via MS Teams, recording will take place via the platform. When sessions are held in-person, clinicians will record each session by using MS Teams too (they will position their laptop so both participant and clinician can be viewed). Each recording file will be named with participant identifier and date, and will be stored on the secure SWLSTG NHS server. Recordings of the therapy will be accessible to the participant's therapist, the CI, and excerpts will be shown in clinical supervision. A random selection of processing sessions will be shown to an EMDR Consultant in order to rate the sessions for fidelity. The video recordings are only being used to enhance fidelity to the therapy protocol, and they will be deleted after a random selection of them has been rated for fidelity.

Interviews will be held remotely via MS Teams or in-person. Recordings will take place via MS Teams in both instances with the video recording function turned off (only audio will be captured). All recordings will be labelled and stored securely on the Trust server. All audio recordings will be deleted after transcription has been completed. The transcriptions will be stored in the TMF, and stored for a minimum of 10 years in line with the other essential data.

For peer review only - http://bmjopen.bmj.com/site/about/guidelines.xhtml

Additional details regarding Analysis and Statistical Methods

Per-protocol analyses of outcome measures

Exploratory analyses using mixed effect models will examine the rate of change in intervention and control groups on outcome measures across four time-points, adjusting for relevant baseline scores and variables of interest (predominant symptom, presence of PTSD, levels of dissociation), and investigate changes on a per-protocol basis (focused on intervention adherence; i.e. including only participants who attended at least 8 sessions and with post-treatment data).

EMA data

The potential value of the (intensively) collected EMA data on FND symptoms will also be considered. EMA completion rates in each 2-week assessment period will be calculated with respect to daily assessment (total days with a completed assessment divided by total number of scheduled assessments); rates will be calculated for all assessments combined and for each assessment separately (symptom frequency, severity, and symptom-associated interference, preoccupation and distress) and the proportion of participants completing at least 33% of the total number of EMAs will be recorded.

Within participant day-to-day variability of repeated FND symptom ratings (e.g., frequency, severity) and Cronbach's alphas for EMA items (symptom severity, interference, distress, preoccupation) will be calculated at each time point to explore reliability and internal consistency of the EMA App, while exploratory mixed effect modelling analyses will adopt a three-level structure (repeated measurements at each EMA in addition to time point and participant). To explore relationships of EMA App data with other clinical measures, bivariate correlations (Pearson r or Spearman rho, according to data distribution) will be completed between 2-week mean EMA symptom ratings and corresponding WHODAS scores and between change in 2-week mean EMA symptom ratings and patient and clinician-rated CGI-I scales/change in WHODAS across corresponding time points. These analyses are expected to inform the analytic plan for a future larger study.

Procedure(s) to account for missing or spurious data

All available data will be included in the data listings and tabulations. If data exhibit missing or spurious values, aspects related to their collection, recording, and analysis will be investigated. The frequency (percentage) of participants with missing outcome values at each time point (retention rates), in addition to the reasons for missingness (where available), will be summarised. For (continuous/categorical) secondary outcomes, intention to treat (ITT) (linear/logistic) mixed effect models with maximum likelihood estimation will examine the rate of change in intervention and control groups on outcome measures across three post-treatment time-points, accounting for data missing at random. Given the feasibility status of the study, a full sensitivity analysis testing various missing data assumptions is not viable. Nevertheless, we will administer additional analyses imputing values for missing data using a conservative last observation carried forward (LOCF) procedure (in these analyses, where data is missing from assessment, a first observation carried backwards (FOCB) procedure will also be adopted). Safety and adverse/unwanted effects will be reported on an

For peer review only - http://bmjopen.bmj.com/site/about/guidelines.xhtml

as-treated basis (as opposed to an ITT approach), as these are best considered using the most accurate information.

For peer review only

For peer review only - http://bmjopen.bmj.com/site/about/guidelines.xhtml

Recording and Reporting of Events

The recording and reporting of AEs will follow the "Recording and reporting of (S)AEs protocol".

Operational definitions for AEs and SAEs

Adverse Events (AEs) are defined by the Health Research Authority (HRA) as any untoward medical occurrence, unintended disease or injury, or untoward clinical signs in participants, whether or not related to the treatment. In addition, issues specific to psychological therapies will also be monitored, namely: clinically significant increases in mental health or physical health problems; risky or problematic behaviours; harm to self/others, including suicide attempts; harm from others; and emergency room visits or crises. Clinically significant increases will be operationalised as an unresolved exacerbation requiring increased involvement from the care team, e.g. alterations to treatment plan. Distress or complaints associated with therapy, completion of assessment measures, or any other trial procedure would also constitute AEs.

The causes for the AEs will also be recorded and monitored. For each AE, the following potential reasons will be identified:

- Victimisation: aggressive behaviour, sexual abuse/assault, physical abuse/assault, emotional abuse/psychological maltreatment, exploitation, and other victimisation
- Mental health/psychological difficulties: excessive use of substances, exacerbation of mental health difficulties, suicidal ideation, and other psychological difficulties
- Trial procedures: group allocation, assessments, or therapy
- Physical health, including exacerbation of presenting FND symptoms, new neurological or other physical symptoms, and COVID-19 infection.
- Accidents or natural disasters
- Other.

AEs will be initially assessed at three levels of severity; mild, moderate and severe, which reflect the impact of the event on the person at the time. Please note there is a distinction between "severe" and "serious". Seriousness is the criteria for defining regulatory reporting obligations:

Serious Adverse Events (SAEs) are defined as:

- Death and life-threatening events (Category A)
- Incidents which acutely jeopardise the health or psychological wellbeing of the individual, resulting in immediate hospital admission and/or persistent or significant disability or incapacity (category B)
- Resulting in injury requiring immediate medical attention (category C).

For peer review only - http://bmjopen.bmj.com/site/about/guidelines.xhtml

All AEs and SAEs will be reported to the TM and CI. A summary of (S)AEs will be presented at each Trial Management Group (TMG) and Trial Steering Committee (TSC) meeting. Urgent actions concerning participant and staff safety, communication with others, and clinical care will be immediately addressed by the CI and reported to the TMG.

AEs will be categorised for severity and seriousness by the TM and CI. SAEs will be further reviewed for relatedness to trial procedures and unexpectedness by the CI initially, and additionally by the chair of the TSC.

Relatedness and unexpectedness of an event to the intervention will be judged based on the following:

1. Related: the event resulted from administration of any of the research procedures, judged according to a temporal relationship (i.e., Serious Related Events; SREs);
2. Unexpected: the event is unexpected or unexplained given the participant's clinical course, previous conditions and history, and concomitant treatments (i.e., Unexpected Serious Related Events; USREs)

Recording and reporting of (S)AEs

Best practice, professional guideline and local NHS policies for monitoring mental state and risk will be followed throughout the participants' involvement in the trial and will be facilitated by the trial being embedded in the Neuropsychiatry Service. The safety of the intervention will be monitored closely during therapy sessions and through contacts with the Neuropsychiatry Service.

The occurrence of AEs will be monitored actively and systematically and recorded by the RA, therapists, and clinical team. All AEs and participant withdrawals will be recorded and monitored by the trial team. If indicated, the TM and CI will review the clinical notes and contact clinicians for any important additional information. At the completion of the trial, all clinical notes will additionally be checked, for the total duration of enrolment, for any previously undisclosed record of AEs. This extra procedure is to ensure completeness of records and to address the possibility of an increased likelihood of disclosure of AEs in the EMDR+NPC condition, as a result of greater frequency of contact and the therapeutic relationship. In the final reports of the trial, the numbers, types and severity of AEs by trial condition, as well as discontinuations, will be reported.

Notification of deaths

All deaths will be reported to the sponsor within one month regardless of whether the death is related to the trial, disease or an unrelated event.

For peer review only - http://bmjopen.bmj.com/site/about/guidelines.xhtml

Monitoring, Audit & Inspection

A trial manager is in post to coordinate the day-to-day running of the trial.

Trial Management Group (TMG):

Dr Sarah Cope – Chief investigator

Prof. Mark Edwards – Co-investigator

Dr Sharif El-Leithy – Co-investigator

Dr Jared Smith – Co-investigator

Dawn Golder – PPI representative

Dr Patricia Hogwood – PPI representative

Kati Jane Turner – PPI representative

Dr Serena Vanzan – Trial Manager

Caitlin Pentland – Research Assistant

A Sponsor representative will observe each meeting.

TMG Terms of Reference

1. To review and provide feedback on the study protocol, outcome measures and recruitment documents (including any amendments)
2. To support the progress of the trial towards its interim and overall objectives and against the study timescale
3. To agree on a trial monitoring plan
4. To advise on participant recruitment and retention
5. To facilitate data analysis
6. To support the troubleshooting of any hindrances to the timely completion of the trial
7. To review the study outcomes
8. To discuss future developments of the study
9. To report to the Trial Steering Committee (TSC)

Trial Steering Committee (TSC)

The TSC will oversee the study on behalf of the of the trial Sponsor and Funder and ensure that the study is conducted within appropriate NHS and professional ethical guidelines. It will provide advice on all appropriate aspects of the project; will oversee progress of the trial, adherence to the protocol, participant safety and the consideration of new information of relevance to the research question; will ensure the rights, safety and well-being of the

For peer review only - http://bmjopen.bmj.com/site/about/guidelines.xhtml

participants are given the most important considerations; will ensure appropriate ethical and other approvals are obtained in line with the project plan; will agree proposals for substantial protocol amendments and provide advice to the Sponsor and Funder regarding approvals of such amendments.

The TSC comprises of:

Dr Tim Nicholson– Independent Chairman

Prof Richard Brown – Independent Expert

Dr Sarah Cope – Chief investigator

Dr Serena Vanzan – Trial Manager

Steve Portelly – Independent PPI representative

Kirsty Griffin – Independent PPI representative

A Sponsor representative will observe each TSC meeting.

TSC Terms of Reference

1. To review and approve the study protocol and recruitment documents (including any amendments)
2. To monitor and supervise the progress of the trial towards its interim and overall objectives and against the study timescale
3. To ensure all trial activities are conducted in line with GCP guidelines
4. To inform the funder annually on the progress of the trial
5. To advise the funder on publicity and the presentation of all aspects of the trial

Protocol compliance

A study related deviation is a departure from the ethically approved study protocol or other study document or process (e.g. consent process or administration of study intervention) or from Good Clinical Practice (GCP) or any applicable regulatory requirements. Any deviations from the protocol will be documented in a protocol deviation form and filed in the study master file.

The reporting procedure for any potential protocol breaches will be reported to the CI and sponsor. Any potential breach will be assessed by the CI and their supervisor(s), and reported as below where this may be a potential serious breach.

Notification of Serious Breaches to GCP and/or the protocol

A “serious breach” is a breach of the protocol or of the conditions or principles of Good Clinical Practice which is likely to affect to a significant degree: (a) the safety or physical or mental integrity of the trial subjects; or (b) the scientific value of the research.

For peer review only - http://bmjopen.bmj.com/site/about/guidelines.xhtml

In the event that a serious breach is suspected, the Sponsor will be contacted within 1 working day. In collaboration with the CI, the serious breach will be reviewed by the Sponsor and, if appropriate, the Sponsor will report it to the approving REC committee and the relevant NHS host organisation within seven calendar days.

Amendments

In the event of the sponsor wishing to make substantial amendments to the REC application or any supporting documentation, they will submit a valid notice of amendment to the REC for consideration.

The SWLSTG NHS and R&D departments will be notified.

The sponsor in collaboration with CI and investigators who contributed to the protocol will determine whether an amendment to the protocol is required and is substantial.

The CI will seek guidance from the local REC office. Changes that are deemed to be substantial will be communicated to the REC and SWLSTG NHS R&D department via the notification system in IRAS.

Any amendments will constitute a new approved version and will have a new label and date in order to distinguish it from the past protocol.

For peer review only - http://bmjopen.bmj.com/site/about/guidelines.xhtml

Data Management Plan (DMP)

Randomised feasibility study of eye movement desensitisation and reprocessing therapy (EMDR) for functional neurological disorder (FND) (MODIFI)
COPS1001

DMP SIGN-OFF SHEET

Trial Forms	Version Number	Version Date
MODIFI_Research protocol_v3.1_23Nov2022	3.1	23/11/2022
MODIFI_Participant invite letter_v1.2_23Nov2022	1.1	23/11/2022
MODIFI_Consent Form_v1.3_23Nov2022	1.3	23/11/2022
MODIFI_PIS_v3.1_23Nov2022	3.1	23/11/2022
MODIFI_GP letter_v2.0_07Sep2022	2.0	07/09/2022

I have reviewed the trial's DMP and approved the use of the above documents

Name	Position	Signature	Date
Sarah Cope	Chief Investigator		04/11/2022
Serena Vanzan	Trial Manager		03/11/2022
Caitlin Pentland	Research Assistant	Caitlin Pentland	09/11/2022
Jared Smith	Trial Statistician		31/01/2023

Comments

For peer review only - http://bmjopen.bmj.com/site/about/guidelines.xhtml

Randomised feasibility study of eye movement desensitisation and reprocessing therapy (EMDR) for functional neurological disorder (FND) (MODIFI)

COPS1001

1. Trial information

Trial type	Non-CTIPM Intervention - Feasibility
Study Sites	Single-site (SWLSTG)
Total sample size	50
Total duration of study<br><br>(months)	Trial start date _01/06/2022__<br><br>Planned duration for recruitment (months)___12____<br><br>Planned duration of follow-up (months)___9____<br><br>Total Duration ___36____Months
Study objective and design	The study aims to evaluate the possibility of delivering, and potential benefit, of EMDR for FND.<br>We will recruit 50 participants who have specific functional neurological symptoms: weakness, walking difficulties, jerks, shaking, and/or seizures from a Neuropsychiatry Service. Participants will be randomly allocated to EMDR, and routine medical appointments, or routine medical appointments alone. Those allocated to EMDR will be offered 8-16 weekly therapy sessions, completed within 6 months, and a follow-up session 1 month after therapy has ended. Participants will be able to choose whether they attend therapy in-person or via an online video conferencing platform. Participants will complete questionnaires regarding their health-related functioning, FND, mental health, and healthcare utilisation. These questionnaires will be completed at the beginning, and at 3 months, 6 months, and 9 months.<br>Some participants will attend interviews about their experiences of treatment.
Primary outcome measure	This is a feasibility study so no primary outcome measure has been chosen, as one of the study's aims is to investigate the value of a range of outcome measures, to determine the outcome measure with greatest symptom improvement and the required sample size, for a

For peer review only - http://bmjopen.bmj.com/site/about/guidelines.xhtml

	substantive RCT.
Secondary outcome measures	Health-Related Quality of Life/Functioning<br>FND<br>Depression and anxiety<br>PTSD<br>Dissociation<br>Service utilisation and other cost variables<br>Improvement<br>Beliefs about diagnosis and intervention<br>Measure of satisfaction
Interim analysis	Yes/**no**, if yes when_____________(Months from baseline)

2. Trial personnel and contact details

This section details the name, their position in the trial, email address, telephone/fax number for all staff involved in the trial including the sponsor. The trial coordinator/trial manager, the investigators, study staff involved in the data management (including computing staff responsibilities for maintaining hardware and software), the monitors and anyone else associated with the trial at each site.

2.1 Sponsor site personnel (add or remove accordingly)

Role	Name	Organisation	Contact Details
Sponsor	SWLSTG R&D	SWLSTG	ResearchDevelopment@swlstg.nhs.uk
Chief Investigator	Sarah Cope	SWLSTG	Sarah.Cope@swlstg.nhs.uk
Trial Manager	Serena Vanzan	SWLSTG	Serena.Vanzan@swlstg.nhs.uk
Research Assistant	Caitlin Pentland	SWLSTG	Caitlin.Pentland@swlstg.nhs.uk
Co-Investigator and Trial statistician	Jared Smith	SGUL	jasmith@sgul.ac.uk
Co-Investigator	Sharif El Leithy	SWLSTG	Sharif.Elleithy@swlstg.nhs.uk
Co-Investigator	Mark Edwards	KCL	mark.j.edwards@kcl.ac.uk
Primary contact for DM issues	Serena Vanzan	SWLSTG	Serena.Vanzan@swlstg.nhs.uk
Secondary contact for DM issues	Sarah Cope	SWLSTG	Sarah.Cope@swlstg.nhs.uk

For peer review only - http://bmjopen.bmj.com/site/about/guidelines.xhtml

3. Milestones

3.1 Study Milestones

Milestone	Date/Estimated Date
Date funding confirmed	22.03.2021
Date and version number of approved protocol	05.10.2022, v3.0
Date and version number of final protocol amendment(s)	03.11.2022, Am01
Date/version number of final approved CRF	No approval required
Release date/version number of final database	01.12.2022
Date DMP signed off	31.01.2023
Date of first participant first visit (FPFV)	19.12.2022
Date last participant last visit (LPLV)	

3.2 Proposed Data Milestones

Milestone	Date/Estimated Date
Data entry to commence	w/s 07.11.2022
Date of interim data partial lock if applicable	n/a
Data entry completed	Est Jun 2024
Last query resolved in the system	Est Aug 2024
Date of database final lock	Est Aug 2024

4. Data collection & data entry system

4.1 *Detail how data will be collected and entered from each site, whether to* ***complete the paper CRFs*** *or how to enter data electronically from each site.*
CRFs and outcome measures (questionnaires) will be completed electronically via the software REDCap, either by the RA (CRFs) or the participant (questionnaires). Where the participant will prefer paper questionnaires, these will be provided and returned via post, and the RA will enter the responses onto REDCap. If the participant requires face-to-face appointments, the RA will complete the CRFs and questionnaires either directly on REDCap or, if this is not possible, on paper and will then transfer the data onto REDCap.
Participants will also answer a few questions about their symptoms via the m-Path app.

4.2 *Provide details on the system used for data entry*
The REDCap online software will be used for collecting CRF and quantitative data. The m-Path app will be used for collecting information about participants' main symptoms (participant-completed).

4.3 *Outline who will be conducting the data entry from each site?*
The RA and the participants themselves.

4.4 *Outline whether single or double data entry will be carried out for all sites*
Single.

For peer review only - http://bmjopen.bmj.com/site/about/guidelines.xhtml

4.5 *If double data entry is required describe the process and whether a 100% of CRFs or a sample of CRFs will be double entered. Outline how the two entries will be compared, who will carry out the comparison and how the results will be dealt with.*
N/a

4.6 *Outline the checks undertaken for outcome measures*
The TM will perform regular self-monitoring visits in which the data entered for a proportion of participants will be checked for completeness, accuracy and timely completion. The dataset from the m-Path app will be downloaded and backed up weekly by the RA, and will be inspected by the TM during monitoring visits.

4.7 *Provide details on how the data will be centralised*
N/a – one site.

5. Data checks & data validation for each site

5.1 *Outline who will perform data cleaning, missing data checks, consistency checks, range checks and logic, and how these are checked at each site?*
The TM will perform regular self-monitoring visits in which the data entered for a proportion of participants will be checked for completeness, accuracy and timely completion. In addition, value range restrictions will be applied in REDCap and the m-Path app so that no values outside the available range can be entered when completing closed questions.

5.2 *Describe how regularly the data will be checked*
Monthly.

5.3 *Provide contact person for each site for data queries if data managed centrally*
N/a

5.4 *Describe the data flow from each site to central data centre, and who will conduct the overall data check*
N/a – one site.

5.5 *Detail the* ***flow of the data*** *from the field to the final storage for each site*
Data will be collected directly on study database (REDCap) or m-Path app. Any data from paper version of the CRFs and questionnaires will be entered on REDCap by the RA within 24 hours of collection.

6. AE and SAE data handling

Outline how the AE data will be collected by each site and collated for all sites at the end of the trial.
N/a – one site.

7. Partial and/or final data check and database lock

Detail if an interim analysis is planned in the trial protocol, stating the time point and whether the database will be partially locked for the interim analysis.
N/a

Detail the process for partial and final data checks and data lock, outline who checks the data and who will sign off for partial/final data lock form(s)?

For peer review only - http://bmjopen.bmj.com/site/about/guidelines.xhtml

The database will be locked after the following actions have been confirmed as completed by the TM:

1. All CRF data have been collected and entered onto REDCap.
2. All queries identified during regular data checks and self-monitoring visits have been resolved/clarified.
3. All missing information has been confirmed as being not available (as opposed to not entered).
4. All monitoring visits (incl. close-out) have been performed, and outstanding actions completed.
5. A final data quality check has been performed.

The TM will be responsible for locking the REDCap database (notifying the CI and Sponsor), send the REDCap and m-Path datasets to the trial statistician (with treatment allocation coded to prevent unblinding), and save a copy of the full databases in the TMF.

8. Data security and transmission between sites

Provide details on the data security procedures for transmitting data between sites
To comply with SWLSTG's Information Governance regulations, the datasets will be sent via an encrypted email to the trial statistician.

9. Data export & analysis

Explain how data will be exported and who the data should be sent to for data analysis.
The REDCap database with demographic and questionnaire data and data from m-Path app will be exported in CSV format and sent via encrypted email to Jared Smith, Trial Statistician and Co-investigator.

10. Data Back-up and archiving

Describe procedures in place to ensure data protection including back-up system (if you don't do this you could lose the data!)
The REDCap database and m-Path data will be downloaded and backed up weekly on a Trust external hard drive. The TMF will be saved on the Trust's shared drive, which is backed up every night, in a restricted folder dedicated to the study. In addition, the TMF will also be backed up once a week on the Trust encrypted external hard drive.
The study will be archived by the R&D team on the SWLSTG R&D shared drive.

For peer review only - http://bmjopen.bmj.com/site/about/guidelines.xhtml

Participant Information Sheet

South West London and
St George's Mental Health
NHS Trust

Participant Information Sheet

MODIFI: A feasibility study of eye MOvement Desensitisation and reprocessing therapy (EMDR) for Functional neurological disorder (FND)

We would like to invite you to take part in this research. Before you decide whether you want to, it is important for you to read the following information carefully and discuss it with others if you wish. Please ask us if there is anything that is not clear or if you would like more information.

Brief summary

This research aims to evaluate the possibility of delivering, and potential benefit, of eye movement desensitisation and reprocessing therapy (EMDR) as a psychological therapy for functional neurological disorder (FND). This is a feasibility study which means that it will not have enough participants to demonstrate statistically whether EMDR is helpful or not, but it will test out the study procedures (such as recruitment and therapy attendance) and look at whether it seems to benefit participants or not. If the study shows that it is feasible and potentially beneficial, a larger trial will be designed. A larger trial will be able to find out whether receiving EMDR, in addition to routine neuropsychiatric care, is more helpful to patients than neuropsychiatric care alone. In order to receive funding for a larger trial, this feasibility study needs to be successful.

The feasibility study will recruit 50 participants with specific functional neurological symptoms (weakness, walking difficulties, jerks, shaking, and/or seizures) from the Neuropsychiatry Service at St. George's Hospital. Participants will be allocated randomly (i.e. by chance, using a computer programme) to either EMDR and routine neuropsychiatry appointments; or to routine neuropsychiatry appointments alone.

Background

Functional Neurological Disorder (FND) is a problem with the functioning of the nervous system and how the brain and body send and receive signals, rather than due to neurological disease or injury. FND causes a range of neurological symptoms, such as seizures, shaking, weakness, and paralysis. The symptoms are associated with significant distress and disability. Treatment for FND in the United Kingdom is limited, and the evidence-base for treatment is poor, despite it being a common condition.

A psychological therapy called cognitive-behavioural therapy (also commonly known as CBT) has been found to be beneficial, but it does not help everyone. EMDR is a psychological therapy that has combined ideas from several therapies, including cognitive-behavioural, psychodynamic, and experiential. The basic idea behind EMDR is that psychological distress originates from upsetting memories in a person's past, and that targeting those key memories will result in a reduction of distress. EMDR is a well-established treatment for PTSD, but there is increasing evidence that is also helpful for many other difficulties, including depression, anxiety disorders, tinnitus, and chronic pain. There is a small amount of case study evidence that EMDR can be useful for treating FND, but proper scientific testing is needed.

EMDR should only be delivered by suitably qualified psychologists or psychological therapists, who have undergone the EMDR training required and who receive EMDR supervision. Within EMDR, key memories or images are identified that could be targeted in therapy. Each EMDR processing session follows a standardised protocol. As part of this protocol, a patient is asked to bring the target image to mind, with an identified negative cognition, notice where they are feeling the distress in their body, and follow the clinician's fingers with their eyes. After each set of eye movements, they are asked what they noticed, and importantly, without discussion, they are told to "go with that" alongside the eye movements. It may involve multiple sessions for the distress to reduce sufficiently. It should be

For peer review only - http://bmjopen.bmj.com/site/about/guidelines.xhtml

NHS
South West London and
St George's Mental Health
NHS Trust

noted that alternative "bilateral stimulation" tasks, instead of eye movements, can be used, such as alternate tapping or audio-tones. There are several theories regarding why the eye movements, or dual-attention on another task, may facilitate emotional processing, but like other psychological therapies, the specific mechanisms of how it works are not clear. It may be that the eye movements (or other task) allow the person to stay in an optimal zone for emotional processing (i.e. not too distressed). Another theory is that engaging in two simultaneous tasks taxes working memory, which has limited capacity. Therefore "bilateral stimulation" whilst focusing on the target memory/image results in less space for the distressing memory/image, and it is stored back in memory less vividly with reduced disturbance.

Why have I been chosen?

We are recruiting people diagnosed with FND by a neurologist, who have been referred to the Neuropsychiatry Service. During the appointment you attended with a neuropsychiatrist, they identified that you experienced specific functional neurological symptoms, such as weakness, walking difficulties, jerks, shaking, and/or seizures, and that you were likely suitable to take part in this study. They asked you whether you would be willing to be contacted about the study, and following your consent, passed your contact details on to the research team.

What will I have to do?

If you are interested in taking part in the study, please let the research team know by emailing XXXX or telephoning XXXX. The research assistant will contact you to arrange a convenient time to attend a screening assessment in order to check you are able to participate. The screening assessment can be carried out in-person at St. George's Hospital or remotely via MS Teams, whichever is best for you.

If you choose to attend the screening assessment, it will last between 30 minutes and 2 hours. During the appointment, you will be able to ask any questions. You will be asked to sign a consent form indicating that you agree to take part in the study. Agreement to take part will include you agreeing to therapy sessions to be video recorded if you are allocated to the group of participants attending EMDR alongside your routine neuropsychiatry appointments (please see below section on data protection and confidentiality for details).

You will then be asked to complete a questionnaire that asks you whether you have experienced any specific potentially traumatic life events in your lifetime, such as being assaulted, experiencing a life-threatening illness, being in an accident or being bullied. To take part in this study, you will need to have experienced at least 1 potentially traumatic event in your lifetime. You will not be asked to discuss the event(s) in the screening assessment, only indicate whether or not any have happened to you. Following this you will be asked to complete another questionnaire that examines any instances of dissociation you may have had over the previous 4 weeks. Dissociation is a broad term that refers to experiences of disconnection or lack of integration in your experience, such as your body feeling like it was someone else's, things around you suddenly seeming strange, or feeling like two or more people were fighting or arguing inside of yourself. There is a particular type of dissociation called identity disturbance and we will test for this as part of the assessment, as we will not be inviting anyone with this condition to take part in the research.

If the screening assessment tells us that you are eligible to take part in the study, we will ask you to complete additional questionnaires.

If you are not eligible to take part in the study and are interested in attending psychological therapy, you will be offered an appointment with Dr Cope, Principal Clinical Psychologist in the Neuropsychiatry Service, who will be able to discuss your current difficulties and psychological therapy options with you.

South West London and St George's Mental Health NHS Trust

What would taking part involve?

Completing questionnaires

Once it is confirmed you are eligible to take part in the study, you will be asked to complete additional questionnaires regarding your health-related functioning, mental health, and use of healthcare services. You will also be asked to complete questions on your demographics (e.g. relationship status, living arrangements, employment status), your medical history, current medication, and any previous psychological therapy you have attended, e.g. type, number of sessions, and focus of intervention (if known).

You will also be asked to identify the FND symptoms, e.g. seizures, tremor, limb weakness, tingling/numbness, gait disturbance (maximum two symptoms) that have the worst impact on your quality of life. You will then be given instructions regarding rating each symptom daily for 2 weeks. This can be done via an App or on paper if you prefer.

These are called "baseline measures", and you will be asked to complete questionnaires at 3 more time points: 3 months, 6 months and 9 months after your screening appointment. The research assistant will contact you at these time points to arrange completion of the questionnaires.

Randomisation (Allocation to treatment group)

Following your consent and completion of the baseline measures, you will be allocated to either EMDR plus routine appointments with your allocated neuropsychiatrist (EMDR+NPC), or routine neuropsychiatric appointments alone (NPC). You will be allocated at random by a computer programme: the research team will not be able to predict or change which group you will be assigned to. You will be told which group you have been randomly allocated to by the Trial Manager. The research assistant will not be told which group you have been allocated to, and it is important that you do not let them know which group you are in, at any point in the study. This is because if they know which group you are allocated to, it may influence how they support you in the completion of the questionnaires completed at different time points in the research.

Treatment groups

You will be invited to routine appointments with your assigned neuropsychiatrist (NPC). It is likely you will be invited to 1-3 routine neuropsychiatric appointments during the research. Each of these routine medical appointments lasts for 30 minutes. Your neuropsychiatrist may refer you to educational FND groups run in the service or for treatments for any other difficulties you may have.

If you are allocated to EMDR+NPC, you will be contacted by a psychological therapist to arrange your first EMDR appointment. Your EMDR appointments can be held in-person at St. George's Hospital or virtually via MS Teams (your choice). You will be offered up to 16 sessions of EMDR, with each session lasting between 60 and 90 minutes (treatment duration will depend on your individual needs). The sessions will need to be completed within 6 months, and ideally you will meet with your therapist weekly. A minimum number of 8 attended EMDR sessions will count as "completed treatment". An optional follow-up session will be offered 1 month after treatment completion. Each of your sessions will be video recorded. This is to check the therapist is following the treatment protocol and for the therapist to review sessions in their clinical supervision.

Whether you are allocated to EMDR+NPC or NPC alone, we ask that during the study period (9 months), you do not start attending any other individual psychological therapies focused on FND or specific FND treatments, such as inpatient treatment or specialist intensive physiotherapy for FND. Your neuropsychiatrist or other doctors involved in your care can still refer you to these treatments during the trial period (if needed); we just ask you not to start any of them during the time you are part of the study if possible. If you do start attending individual psychological therapy for FND which is not part of the study, or the specific FND treatments mentioned, you will not be able to continue to take part in the study, and any data collected up until that point will be used for analysis. This is because we would like to compare EMDR+NPC to NPC alone, and if you receive another specific FND

For peer review only - http://bmjopen.bmj.com/site/about/guidelines.xhtml

South West London and St George's Mental Health NHS Trust

treatment in the same time period, we will not be able to know whether any changes are due to the treatment we are offering or the treatments you are receiving elsewhere.

Travel expenses and payment for participation

You will be reimbursed for research-related travel costs, up to the value of £20 per appointment (if you choose to attend appointments in-person, rather than virtually). Receipts/proof of travel will need to be provided.

If you are enrolled onto the trial, you will be offered a £25 incentive 9-months after consenting to participate, irrespective of whether you complete the trial or not.

Do I have to take part?

No. It is up to you to decide whether to take part. If you choose not to take part, it will not affect any routine care that you receive.

How long does the study last?

If you take part, your participation in the trial will end after 9 months, once the final assessment measures have been completed. If you take part, you will also be asked whether you would be willing to be contacted after completion of the study to potentially provide additional data for a prospective full-scale study, and/or be invited to participate in future studies.

When all participants have completed the study, some people will be invited to attend interviews regarding their experiences of the study. If you are invited to attend an interview, this will be entirely voluntary, and you will be given further information beforehand so that you can decide whether you would like to be interviewed or not.

Following your participation in the trial, your clinical care will continue to be provided by the neuropsychiatry service, if needed, and will be assessed on your individual basis.

What are the possible benefits of taking part?

We cannot guarantee any specific treatment benefits, but it is hoped that those allocated to EMDR + NPC will benefit from the therapy. Taking part in this research will contribute to the evidence-base regarding treatments for FND. Contributing to research on FND treatments has the potential to help many people with FND, as the results of this study will likely inform treatment provision for FND.

What are the possible disadvantages and risks of taking part?

Attending mental health treatments like neuropsychiatric appointments and/or psychological therapy like EMDR may result in an increase in distress and/or deterioration in physical or mental health. However, these potential changes are usually temporary. They may occur as a result of discussing difficult events, emotions and symptoms in the appointments. If you are randomly selected for EMDR+NPC, it is possible that during your EMDR sessions, focusing on a distressing memory may cause you to experience an increase in the physical sensations or emotions associated with that memory. However, this is likely to be temporary and it is hoped that any potential discomfort will be outweighed by the potential benefits of EMDR. Irrespective of which treatment group you are allocated to, if you experience an increase in distress and/or deterioration in your physical or mental health, you will be supported by the neuropsychiatry healthcare professional(s) involved in your care, and if needed, signposted or referred to relevant organisations or support resources.

South West London and St George's Mental Health NHS Trust

What if I don't want to carry on with the research project?

It is up to you to decide whether to take part or not; choosing not to take part will not disadvantage you in any way or affect any treatment you are receiving outside of the study. You are free to withdraw at any time without giving a reason. If you withdraw, it will not affect any routine care that you receive. If you withdraw early from the study, any data you provided up until that point will be used for analysis.

What if something goes wrong?

If you have a concern about any aspect of this study, you should contact the trial manager Dr Serena Vanzan or chief investigator Dr Sarah Cope [contact number XXXX, email modifi@swlstg.nhs.uk; sarah.cope@swlstg.nhs.uk]. If you are not satisfied following this, you can contact the Sponsor's Office by emailing ResearchDevelopment@swlstg.nhs.uk. If you remain unhappy and wish to complain formally, you can do this by contacting complaints@swlstg.nhs.uk. Further details can be obtained from https://www.swlstg.nhs.uk/contact-us/complaints. Or you can contact the Trust's Patient Advice and Liaison Service (PALS). Tel: 0203 513 6150 (Monday - Friday 9.30am to 4.30pm) or email pals@swlstg.nhs.uk.

In the event that something does go wrong and you are harmed during the research and this is due to someone's negligence then you may have grounds for a legal action for compensation against SWLSTG NHS Trust, but you may have to pay your legal costs. The normal National Health Service complaints mechanisms will still be available to you.

Will my taking part in this study be kept confidential?

All the information that we collect about you during the course of the research will be kept strictly confidential. The only limits to this confidentiality are that if you share information that suggests you or someone else is at risk of harm, or if you disclose a serious crime has been committed. If this happens, the researcher or clinician will need to share this information with those directly involved in your care. The researcher or clinician would try to discuss this with you before confidentiality is broken.

You will not be able to be identified in any ensuing reports or publications. All data will be pseudonymised. This means that you cannot be personally identified. Each participant will be assigned a unique screening number upon referral to the study. This number will be written on all eligibility measures and databases. A unique trial identification number will then be issued following your consent to take part. This number will be written on all clinical assessment forms/datasheets and databases used to record data on participants. All data will be kept secure at all times and maintained in accordance with General Data Protection Regulation (GDPR, 2018) requirements and archived according to clinical trial Good Clinical Practice regulations. Only researchers working with the Chief Investigator (Dr. Cope) will have access to the data. The data custodian for this project will be Dr. Cope. The data will be retained for 10 years after completion of the study.

How will we use information about you?

We will need to use information from your medical records for this research project. This information will include your:

- NHS number
- Hospital number
- Name
- Contact details
- Date of birth
- Ethnicity
- Gender
- Relationship status
- Employment status
- Presence of dependent(s)
- Presence of a carer

For peer review only - http://bmjopen.bmj.com/site/about/guidelines.xhtml

- Receipt of state benefits
- Current medication
- Medical history

People will use this information to do the research or to check your records to make sure that the research is being done properly. People who do not need to know who you are will not be able to see your name or contact details. Your data will have a code number instead. We will keep all information about you safe and secure.

Once we have finished the study, we will keep some of the data so we can check the results. We will write our reports in a way that no-one can work out that you took part in the study.

What are your choices about how your information is used?

You can stop being part of the study at any time, without giving a reason, but we will keep information about you that we already have.

- We need to manage your records in specific ways for the research to be reliable. This means that we won't be able to let you see or change the data we hold about you.
- If you agree to take part in this study, you will have the option to take part in future research using your data saved from this study. If you consent to this we may:
  - Use your data already collected for this study in future research.
  - Contact you regarding taking part in future research relating to this current study.

Where can you find out more about how your information is used?

You can find out more about how we use your information

- at www.hra.nhs.uk/information-about-patients/
- by contacting the trial team at modifi@swlstg.nhs.uk
- by sending an email to SWLSTG's Data Protection Officer, John Hughes, at john.hughes@swlstg.nhs.uk
- by ringing us on XXXX

Informing General Practitioner (GP)

If you choose to participate, your GP will be informed that you are participating in the study.

What will happen to the results of the study?

We will disseminate the results from this study in conferences, peer-reviewed journals, social media, and the website for the patient-led charity FND Hope.

We will email you a copy of the paper that presents the findings from this study once the paper has been published.

Who is organising and funding the research?

SWLSTG is the study sponsor. This project is funded by the National Institute for Health and Care Research (NIHR) under its Research for Patient Benefit (RfPB) Programme (Grant Reference Number NIHR202277).

For peer review only - http://bmjopen.bmj.com/site/about/guidelines.xhtml

South West London and St George's Mental Health NHS Trust

How have patients and the public been involved in this study?

The research design has been informed by a Public and Patient Involvement (PPI) meeting, where all participants had lived experience of FND. The research team has people with lived experience of FND. Two people are on the Trial Steering Committee and three other people are working with other members of the research team to make sure that the lived experience perspective is reflected in the research tools, methodology, analysis and dissemination. If the trial proves feasible, they will contribute to the design of a substantive study. The study has received guidance and support on PPI and lived experience involvement from the lived experience PPI lead for Research & Development at SWLSTG.

Who has reviewed the study?

All proposals for research using human subjects are reviewed by an Ethics Committee before they can proceed. This proposal was reviewed by West Midlands - Edgbaston Research Ethics Committee. This study has also been reviewed by the Health Research Authority.

Further information and contact details

Please contact modifi@swlstg.nhs.uk or telephone XXXX if you require further information about this study.

The Chief Investigator is Dr Sarah Cope (Principal Clinical Psychologist) and her contact details are:

Neuropsychiatry Service

2nd Floor Grosvenor Wing,

St. George's Hospital,

Blackshaw Road,

SW17 0QT

(T) 020 8725 3786

(E) sarah.cope@swlstg.nhs.uk

Thank you for reading this information sheet and for your consideration regarding taking part in this research study.

STANDARD PROTOCOL ITEMS: RECOMMENDATIONS FOR INTERVENTIONAL TRIALS

SPIRIT 2013 Checklist: Recommended items to address in a clinical trial protocol and related documents*

Section/item	ItemNo	Description	Page Number
**Administrative information**			
Title	1	Descriptive title identifying the study design, population, interventions, and, if applicable, trial acronym	i
Trial registration	2a	Trial identifier and registry name. If not yet registered, name of intended registry	ii
	2b	All items from the World Health Organization Trial Registration Data Set	
Protocol version	3	Date and version identifier	i
Funding	4	Sources and types of financial, material, and other support	i
Roles and responsibilities	5a	Names, affiliations, and roles of protocol contributors	i, 14
	5b	Name and contact information for the trial sponsor	14
	5c	Role of study sponsor and funders, if any, in study design; collection, management, analysis, and interpretation of data; writing of the report; and the decision to submit the report for publication, including whether they will have ultimate authority over any of these activities	14
	5d	Composition, roles, and responsibilities of the coordinating centre, steering committee, endpoint adjudication committee, data management team, and other individuals or groups overseeing the trial, if applicable (see Item 21a for data monitoring committee)	14, Supplementary Material (SM) p. 12-13
**Introduction**			
Background and rationale	6a	Description of research question and justification for undertaking the trial, including summary of relevant studies (published and unpublished) examining benefits and harms for each intervention	1-3
	6b	Explanation for choice of comparators	
Objectives	7	Specific objectives or hypotheses	3

For peer review only

Trial design	8	Description of trial design including type of trial (eg, parallel group, crossover, factorial, single group), allocation ratio, and framework (eg, superiority, equivalence, noninferiority, exploratory)	3

Methods: Participants, interventions, and outcomes

Study setting	9	Description of study settings (eg, community clinic, academic hospital) and list of countries where data will be collected. Reference to where list of study sites can be obtained	3
Eligibility criteria	10	Inclusion and exclusion criteria for participants. If applicable, eligibility criteria for study centres and individuals who will perform the interventions (eg, surgeons, psychotherapists)	4
Interventions	11a	Interventions for each group with sufficient detail to allow replication, including how and when they will be administered	4-6
	11b	Criteria for discontinuing or modifying allocated interventions for a given trial participant (eg, drug dose change in response to harms, participant request, or improving/worsening disease)	n/a
	11c	Strategies to improve adherence to intervention protocols, and any procedures for monitoring adherence (eg, drug tablet return, laboratory tests)	9
	11d	Relevant concomitant care and interventions that are permitted or prohibited during the trial	6
Outcomes	12	Primary, secondary, and other outcomes, including the specific measurement variable (eg, systolic blood pressure), analysis metric (eg, change from baseline, final value, time to event), method of aggregation (eg, median, proportion), and time point for each outcome. Explanation of the clinical relevance of chosen efficacy and harm outcomes is strongly recommended	6-7
Participant timeline	13	Time schedule of enrolment, interventions (including any run-ins and washouts), assessments, and visits for participants. A schematic diagram is highly recommended (see Figure)	Fig. 1
Sample size	14	Estimated number of participants needed to achieve study objectives and how it was determined, including clinical and statistical assumptions supporting any sample size calculations	10
Recruitment	15	Strategies for achieving adequate participant enrolment to reach target sample size	9

Methods: Assignment of interventions (for controlled trials)

Allocation:

Sequence generatio n	16a	Method of generating the allocation sequence (eg, computer-generated random numbers), and list of any factors for stratification. To reduce predictability of a random sequence, details of any planned restriction (eg, blocking) should be provided in a separate document that is unavailable to those who enrol participants or assign interventions	10
Allocation concealm ent mechanis m	16b	Mechanism of implementing the allocation sequence (eg, central telephone; sequentially numbered, opaque, sealed envelopes), describing any steps to conceal the sequence until interventions are assigned	10
Implemen tation	16c	Who will generate the allocation sequence, who will enrol participants, and who will assign participants to interventions	10
Blinding (masking)	17a	Who will be blinded after assignment to interventions (eg, trial participants, care providers, outcome assessors, data analysts), and how	10
	17b	If blinded, circumstances under which unblinding is permissible, and procedure for revealing a participant's allocated intervention during the trial	
**Methods: Data collection, management, and analysis**			
Data collection methods	18a	Plans for assessment and collection of outcome, baseline, and other trial data, including any related processes to promote data quality (eg, duplicate measurements, training of assessors) and a description of study instruments (eg, questionnaires, laboratory tests) along with their reliability and validity, if known. Reference to where data collection forms can be found, if not in the protocol	6-9
	18b	Plans to promote participant retention and complete follow-up, including list of any outcome data to be collected for participants who discontinue or deviate from intervention protocols	9
Data management	19	Plans for data entry, coding, security, and storage, including any related processes to promote data quality (eg, double data entry; range checks for data values). Reference to where details of data management procedures can be found, if not in the protocol	12, SM p. 15
Statistical methods	20a	Statistical methods for analysing primary and secondary outcomes. Reference to where other details of the statistical analysis plan can be found, if not in the protocol	10-11, SM p.8
	20b	Methods for any additional analyses (eg, subgroup and adjusted analyses)	
	20c	Definition of analysis population relating to protocol non-adherence (eg, as randomised analysis), and any statistical methods to handle missing data (eg, multiple imputation)	SM p. 8

Methods: Monitoring

Data monitoring	21a	Composition of data monitoring committee (DMC); summary of its role and reporting structure; statement of whether it is independent from the sponsor and competing interests; and reference to where further details about its charter can be found, if not in the protocol. Alternatively, an explanation of why a DMC is not needed	12, SM p. 12-13
	21b	Description of any interim analyses and stopping guidelines, including who will have access to these interim results and make the final decision to terminate the trial	n/a
Harms	22	Plans for collecting, assessing, reporting, and managing solicited and spontaneously reported adverse events and other unintended effects of trial interventions or trial conduct	SM p. 10-11
Auditing	23	Frequency and procedures for auditing trial conduct, if any, and whether the process will be independent from investigators and the sponsor	12

Ethics and dissemination

Research ethics approval	24	Plans for seeking research ethics committee/institutional review board (REC/IRB) approval	12
Protocol amendments	25	Plans for communicating important protocol modifications (eg, changes to eligibility criteria, outcomes, analyses) to relevant parties (eg, investigators, REC/IRBs, trial participants, trial registries, journals, regulators)	SM p. 14
Consent or assent	26a	Who will obtain informed consent or assent from potential trial participants or authorised surrogates, and how (see Item 32)	9, 12
	26b	Additional consent provisions for collection and use of participant data and biological specimens in ancillary studies, if applicable	n/a
Confidentiality	27	How personal information about potential and enrolled participants will be collected, shared, and maintained in order to protect confidentiality before, during, and after the trial	12
Declaration of interests	28	Financial and other competing interests for principal investigators for the overall trial and each study site	14
Access to data	29	Statement of who will have access to the final trial dataset, and disclosure of contractual agreements that limit such access for investigators	12
Ancillary and post-trial care	30	Provisions, if any, for ancillary and post-trial care, and for compensation to those who suffer harm from trial participation	12

For peer review only

Dissemination policy	31a	Plans for investigators and sponsor to communicate trial results to participants, healthcare professionals, the public, and other relevant groups (eg, via publication, reporting in results databases, or other data sharing arrangements), including any publication restrictions	12
	31b	Authorship eligibility guidelines and any intended use of professional writers	12
	31c	Plans, if any, for granting public access to the full protocol, participant-level dataset, and statistical code	n/a
**Appendices**			
Informed consent materials	32	Model consent form and other related documentation given to participants and authorised surrogates	SM p. 21
Biological specimens	33	Plans for collection, laboratory evaluation, and storage of biological specimens for genetic or molecular analysis in the current trial and for future use in ancillary studies, if applicable	n/a

*It is strongly recommended that this checklist be read in conjunction with the SPIRIT 2013 Explanation & Elaboration for important clarification on the items. Amendments to the protocol should be tracked and dated. The SPIRIT checklist is copyrighted by the SPIRIT Group under the Creative Commons "Attribution-NonCommercial-NoDerivs 3.0 Unported" license.

For peer review only

BMJ Open

MODIFI: Protocol for randomised feasibility study of eye movement desensitisation and reprocessing therapy (EMDR) for functional neurological disorder (FND)

Journal:	*BMJ Open*
Manuscript ID	bmjopen-2023-073727.R1
Article Type:	Protocol
Date Submitted by the Author:	21-Apr-2023
Complete List of Authors:	Cope, Sarah; South West London and Saint George's Mental Health NHS Trust,<br>Smith, Jared; St George's University of London, Population Health Research Institute; South West London and Saint George's Mental Health NHS Trust, Clinical Research Unit<br>El-Leithy, Sharif; South West London and Saint George's Mental Health NHS Trust, Traumatic Stress Service<br>Vanzan, Serena; South West London and Saint George's Mental Health NHS Trust, Clinical Research Unit<br>Pentland, Caitlin; South West London and Saint George's Mental Health NHS Trust, Clinical Research Unit<br>Pick, Susannah; Institute of Psychiatry Psychology and Neuroscience, Department of Psychological Medicine<br>Golder, Dawn; FND Hope UK<br>Hogwood, Patricia; N/A<br>Turner, Kati; N/A<br>Billings, Jo; University College London, Department of Psychiatry<br>Edwards, Mark; King's College London, Department of Basic and Clinical Neuroscience, Institute of Psychiatry, Psychology & Neuroscience
**Primary Subject Heading**:	Mental health
Secondary Subject Heading:	Neurology
Keywords:	NEUROLOGY, Adult psychiatry < PSYCHIATRY, Clinical trials < THERAPEUTICS

SCHOLARONE™ Manuscripts

MODIFI: Protocol for randomised feasibility study of eye movement desensitisation and reprocessing therapy (EMDR) for functional neurological disorder (FND)

By Sarah R. Cope[1*], Jared G. Smith[2,3], Sharif El-Leithy[4], Serena Vanzan[3], Caitlin Pentland[3], Susannah Pick[5], Dawn Golder[6], Patricia Hogwood, Kati Jane Turner, Jo Billings[7], & Mark J. Edwards[8]

[1]Neuropsychiatry Service, South West London & St. George's Mental Health Trust, St. George's Hospital, Blackshaw Road, SW17 0QT, UK

[2]Population Health Research Institute, St George's, University of London, Cranmer Terrace, Tooting, London, SW17 0RE, UK

[3]Clinical Research Unit, South West London & St George's Mental Health Trust, Springfield University Hospital, 61 Glenburnie Road, London, SW17 7DJ, UK

[4]Traumatic Stress Service, South West London & St. George's Mental Health NHS Trust, Springfield University Hospital, 61 Glenburnie Rd, London SW17 7DJ

[5]Department of Psychological Medicine, Institute of Psychiatry, Psychology & Neuroscience, King's College London, London, SE5 8AF

[6]FND Hope UK Registered charity in England & Wales 1173607 & Scotland SC048333

[7]Department of Psychiatry, University College London, Wing A, 6th Floor, Maple House, 149 Tottenham Court Road, London W1T 7NF, UK

[8]Department of Basic and Clinical Neuroscience, Institute of Psychiatry, Psychology & Neuroscience, King's College London, London, SE5 8AF

[*]Corresponding author: sarah.cope@swlstg.nhs.uk

Protocol: Version v3.1 23/11/2022

Word Count (excluding title page and abstract p. i-ii, Ethics Statements p. 14-15, tables & figures): 3997

Key Words: Functional neurological disorder, functional motor disorder, functional seizures, psychological therapy, eye movement desensitisation and reprocessing therapy, randomised controlled trial

For peer review only

Abstract

Introduction:

Functional neurological disorder (FND) refers to an involuntary loss of control over and/or aberrant perception of the body. Common presenting symptoms are functional (non-epileptic) seizures, and functional motor disorder, e.g. walking difficulties, weakness, or tremor. Greater access to effective treatments would lead to reduced distress and disability; and reduce unnecessary healthcare costs.

This study will examine eye-movement desensitisation and reprocessing therapy (EMDR) as a treatment for FND. EMDR is an evidence-based treatment for post-traumatic stress disorder (PTSD), but its use for other conditions is growing. An FND-specific EMDR protocol will be tested, and if the intervention proves feasible with promising clinical outcomes, progression to a substantive study could take place.

Methods and Analysis:

Fifty adult patients diagnosed with FND will be recruited. It will be a single-blind randomised controlled trial with two arms: EMDR (plus standard neuropsychiatric care; NPC) and standard NPC. The two groups will be compared at baseline (T0), 3 months (T1), 6 months (T2), and 9 months (T3). Measures of feasibility include safety, recruitment, retention, treatment adherence and acceptability. Clinical outcome measures will assess health-related functioning/quality of life, ratings of FND symptoms and severity, depression, anxiety, PTSD, dissociation, service-utilisation and other costs. Improvement and satisfaction ratings will also be assessed. Feasibility outcomes will be summarised using descriptive statistics. Exploratory analyses using (linear/logistic) mixed effect models will examine the rate of change in the groups' clinical outcome measures across the four time-points.

After the intervention period, a sample of participants, and clinicians, will be invited to attend semi-structured interviews. The interviews will be analysed using reflexive thematic analysis.

Ethics and Dissemination:

This study has been approved by the NHS West Midlands - Edgbaston Research Ethics Committee. Study findings will be published in open access peer-reviewed journals, presented at conferences, and communicated to participants and other relevant stakeholders.

Trial Registration:

NCT05455450 (www.clinicaltrials.gov)

Strengths and Limitations:

- This is a pragmatic randomised controlled trial embedded within an existing clinical service.
- The EMDR treatment is tailored for FND presentations.
- There has been Patient and Public Involvement (PPI) input from the design stage, and there is continued PPI involvement.
- The study will use a validated self-report measure to assess PTSD, rather than a clinical interview.

For peer review only

Introduction

Functional neurological disorder (FND) is a disorder at the interface between neurology and psychiatry. It refers to an involuntary loss of control over and/or aberrant perception of the body. Presenting symptoms can be wide-ranging with the commonest being functional (non-epileptic) seizures (FS) and functional motor disorder (mFND), e.g. walking difficulties, weakness, tremor. Risk and perpetuating factors for FND include traumatic experiences, affective disorders and experiencing chronic or acute illness. FND is one of the commonest diagnoses made in neurology, e.g. 16% of new patients in general neurology[1]; causes similar disability and impairment in quality of life as Parkinson's disease and multiple sclerosis, and high unemployment. [2] Lack of provision of assessment and treatment is associated with significant unnecessary costs, e.g. unnecessary referrals, investigations, and emergency department attendances.[3,4]

Accessing treatment is often difficult, and effective treatments for FND are still being established. Healthcare Improvement Scotland published guidance in 2012, recommending a stepped-care approach, whereby patients are assessed and diagnosed by a neurologist, and referred for relevant interventions (e.g. physiotherapy, psychology, psychiatry, occupational therapy) as required.[5] A cornerstone of FND treatment is effective communication of the diagnosis, and guidelines regarding management of FND have been published.[6,7] There have been consensus recommendations published for physiotherapy for mFND and occupational therapy.[8,9] The best evidence for mFND specifically comes from a feasibility study evaluating a specialist physiotherapy intervention compared to physiotherapy in the community, which reported positive outcomes in terms of recruitment, retention, acceptability, and clinically meaningful effect sizes.[10] A multicentre NIHR-funded RCT evaluating this is underway (International Standard Randomised Controlled Trials Number ISRCTN56136713). However, this intervention is not suitable for a large proportion of patients with mFND (only 32% of eligible patients met the inclusion criteria; most common reasons for exclusion were dominant persistent pain and psychological factors requiring treatment). There have been reports of beneficial outcomes following cognitive behavioural therapy (CBT) in uncontrolled studies for patients with mFND, but no controlled and adequately powered studies have been carried out.[11] For FS specifically, the multicentre CODES trial compared CBT plus standard medical care (SMC) to SMC alone. Although this study did not find a significant difference in monthly seizure frequency between the groups, they did find significant improvements for CBT on secondary measures (psychosocial functioning, psychological distress, and health-related quality of life).[12] Previous studies evaluating CBT for FS have reported significant improvements in seizure frequency.[13,14] More research regarding effective psychological treatments other than CBT for FND is needed, to inform delivery of treatment.

Cross-sectional studies suggest that lifetime traumatic/adverse experiences are higher in FND populations, when compared to healthy controls; in particular for those with FS, who also have higher incidences of post-traumatic stress disorder (PTSD).[15] The occurrence of severe life events immediately prior to symptom onset is significantly more frequent in those with mFND compared to psychiatric controls[16] and it has been proposed that there is a trauma subtype of FND.[17] Traumatic/adverse life events, including physical events such as injury or illness, are a risk factor and can be a trigger for developing FND. Mechanistic models of FND have been developed focusing on different levels of explanation from the neurobiological to the psychosocial. Neurobiological models have used predictive coding models of perception and movement control to suggest that symptoms in FND relate to the development of abnormal priors which are activated by misdirected attention towards the body.[18] This links closely with cognitive models suggesting that learned patterns of behaviour are triggered by abnormal threat processing.[19] Emotional dysregulation, abnormal interoceptive processing and alexithymia can all be integrated with such models, building a complex

For peer review only - http://bmjopen.bmj.com/site/about/guidelines.xhtml

picture of the biological and psychological processes that underpin FND.[20] This provides a scientific foundation for the development and application of specific psychological interventions to treat people with FND.

Eye-movement desensitisation and reprocessing therapy (EMDR) is an evidence-based treatment for PTSD, but its use for other conditions is growing, including treatment of somatic symptoms such as persistent pain and tinnitus.[21–24] EMDR follows a standard protocol (see Table 1).[25] Within EMDR, a target memory is brought to mind, whilst the clinician creates a distracting task that means the person's attention is divided between the memory and the present-focused task. Traditionally, eye-movements are used, but other tasks that create dual attention can be used, e.g. tapping. The working memory (WM) hypothesis suggests that focusing on the memory, whilst engaging in a competing task, results in the memory becoming less vivid and distressing. A recent systematic review, identifying 11 studies testing the WM hypothesis, concluded that bringing a distressing memory to mind, whilst engaging in a secondary task, results in reduced vividness and emotionality of the memory, and is associated with symptom reduction.[26]

Table 1: The 8 phases of EMDR according to the Standard Protocol (Shapiro 2018)

Phase	Description
1 - 2	Taking of patient history, assessment of suitability for EMDR, and preparation for the therapy.
3	Assessment of a target image whereby the patient is asked to bring a target memory to mind, identify the most upsetting image or moment, and identify the negative cognition about themselves that goes with that moment. They are also asked to identify a preferred cognition and rate their belief in that cognition. Additionally, they are asked to rate their subjective distress, identify the associated emotions, and locate where they feel the distress in their body.
4	Desensitisation phase: the patient is asked to bring the target memory to mind, with the negative cognition, notice where they are feeling the distress in their body, and follow the clinician's fingers with their eyes (or other alternating task that taxes working memory). After each set of eye movements, the patient is asked what they noticed, and importantly, without discussion, they are told to "go with that" alongside the eye movements. Once the distress has reduced sufficiently (this may involve multiple sessions), the clinician proceeds to the installation phase.
5	Installation phase: Preferred cognition is installed, again aided by eye-movements (or alternative).
6	Target any remaining distress in the body
7	Closure of the session
8	Assess previously targeted material and whether or not further processing is required

A systematic review of EMDR as a treatment for FND reported three case studies/series with all five cases presented having comorbid PTSD, of which four cases were successfully treated .[27–30] EMDR has been reported as a useful adjunctive therapy for two FND cases without PTSD, whereby both cases achieved resolution of FND symptoms and less distress.[31] EMDR can focus on specific past

For peer review only - http://bmjopen.bmj.com/site/about/guidelines.xhtml

adverse experiences that are contributing to pathology, memories associated with when FND symptoms began, current FND symptoms, and future predictions regarding symptoms. Theoretically, targeting distressing memories/images associated with FND could weaken cognitive representations of symptoms, and reduce threat associated with symptoms, meaning that representations of the symptoms are less easily triggered, resulting in fewer symptoms and less distress. EMDR is a therapy that can be tailored to the heterogeneous presentations of FND.

This study aims to evaluate the feasibility and acceptability of conducting a full-scale trial of EMDR for people diagnosed with FND. Feasibility will be assessed by examining recruitment rate, intervention adherence, and retention. Acceptability will be examined through attendance rates, satisfaction ratings, therapy fidelity ratings, and qualitative interviews with participants and treating therapists. Assessment of safety across the two arms will be compared. Examination of the completeness of outcome measures and variance in outcomes will be used to inform the design and power calculation of a future definitive trial.

Study Objectives:

i. Test the acceptability and feasibility of an FND-specific EMDR intervention protocol, delivered in-person or virtually. For a substantive RCT, the intervention will be subject to amendment based on the results of this trial.
ii. Investigate the value of a range of outcome measures, to determine the outcome measure with greatest effect size to enable a sample size calculation for a substantive RCT.
iii. Carry out semi-structured interviews with participants and therapists to explore experiences of EMDR and the trial; informing the intervention and design of a substantive trial.

Methods and analysis

This protocol is reported in accordance with the Standard Protocol Items: Recommendations for Intervention Trials 2013 statement.[32]

Study Design

This feasibility study is a single-blind randomised controlled trial (RCT) with two arms: EMDR (plus standard neuropsychiatric care (NPC)) and standard NPC only. The two groups will be compared at baseline (T0), 3 months (T1), 6 months (T2), and 9 months (T3). Fifty adult patients with a diagnosis of FND, confirmed by a neurologist according to standardised diagnostic criteria, will be recruited via a UK neuropsychiatry service. The research assistant (RA) and project statistician will be blind to treatment allocation. After the intervention period, semi-structured interviews will be carried with a proportion of participants and clinicians to explore their experiences and views about the trial.

Study Setting

This is a single-site study being carried out at a neuropsychiatry service, based at St. George's Hospital, Tooting, London, UK. The service is part of the South-West London and St. George's Mental Health NHS Trust (SWLSTG).

For peer review only - http://bmjopen.bmj.com/site/about/guidelines.xhtml

Public and Patient Involvement (PPI)

The research design has been informed by a PPI meeting (June 2020), where all five participants had lived experience of FND. Three PPI representatives join regular Trial Management Group (TMG) meetings, review participant literature, and will co-produce the interview schedules and contribute to the design of a substantive study. Two PPI representatives join the Trial Steering Committee (TSC).

Eligibility Criteria

Inclusion criteria:

(1) Predominant diagnosis of FS and/or mFND, with diagnosis confirmed by neurologist;

(2) Aged 18 years or over;

(3) Capacity to consent;

(4) Willingness to attend regular psychological therapy sessions;

(5) Reporting at least one traumatic event on the International Trauma Exposure Measure (ITEM).

Exclusion criteria:

(1) Non-English speaking;

(2) Current ongoing adversity that is likely to interfere with psychological therapy, e.g. domestic violence, homelessness, unresolved compensation claim/litigation;

(3) Predominant diagnosis of borderline personality disorder*;

(4) Predominant diagnosis of chronic pain condition*, e.g. fibromyalgia;

(5) Predominant diagnosis of chronic fatigue syndrome*;

(6) Diagnosis of a psychotic disorder;

(7) Diagnosis of dissociative identity disorder or score in clinical range on “identity disturbance” subscale of Multiscale Dissociation inventory;

(8) Uncontrolled epileptic seizures;

(9) Diagnosis of an eating disorder;

(10) Current severe self harm or strong suicidal ideation that requires secondary care mental health services input;

(11) Current alcohol or drug harmful use or dependence;

(12) Current diazepam use exceeding the equivalent of 10mg per day;

(13) Currently attending individual psychological therapy focused on FND or other specialist FND-specific treatment such as inpatient/outpatient multi-disciplinary treatment or intensive FND-specific physiotherapy

*Comorbid diagnosis is acceptable, as long as FND is the predominant difficulty

For peer review only

Study Interventions

EMDR plus standard neuropsychiatric care

The intervention group will be offered up to sixteen EMDR sessions, and a one-month follow-up session, as well as attending standard outpatient neuropsychiatric appointments (NPC). Participants will be given the choice of attending EMDR face-to-face or virtually via a video-consultation platform. Sessions will normally be attended weekly, with treatment completed within six months. Sessions will be 60-90 minutes long, in accordance with NICE guidance for PTSD.[33] A minimum of eight sessions was chosen based on patient feedback and previous research.[34] Optimum session duration and number will be examined as part of the trial. The optional follow-up session will occur one month after treatment completion.

EMDR for FND is a collaborative and individualised approach, following the standard EMDR protocol, but tailored for FND presentations. Treatment has three broad stages: Assessment, psychoeducation, target selection, and preparation for processing; Processing of targets; Ending of therapy. The initial sessions incorporate education regarding FND, anxiety and dissociation; formulating collaboratively with the participant regarding the development of FND symptoms; and giving a rationale for EMDR. In collaboration with the participant, target memories/images will be chosen, such as: (1) Distressing memories associated with the time when symptoms began; (2) Distressing memories from past events that may be relevant to their FND symptoms; (3) FND symptoms themselves, when present in session, or an image of them; (4) Distressing images about the future, e.g. image of having symptoms in front of others. EMDR therapy will follow the Therapy Protocol developed by the CI for this study. Table 2 shows an overview of treatment, with a guide regarding how many sessions per stage.

Table 2: Overview of EMDR for FND

Session(s)	Overview of Content
**1-3**	History taking and preparation for therapy (Phase 1-2 of standard EMDR protocol)<br><br>Formulating their FND presentation and increasing their understanding of FND<br><br>Collaboratively selecting target memories/images
**4-15***	Processing of EMDR targets using EMDR's three-pronged approach of past, present and future targets
**Final session**	End therapy
	An optional follow-up session can be offered 1 month after completing therapy (this does not count as one of the 8-16 sessions)
***Total number of sessions can be between 8-16. If completing by session 8, there will be fewer sessions in this phase of the intervention.**	

Standard Neuropsychiatric Care (NPC)

NPC is treatment-as-usual and will consist of 1-3 routine out-patient appointments with an assigned neuropsychiatrist in the trial period. Participants will not begin any FND-specific individual psychological therapy, inpatient/outpatient multi-disciplinary treatment or intensive specialist FND-

For peer review only - http://bmjopen.bmj.com/site/about/guidelines.xhtml

specific physiotherapy. If a participant begins FND-specific individual psychological or physiotherapy treatment in the trial period, they would not be able to complete the trial. Data from their last assessment point will be used for analysis. Participants can still attend psycho-educational interventions focused on FND that are administered by the service and remain part of the trial. Their assigned neuropsychiatrist can refer for psychological therapy outside of the service for any comorbid conditions, e.g. therapy for depression.

Training, supervision and fidelity checks

The study RA has received training to deliver screening interviews and collect data. The trial EMDR-trained psychological therapists have attended training on the FND-specific EMDR protocol, delivered by the Chief Investigator (SC). They receive regular clinical supervision from SC, as well as external supervision from an EMDR Consultant (a requirement of offering EMDR). Therapists will complete a session record form after every session, and these will be reviewed in supervision to enhance fidelity.

All sessions of EMDR will be video-recorded and the recordings stored on the secure SWLSTG NHS server. The therapists will share access to the recordings with SC for supervision purposes, and excerpts will be shown in EMDR supervision. Randomly selected recordings of processing sessions will be rated for fidelity, using the EMDR Fidelity Rating Scale Version 2 – Adverse Life Experiences Processing subscale, by an EMDR Consultant.[35] These scores will be analysed to evaluate fidelity.

Primary Objectives and Outcome Measures

A mixed methods approach will be used to establish feasibility and acceptability. The feasibility criteria and progression criteria are summarised in Table 3.

Table 3: Feasibility criteria and progression criteria for MODIFI

Criterion	Critical feasibility outcome	Other feasibility and acceptability data relevant to the criterion	Proposed threshold on critical outcome
Recruitment rate	% potentially eligible participants attending screening interview	Number of potentially eligible participants identified during neuropsychiatric assessment<br><br>Number of participants who consent and are randomised<br><br>Reasons for non-eligibility	*Above 70% attending a screening interview of those approached to participate, and if attendance is less than 70%, ways to increase the screening interview attendance rate will be considered.<br><br>**If 50-70% attend a screening interview of those approached to participate, and if attendance is less than 70%, ways to increase the screening interview attendance rate will be considered.<br><br>***If below 50% attend screening interview of those approached to participate, feasibility will not be demonstrated

For peer review only - http://bmjopen.bmj.com/site/about/guidelines.xhtml

Intervention adherence	% participants randomised to EMDR+NPC who complete therapy (Completion = attendance of 8 or more sessions, maximum session number =16)	Qualitative interviews with participants who attend EMDR<br><br>Therapy session record forms<br><br>Average number of sessions attended per course of therapy	*Feasibility will be demonstrated if above 70% complete therapy (attend 8 or more EMDR sessions)<br><br>**If 50-70% participants complete therapy, ways to improve engagement will be considered<br><br>***If less than 50% participants complete therapy, feasibility will not be demonstrated.
Outcome measurements completion	% participants who complete outcome measures at all time points	Retention of participants (rates of withdrawal across both arms)<br><br>Reasons for withdrawal<br><br>Qualitative interviews with participants	*Feasibility will be demonstrated if above 70% of participants complete outcome measures at each time point.<br><br>**If 50-70% of participants complete outcome measures at all time points, ways to improve retention and completion of outcome measures will be considered<br><br>***If less than 50% of participants complete outcome measures at all time points, feasibility will not be demonstrated

* = Continue to main study without modifications; ** = Future definitive trial is feasible with modifications; *** = Future definitive trial is not feasible.

Assessment of safety (adverse/serious adverse events) will be compared between the two arms. Therapy satisfaction and therapy fidelity will also be examined. The outcome measures used will be evaluated and the primary outcome measure identified, and the required sample size for a substantive RCT will be calculated.

The nested qualitative study will explore participants' and treating therapists' experiences and views. Information from these interviews will inform design and trial materials for a substantive study, including the FND-specific EMDR protocol. As the feasibility trial includes the option of attendance of appointments virtually, take up of this option will be measured.

Outcome measures

In terms of assessing FND, there is no single validated outcome measure for FND symptoms available.[36] Ecological Momentary Assessment (EMA) using the m-Path App will be used to assess FND symptoms. Participants will rate a maximum of two symptoms, chosen at the beginning of the trial period, e.g. seizures, tremor, limb weakness, tingling/numbness, gait disturbance, for a two-week period at each time point. For each of the two symptoms chosen, they will answer five questions daily (frequency, severity, interference, associated distress, associated preoccupation), and the mean for each item will be calculated for each two-week period at each time point.

The schedule of enrolment, interventions and assessments for participants is illustrated in Table 4. For descriptions of outcome measures, please see Supplementary Material.

For peer review only - http://bmjopen.bmj.com/site/about/guidelines.xhtml

Table 4: Schedule of enrolment, interventions and assessments for participants

	Screening	Baseline (T0)	3 months (T1)	6 Months (T2)	9 months (T3)	Post-Trial period
**Enrolment**						
**Consent to contact obtained by clinical staff (neuropsychistrist)**	x					
**Contacted by RA to arrange screening interview**	x					
**International Trauma Exposure Measure (ITEM)[37]**	x					
**Multiscale Dissociation Inventory (MDI)[38]**	x		x	x	x	
**Informed consent**	x					
**Demographics recorded**	x				x*	
**History of psychological therapy**		x				
**Current medication**		x				
**Randomisation allocation**		x				
**Interventions**						
**EMDR**		●——	———	———	——●	
**NPC**		●——	———	———	———	——●
**Assessments**						
**World Health Organisation Disability Assessment Schedule (WHODAS 2.0)[39]**		x	x	x	x	
**EQ-5F-5L[40]**		x	x	x	x	
**Ecological momentary assessment of FND symptoms via m-Path App**		x	x	x	x	
**PHQ-9[41]**		x	x	x	x	
**GAD-7[42]**		x	x	x	x	
**International Trauma Questionnaire (ITQ)[43]**		x	x	x	x	
**Adult Service Use Schedule (AD-SUS)[44]**		x			x	
**Beliefs related to diagnosis and intervention**		x			x	

For peer review only - http://bmjopen.bmj.com/site/about/guidelines.xhtml

**Clinical Global Impression - Improvement Scale (CGI-I)[45] rated by participant**				x	
**CGI-I rated by participant-nominated person**				x	
**Measure of satisfaction**				x	
**Review/reporting of patient AEs/SAEs**	x	x	x	x	
**Qualitative Interviews**					
**Informed consent for qualitative interview (select participants)**					x
**Qualitative interviews with select sample of participants and trial therapists**					x

* Participants will be asked at T3 (end of trial period) whether there have been any changes regarding medication, relationship status, and employment status and any changes recorded.

Recruitment and timeline for participants

Recruitment

Potential participants will initially be screened as part of routine neuropsychiatric appointments. The psychiatrist will verbally introduce the trial to potentially eligible participants and ask for permission for the RA to contact them. Potentially eligible participants' names will be passed to the CI, who will check that they likely meet the eligibility criteria. The RA will provide potential participants with a summary of the study and will send the Participant Information Sheet (PIS) and Informed Consent Form for them to review (see Supplementary Material). They will have the opportunity to ask any questions they may have. If willing to participate, the RA will obtain informed consent and arrange a screening interview.

The screening interview will be used to establish eligibility in terms of inclusion criterion (5) Reporting at least one traumatic event on the International Trauma Exposure Measure (ITEM), and exclusion criterion (7) Diagnosis of dissociative identity disorder or score in clinical range on "identity disturbance" subscale of Multiscale Dissociation inventory (MDI). [37,38] The (ITEM) will be used to assess previous adverse experiences. If they meet this inclusion criterion, potential participants will then complete the MDI, which will screen for clinical levels of dissociative "identity disturbance". Potential participants need to score in the non-clinical range of the subscale "Identity Disturbance" on the MDI to take part (score <15). Eligible participants will complete additional baseline measures, as well as completing demographic information, medical history, and listing any previous psychological therapies attended for any difficulty (not just FND).

The flow of participants is Illustrated in Figure 1.

<insert Figure 1>

Incentives

For peer review only - http://bmjopen.bmj.com/site/about/guidelines.xhtml

Participants will be reimbursed for research-related travel costs, up to the value of £20 per appointment (for those who choose to attend appointments in-person, rather than virtually). A non-contingent £25 incentive will be offered to participants for taking part 9 months after informed consent, unrelated to whether they complete the trial or not.

Allocation and Blinding

Consenting participants will be randomised into EMDR (plus NPC) or NPC in a 1:1 ratio. A stratified block randomisation (using randomly permuted blocks of sizes 2 and 4) will be used to ensure similar numbers of patients with and without PTSD symptoms (i.e., meeting or not meeting PTSD diagnostic criteria as determined by diagnostic algorithm of the International Trauma Questionnaire (ITQ)) are (relatively) equal across arms. Randomisations will be carried out by the Trial Manager (TM) using the randomisation function on REDCap.

Blinding

The trial is a single-blind trial with the RA and statistician remaining blind to treatment allocation. It is not possible to blind participants or treating clinicians to randomisation outcome.

Analysis and Statistical Methods

Sample size

This is a feasibility trial; as such, a power calculation is neither possible nor necessary. Rather, the sample size is pragmatic. Target recruitment is 50 patients in total (25 in each arm), which is consistent with those recommended for pilot and feasibility studies to provide sufficiently reliable estimates of feasibility outcomes, e.g. recruitment, adherence and attrition rates, and adequate precision of means and variances to inform a fully powered RCT.[46–48]

Statistical Analysis Plan

Data analysis will follow a statistical analysis plan (SAP), formally agreed with the trial steering committee prior to analysis, and centred on describing key process measures to decide if a definitive trial is feasible. Participant throughput will be summarised in an extended CONSORT diagram (Eldridge et al., 2016).

Feasibility outcomes will be summarised using descriptive statistics, with 95% confidence intervals (CIs) provided to permit assumptions when planning the main trial. Data relating to (serious) adverse events, assessment, screening, and recruitment logs will be used to produce accurate estimates of safety, eligibility, recruitment and consent rates in the study population. To determine the adequacy of study inclusion and exclusion criteria, and the generalisability of the trial to the FND population, baseline sociodemographic and clinical characteristics will be compared between study participants and ineligible and non-consenting patients. Intervention adherence (e.g. EMDR session attendance) and satisfaction of care data will be used to contribute to the evaluation of the acceptability of allocated intervention/treatment arms and mean EMDR fidelity scores for rated sessions calculated to assess intervention fidelity. At each time point, retention rates will be estimated for each of the patient reported/clinical outcome measures, with consideration given to differential dropout between the arms of the trial, to identify potential (attrition) bias in treatment completion and/or data collection. EMA

For peer review only - http://bmjopen.bmj.com/site/about/guidelines.xhtml

completion rates in each 2-week assessment period will be calculated with respect to daily assessment. All feasibility outcomes will be compared with relevant full-trial progression criteria.

Baseline characteristics will be reported according to treatment arm. Continuous variables will be reported as mean (standard deviation (SD)) if normally distributed or median (inter-quartile range) if non-normal, while categorical variables will be presented as frequency (%). Subsequent analyses will summarise the proposed patient-reported and clinical outcomes (e.g. quality of life and depression measures, (2-week mean) EMA symptom ratings) at each time point for each trial arm using appropriate descriptive statistics (e.g. group mean, SD). To provide an indication of potential changes in scores/frequencies between the four time points, linear/logistic mixed effects regression models will be employed performed on an intention-to-treat (ITT) basis (accounting for data assumed to be missing at random). These random intercept (mixed) models will include intervention group, time, and intervention group-by-time interaction. There will be no emphasis on hypothesis testing, however, which is reserved for the future main trial. Rather, pre-to-post-intervention standardised effect sizes (Hedges' g, relative risk) will be computed (SDs will be computed from estimated model standard errors) with associated CIs calculated to explore imprecision around effect sizes (Durlak, 2009). Due to the small sample size, important covariates (e.g. baseline score on relevant measure, gender, age) may be included in models if the two arms happen to be highly imbalanced. Additional analyses (using mixed effect models) focussed on 'per-protocol' outcomes and the potential value of the (intensively) collected EMA data on FND symptoms will also be administered (see Supplementary Material for additional detail).

A descriptive assessment of healthcare utilisation stratified by treatment arm will also be presented. The Adult Service Use Schedule (AD-SUS) will be used to record previous 6 months of health and social care resource use at baseline (T0) and 9 months of health and social care resource use at 9 months (T3). The acceptability of the AD-SUS will be assessed and key items of resource use for a future RCT will be identified. EQ-5D-5L utility scores will also be calculated. A cost effectiveness analysis will not be conducted.

Qualitative analysis plan

A sub-sample of participants in EMDR+NPC (n=8) and NPC (n=6) arms of the trial will be invited for in-depth semi-structured interviews after the intervention period. A sampling framework will be used that ensures participants are included that are representative of the sociodemographic characteristics and clinical profile (FS and FMD, presence of PTSD symptoms). Interviews will focus on the acceptability and feasibility of participating in a future larger trial of EMDR and explore experiences of recruitment practices, informed consent procedures, randomisation and range of outcomes measures. For participants in EMDR+NPC arm, interviews will also gauge the acceptability and perceived value of the EMDR intervention, suitability of number and frequency of sessions, ways of optimising engagement; and perceived benefits/limitations of the intervention as well as any recommendations for improvement. Final interview guides will be co-produced by the team, including PPI representatives.

Semi-structured interviews will also be carried out with both treating EMDR therapists. They will explore therapists' views concerning the research design and EMDR intervention protocol, including their experiences of training and delivering EMDR to patients with FND, as well as their perceptions of its deliverability within the NHS.

Interviews will be carried out by an RA, recorded and transcribed verbatim, with transcripts cross-checked against the original recordings to ensure accuracy. They will be analysed using reflexive thematic analysis.[49,50]

For peer review only - http://bmjopen.bmj.com/site/about/guidelines.xhtml

Trial Status

Enrolment of the first participant occurred on 19th December 2022. The trial is ongoing and we anticipate completing recruitment by November 2023.

Ethics and Dissemination

Research Ethical Approval

The research was reviewed by the NHS West Midlands - Edgbaston Research Ethics Committee with a favourable opinion (Reference: 22/WM/0178), and Health Research Authority approval has been received (both dated 27th September 2022).

Informed consent

Participants will provide informed consent prior to attending the screening appointment. This includes consenting to be recorded if randomised to EMDR+NPC. For the sub-sample of participants in the nested qualitative study, additional consent will be obtained at the point of being invited for interview. Please see Supplementary Material for additional detail.

Confidentiality and management of participant data

All data will be pseudo-anonymised and inputted on to the trial database, which will be saved on the secure NHS server. The Trial Master File will be backed up weekly on an encrypted hard drive. No paper copies will be stored. The data collection and management will be in line with GDPR Data Protection Act (2018). For details regarding data collection, data handling, and record keeping, refer to the Data Management Plan found in Supplementary Material.

Monitoring, Audit and Inspection

The trial will be monitored by the TMG and TSC. A Data Management Committee is not required as this is a feasibility study. The study will be self-monitored following a Monitoring Plan protocol. Please see Supplementary Material for detailed information.

Access to the final trial dataset

The CI and statistician will have access to the final trial dataset. If anyone else requires access, a request will need to be made via the TSC.

Post trial care

Participants who take part in the trial will have access to support from the Neuropsychiatry Service. Their care will be overseen by their allocated neuropsychiatrist and appropriate referrals made, if needed.

For peer review only - http://bmjopen.bmj.com/site/about/guidelines.xhtml

Dissemination

Trial findings will be published in a peer-reviewed journal or platform within 24 months from study completion. Authorship will be determined in accordance with the ICMJE guidelines and other contributors will be acknowledged. Participants will be notified via email after the results have been published. Trial Registries will be updated during the study and the trial protocol and key outcomes will be made publicly available within 12 months of study completion.

For peer review only

For peer review only - http://bmjopen.bmj.com/site/about/guidelines.xhtml

Contributors:

SC, MJE, JGS, SEL & SP provided significant input into the conception and design of the study. JGS completed the statistical analysis plan. JGS and SC drafted the qualitative analysis plan, and JB will supervise the qualitative aspects of the study. DG, PH, and KJT provided expert by experience input. SC trained the research assistant and trial therapists in reference to the trial protocols. SV and CP inputted into protocol modifications during the study set up phase. SC wrote the first draft of the manuscript. All authors reviewed the manuscript, and gave the final approval of the version to be published.

Sponsor:

South West London & St. George's Mental Health NHS Trust (SWLSTG)

R&D Office

Springfield University Hospital

1st Floor, Newton Building 7

61 Glenburnie Road, London SW17 7DJ

E: ResearchDevelopment@swlstg.nhs.uk

Competing interests:

SC - None declared; JS - None declared; SEL- None declared; SV – None declared; CP – None declared; SP – None declared; DG – None declared; PH – None declared; KT – None declared; JB - None declared; MJE – None declared

Funding:

This project is funded by the National Institute for Health and Care Research (NIHR) under its Research for Patient Benefit (RfPB) Programme (Grant Reference Number 202277). The views expressed are those of the author(s) and not necessarily those of the NIHR or the Department of Health and Social Care.

Sponsor roles and responsibilities

The Sponsor is responsible for providing the research team with adequate arrangements to successfully complete the trial, and to monitor its compliance with ethical guidelines and legislation. It is not involved in any aspect of the study design and management, or in its data collection, analysis or interpretation. SWLSTG Mental Health NHS Trust is the data controller for the study and all data shall return to the Sponsor at the end of the trial.

Funder roles and responsibilities

NIHR is not involved in any aspect of the study design and management, data collection, analysis, or interpretation. Core publications (Protocol and Results paper) were agreed with the NIHR before the start of the trial. NIHR will be acknowledged in any public dissemination of the research, including peer-reviewed articles, conference presentations and patient involvement activities.

For peer review only - http://bmjopen.bmj.com/site/about/guidelines.xhtml

Data availability statement:

Pseudoanonymised participant data is available upon reasonable request from the corresponding author Dr Sarah Cope (sarah.cope@swlstg.nhs.uk). Conditions for reuse will need to be discussed with the corresponding author on a case-by-case basis

Research Ethical Approval

The research was reviewed by the NHS West Midlands - Edgbaston Research Ethics Committee with a favourable opinion (Reference: 22/WM/0178), and Health Research Authority approval has been received (both dated 27th September 2022).

Acknowledgements:

The authors would like to thank the Trial Steering Committee (Dr Timothy Nicholson, Professor Richard J. Brown, Steve Portelly, Kirsty Griffin); staff working in the Neuropsychiatry Service (Dr Niruj Agrawal, Dr Norman Poole, Dr Andrea de Angelis, Dr Jacob Bird, Dr Gertrude Okello, Dr Charlotte Wattebot O'Brien, Dr Hendrik Hinrichsen, Dr Barnaby Proctor, Carla Vianello, Holly Zephrine, Rebecca Ewens, Cheryl Hamilton, Afsana Zaman) and St. George's Hospital (Dr Glenn Nielsen, Dr Shai Betteridge, Dr Jan Coebergh); and Maeve Crowley for her expert EMDR supervision.

For peer review only

References

1. Stone J, Carson A, Duncan R, et al. Who is referred to neurology clinics? - The diagnoses made in 3781 new patients. *Clin Neurol Neurosurg*. 2010;112(9):747-751. doi:10.1016/j.clineuro.2010.05.011

2. Carson A, Stone J, Hibberd C, et al. Disability, distress and unemployment in neurology outpatients with symptoms "unexplained by organic disease." *J Neurol Neurosurg Psychiatry*. 2011;82(7):810-813. doi:10.1136/jnnp.2010.220640

3. Magee JA, Burke T, Delanty N, Pender N, Fortune GM. The economic cost of nonepileptic attack disorder in Ireland. *Epilepsy & Behavior*. 2014;33:45-48.

4. Crimlisk HL, Bhatia KP, Cope H, David AS, Marsden D, Ron MA. Patterns of referral in patients with medically unexplained motor symptoms. *J Psychosom Res*. 2000;49(3):217-219. doi:10.1016/s0022-3999(00)00167-7

5. Healthcare Improvement Scotland. Stepped care for functional neurological symptoms. *Health Improvement Scotland*. 2012;(February). http://www.healthcareimprovementscotland.org/our_work/long_term_conditions/neurological_health_services/neurological_symptoms_report.aspx

6. Gilmour GS, Nielsen G, Teodoro T, et al. Management of functional neurological disorder. *J Neurol*. 2020;267(7):2164-2172. doi:10.1007/s00415-020-09772-w

7. Stone J, Carson A, Hallett M. Explanation as treatment for functional neurologic disorders. *Handb Clin Neurol*. 2016;139:543-553. doi:10.1016/B978-0-12-801772-2.00044-8

8. Nicholson C, Edwards MJ, Carson AJ, et al. Occupational therapy consensus recommendations for functional neurological disorder. *J Neurol Neurosurg Psychiatry*. 2020;91(10):1037-1045. doi:10.1136/jnnp-2019-322281

9. Nielsen G, Stone J, Matthews A, et al. Physiotherapy for functional motor disorders: A consensus recommendation. *J Neurol Neurosurg Psychiatry*. 2015;86(10):1113-1119. doi:10.1136/jnnp-2014-309255

10. Nielsen G, Buszewicz M, Stevenson F, et al. Randomised feasibility study of physiotherapy for patients with functional motor symptoms. *J Neurol Neurosurg Psychiatry*. 2017;88(6):484-490. doi:10.1136/jnnp-2016-314408

11. O'Connell N, Watson G, Grey C, Pastena R, McKeown K, David AS. Outpatient CBT for Motor Functional Neurological Disorder and Other Neuropsychiatric Conditions: A Retrospective Case Comparison. *J Neuropsychiatry Clin Neurosci*. 2020;32(1):58-66. doi:10.1176/appi.neuropsych.19030067

12. Goldstein LH, Robinson EJ, Mellers JDC, et al. Cognitive behavioural therapy for adults with dissociative seizures (CODES): a pragmatic, multicentre, randomised controlled trial. *Lancet Psychiatry*. 2020;7(6):491-505. doi:10.1016/S2215-0366(20)30128-0

13. Goldstein LH, Chalder T, Chigwedere C, et al. Cognitive-behavioral therapy for psychogenic nonepileptic seizures: A pilot RCT. *Neurology*. 2010;74(24):1986-1994. doi:10.1212/WNL.0b013e3181e39658

14. LaFrance WC, Baird GL, Barry JJ, et al. Multicenter pilot treatment trial for psychogenic nonepileptic seizures: A randomized clinical trial. *JAMA Psychiatry*. 2014;71(9):997-1005. doi:10.1001/jamapsychiatry.2014.817

For peer review only - http://bmjopen.bmj.com/site/about/guidelines.xhtml

15. Ludwig L, Pasman JA, Nicholson T, et al. Stressful life events and maltreatment in conversion (functional neurological) disorder: systematic review and meta-analysis of case-control studies. *Lancet Psychiatry*. 2018;5(4):307-320. doi:10.1016/S2215-0366(18)30051-8

16. Nicholson TR, Aybek S, Craig T, et al. Life events and escape in conversion disorder. *Psychol Med*. 2016;46(12):2617-2626. doi:10.1017/S0033291716000714

17. Paredes-Echeverri S, Guthrie AJ, Perez DL. Toward a possible trauma subtype of functional neurological disorder: Impact on symptom severity and physical health. *Front Psychiatry*. 2022;13. doi:10.3389/fpsyt.2022.1040911

18. Edwards MJ, Bhatia KP. Functional (psychogenic) movement disorders: Merging mind and brain. *Lancet Neurol*. 2012;11(3):250-260. doi:10.1016/S1474-4422(11)70310-6

19. Brown RJ, Reuber M. Towards an integrative theory of psychogenic non-epileptic seizures (PNES). *Clin Psychol Rev*. 2016;47:55-70. doi:10.1016/j.cpr.2016.06.003

20. Pick S, Goldstein LH, Perez DL, Nicholson TR. Emotional processing in functional neurological disorder: a review, biopsychosocial model and research agenda. *J Neurol Neurosurg Psychiatry*. 2019;90(6):704-711. doi:10.1136/jnnp-2018-319201

21. Lewis C, Roberts NP, Andrew M, Starling E, Bisson JI. Psychological therapies for post-traumatic stress disorder in adults: systematic review and meta-analysis. *Eur J Psychotraumatol*. 2020;11(1). doi:10.1080/20008198.2020.1729633

22. Shapiro F. The Role of Eye Movement Desensitization and Reprocessing (EMDR) Therapy in Medicine : Addressing the Psychological and Physical Symptoms Stemming from Adverse Life Experiences. 2014;18(1):71-77.

23. Tesarz J, Leisner S, Gerhardt A, Janke S, Seidler GH, Eich W. NEUROSCIENCE SECTION Review Article Effects of Eye Movement Desensitization and Reprocessing (EMDR) Treatment in Chronic Pain Patients : A Systematic Review. Published online 2014:247-263.

24. Rikkert M, van Rood Y, de Roos C, Ratter J, van den Hout M. A trauma-focused approach for patients with tinnitus: the effectiveness of eye movement desensitization and reprocessing–a multicentre pilot trial. *Eur J Psychotraumatol*. 2018;9(1). doi:10.1080/20008198.2018.1512248

25. Shapiro F. *Eye Movement Desensitization and Reprocessing (EMDR) Therapy: Basic Principles, Protocols, and Procedures*. Third Edition. The Guildford Press; 2018.

26. Wadji DL, Martin-Soelch C, Camos V. Can working memory account for EMDR efficacy in PTSD? *BMC Psychol*. 2022;10(1):245. doi:10.1186/s40359-022-00951-0

27. Chemali Z, Meadows ME. The use of eye movement desensitization and reprocessing in the treatment of psychogenic seizures. *Epilepsy and Behavior*. 2004;5(5):784-787. doi:10.1016/j.yebeh.2004.06.003

28. Kelley SDM, Benbadis S. Eye movement desensitization and reprocessing in the psychological treatment of trauma-based psychogenic non-epileptic seizures. *Clin Psychol Psychother*. 2007;14(2):135-144. doi:10.1002/cpp.525

29. Silver SM, Rogers S, Russell M. Eye movement desensitization and reprocessing (EMDR) in the treatment of war veterans. *J Clin Psychol*. 2008;64(8):947-957. doi:10.1002/jclp.20510

30. Cope S, Mountford L, Smith J, Agrawal N. Eye Movement Desensitisation and Reprocessing Therapy (EMDR) to treat functional neurological disorder: A review. *Journal of EMDR Practice and Research*. Published online 2018.

For peer review only

For peer review only - http://bmjopen.bmj.com/site/about/guidelines.xhtml

31. Cope SR. EMDR as an Adjunctive Psychological Therapy for Patients With Functional Neurological Disorder: Illustrative Case Examples. *Journal of EMDR Practice and Research*. Published online 2020:EMDR-D-20-00008. doi:10.1891/emdr-d-20-00008

32. Chan AW, Tetzlaff JM, Altman DG, et al. SPIRIT 2013 Statement: Defining Standard Protocol Items for Clinical Trials. *Ann Intern Med*. 2013;158(3):200. doi:10.7326/0003-4819-158-3-201302050-00583

33. National Institute for Health and Care Excellence. *Post-Traumatic Stress Disorder (NICE Guideline 116)*.; 2018.

34. Varese F, Sellwood W, Aseem S, et al. Eye movement desensitization and reprocessing therapy for psychosis (EMDRp): Protocol of a feasibility randomized controlled trial with early intervention service users. *Early Interv Psychiatry*. 2021;15(5):1224-1233. doi:10.1111/eip.13071

35. Korn DL, Maxfield L, Nancy Smyth CJ, Stickgold R. EMDR Fidelity Rating Scale (EFRS) The Manual. *EFRS Manual Version*. 1:2017-2025. http://emdrresearchfoundation.org/emdr-fidelity-

36. Pick S, Anderson D, Asadi-pooya AA, et al. Outcome measurement in functional neurological disorder: a systematic review and recommendations. *J Neurol Neurosurg Psychiatry*.

37. Hyland P, Karatzias T, Shevlin M, et al. Does requiring trauma exposure affect rates of ICD-11 PTSD and complex PTSD? Implications for DSM–5. *Psychol Trauma*. 2021;13(2):133-141. doi:10.1037/tra0000908

38. Brière J. MDI, Multiscale dissociation inventory: Professional manual. Published online 2002. Accessed August 31, 2018. https://scholar.google.co.uk/scholar?hl=en&as_sdt=0%2C5&q=briere+2002+MDI&oq=briere+2002+

39. World Health Organisation (WHO). *Measuring Health and Disability: Manual for WHO Disability Assessment Schedule (WHODAS 2.0)*. (Ustun T, Kostanjsek S, Chatterji JR, eds.).; 2010.

40. Herdman M, Gudex C, Lloyd A, et al. Development and preliminary testing of the new five-level version of EQ-5D (EQ-5D-5L). *Quality of Life Research*. 2011;20(10):1727-1736. doi:10.1007/s11136-011-9903-x

41. Kroenke K, Spitzer RL. The PHQ-9: A New Depression Diagnostic and Severity Measure. *Psychiatr Ann*. 2002;32(9):509-515. doi:10.3928/0048-5713-20020901-06

42. Spitzer RL, Kroenke K, Williams JBW, Löwe B. A Brief Measure for Assessing Generalized Anxiety Disorder. *Arch Intern Med*. 2006;166(10):1092. doi:10.1001/archinte.166.10.1092

43. Cloitre M, Shevlin M, Brewin CR, et al. The International Trauma Questionnaire: development of a self-report measure of ICD-11 PTSD and complex PTSD. *Acta Psychiatr Scand*. 2018;138(6):536-546. doi:10.1111/acps.12956

44. Tyrer P, Cooper S, Salkovskis P, et al. Clinical and cost-effectiveness of cognitive behaviour therapy for health anxiety in medical patients: a multicentre randomised controlled trial. *Lancet*. 2014;383(9913):219-225. doi:10.1016/S0140-6736(13)61905-4

45. Guy W. Clinical Global Impressions (CGI) Scale, Modified. In: Rush JA, ed. *Handbook of Psychiatric Measures*. 1st Edition. American Psychiatric Association; 2000.

46. Sim J, Lewis M. The size of a pilot study for a clinical trial should be calculated in relation to considerations of precision and efficiency. *J Clin Epidemiol*. 2012;65(3):301-308. doi:10.1016/j.jclinepi.2011.07.011

For peer review only - http://bmjopen.bmj.com/site/about/guidelines.xhtml

47. Lancaster GA, Dodd S, Williamson PR. Design and analysis of pilot studies: recommendations for good practice. *J Eval Clin Pract*. 2004;10(2):307-312. doi:10.1111/j..2002.384.doc.x

48. Julious SA. Sample size of 12 per group rule of thumb for a pilot study. *Pharm Stat*. 2005;4(4):287-291. doi:10.1002/pst.185

49. Braun V, Clarke V. Using thematic analysis in psychology. *Qual Res Psychol*. 2006;3(2):77-101. doi:10.1191/1478088706qp063oa

50. Braun V, Clarke V. Reflecting on reflexive thematic analysis. *Qual Res Sport Exerc Health*. 2019;11(4):589-597. doi:10.1080/2159676X.2019.1628806

Figure 1: CONSORT flow diagram for MODIFI study

For peer review only

For peer review only - http://bmjopen.bmj.com/site/about/guidelines.xhtml

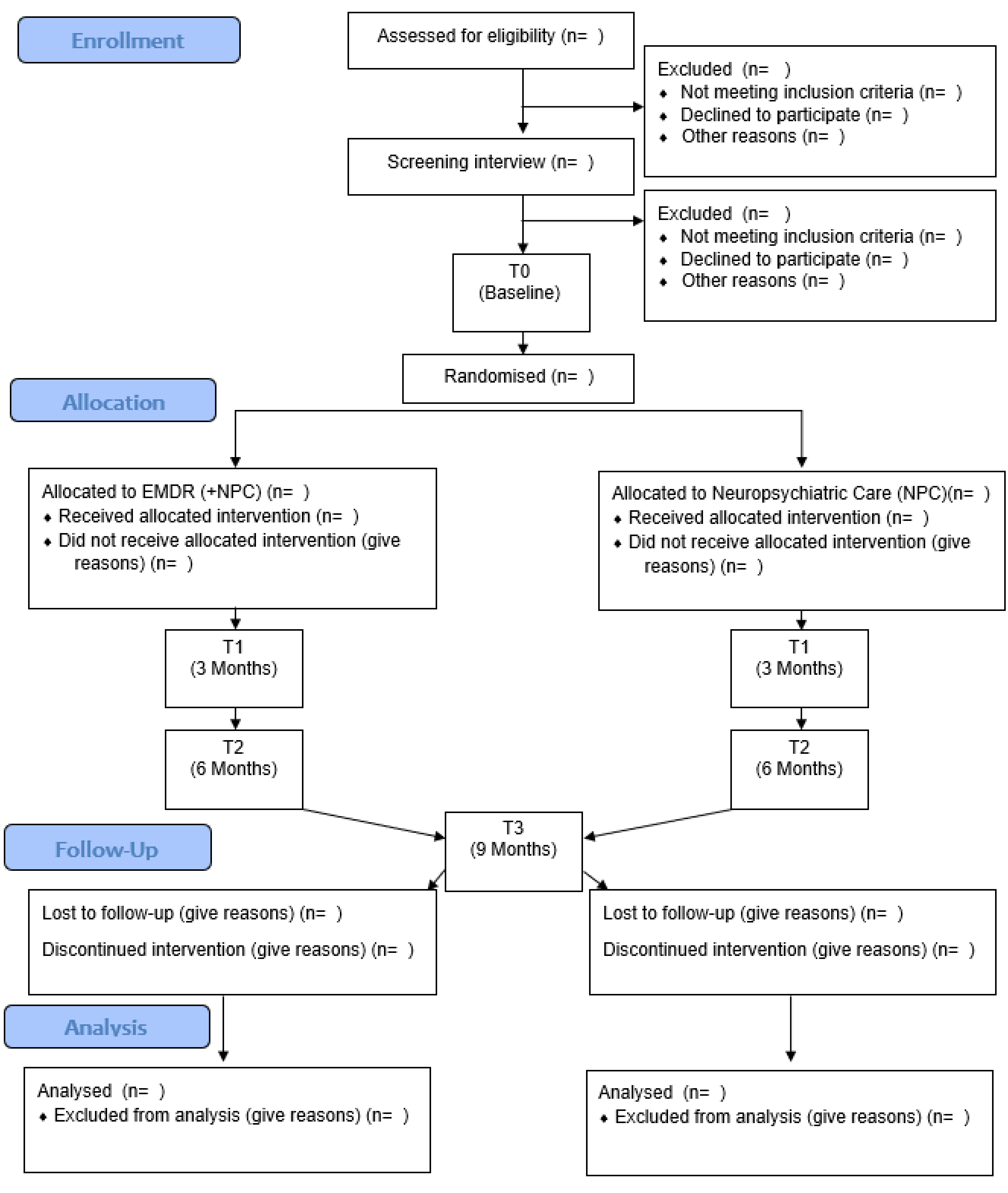
Enrollment
Assessed for eligibility (n=)
Excluded (n=)
Not meeting inclusion criteria (n=)
Declined to participate (n=)
Other reasons (n=)
Screening interview (n=)
Excluded (n=)
Not meeting inclusion criteria (n=)
Declined to participate (n=)
Other reasons (n=)
T0
(Baseline)
Randomised (n=)
Allocation
Allocated to EMDR (+NPC) (n=)
Received allocated intervention (n=)
Did not receive allocated intervention (give reasons) (n=)
Allocated to Neuropsychiatric Care (NPC)(n=)
Received allocated intervention (n=)
Did not receive allocated intervention (give reasons) (n=)
T1
(3 Months)
T1
(3 Months)
T2
(6 Months)
T2
(6 Months)
Follow-Up
T3
(9 Months)
Lost to follow-up (give reasons) (n=)
Discontinued intervention (give reasons) (n=)
Lost to follow-up (give reasons) (n=)
Discontinued intervention (give reasons) (n=)
Analysis
Analysed (n=)
Excluded from analysis (give reasons) (n=)
Analysed (n=)
Excluded from analysis (give reasons) (n=)

MODIFI Protocol – Supplementary Material

Contents

Outcome Measures 2
Additional details regarding Consent, Blinding, and Confidentiality 5
Additional details regarding Analysis and Statistical Methods 8
Recording and Reporting of Events 10
Monitoring, Audit & Inspection 12
Data Management Plan (DMP) 15
Participant Information Sheet
Participant Consent Form

For peer review only

For peer review only - http://bmjopen.bmj.com/site/about/guidelines.xhtml

Outcome Measures

I. Health-Related Quality of Life/Functioning:

World Health Organisation Disability Assessment Schedule (WHODAS 2.0) (WHO, 2010) is a 36-item generic assessment instrument for health and disability. The questionnaire asks about difficulties due to health conditions and asks participants to rate these on a scale with 5 levels: None, Mild, Moderate, Severe, Extreme or cannot do.

EQ-5D-5L (Herdman et al., 2011) consists of a descriptive system assessing five dimensions: mobility, self-care, usual activities, pain/discomfort and anxiety/depression. Each dimension has 5 levels: no problems, slight problems, moderate problems, severe problems and extreme problems. It also has a self-rated health (0-100) visual analogue scale, where the endpoints are labelled 'The worst health you can imagine' (0) and 'The best health you can imagine' (100).

II. FND:

There is no single validated outcome measure for FND symptoms available (Pick et al., 2020). Ecological Momentary Assessment (EMA) using the m-Path App, which is well supported and maintained across all smartphone platforms, will be used to assess FND symptoms.

Participants will rate a maximum of 2 symptoms, chosen at the beginning of the trial period, e.g. seizures, tremor, limb weakness, tingling/numbness, and gait disturbance. A paper version will be available to participants if necessary. Participants will be encouraged to choose the 1 or 2 symptom(s) that currently have the worst impact on their quality of life.

Each participant would answer the following questions once a day over each 2-week time period for a maximum of two symptoms:

Select the symptom you are referring to [seizures, tremor, limb weakness, tingling/numbness, jerks, gait disturbance]. With this symptom in mind, please rate the following:

a. If the symptom is episodic (not present all the time), rate the number of times (frequency) it has occurred in the previous 24 hours, or answer not applicable (n/a) if your symptom is continuous

b. Over the last 24 hours, please rate the severity of the symptom, from 0 (none) – 10 (extremely severe). If the symptom has not occurred, rate 0 for this question.

c. Over the last 24 hours, please rate the extent the symptom has interfered with your daily functioning, from 0 (not at all) – 10 (extreme interference)

d. Over the last 24 hours, please rate the extent the symptom has distressed you, from scale 0 (none) – 10 (extreme distress)

e. Over the last 24 hours, please rate the extent the symptom has preoccupied you (been on your mind), from 0 (not at all) – 10 (all of the time)

For each of the 2 symptoms chosen, the mean for each item will be calculated for each 2-week period at each time point.

For peer review only - http://bmjopen.bmj.com/site/about/guidelines.xhtml

III. Depression and anxiety:

PHQ-9 (Kroenke & Spitzer, 2002) is a 9-item measure assessing presence and severity of depression. Each item is rated on a 4-point scale ranging from 'Not at all' to 'Nearly every day'.

GAD-7 (Spitzer et al., 2006) is a 7-item measure assessing presence and severity of anxiety. Each item is rated on a 4-point scale ranging from 'Not at all' to 'Nearly every day'.

IV. PTSD:

International Trauma Exposure Measure (ITEM) (Hyland et al., 2021) is a checklist developed to capture traumatic life events, and their associated features, in a manner consistent with the description of a trauma in the ICD-11 diagnostic manual. It assesses exposure to different traumatic life events across different developmental periods (childhood, adolescence, adulthood, and lifetime); frequency of exposure to one's most distressing traumatic event; and the main emotion associated with one's most distressing traumatic event.

International Trauma Questionnaire (ITQ) (Cloitre et al., 2018) is a valid and reliable measure that assesses PTSD and Complex PTSD in line with the ICD-11.

V. Dissociation:

Multiscale Dissociation Inventory (MDI) (Briere, 2002) is a 30-item self-report measure of dissociative experiences in the previous month. It measures six different types of dissociation: disengagement, depersonalisation, derealisation, emotional constriction, memory disturbance, and identity dissociation.

VI. Service utilisation and other cost variables:

Adult Service Use Schedule (AD-SUS) (Tyrer et al., 2014). The AD-SUS was developed by an economist for previous work in similar populations and was adapted for the needs of this evaluation. It assesses employment, healthcare utilisation and medication.

VIII. Improvement:

Clinical Global Impression – Improvement Scale (CGI-I) rated by participant (single item, 7-point scale) (Guy, 2000)

CGI-I rated by person nominated by participant (e.g. family member, partner, close friend, carer) (single item, 7-point scale)

IX. Beliefs about diagnosis and intervention:

Agreement with diagnosis of FND (single item, 11-point scale)

For peer review only - http://bmjopen.bmj.com/site/about/guidelines.xhtml

Preference regarding treatment (EMDR + NPC, NPC or no preference)

Belief in having been given the right treatment (single item, 11-point scale)

X. Measure of satisfaction

Satisfaction rating of treatment (single-item, 11-point scale)

For peer review only

For peer review only - http://bmjopen.bmj.com/site/about/guidelines.xhtml

Additional details regarding Consent, Blinding, and Confidentiality

The RA will assess risk and capacity to provide consent throughout the screening and assessment procedures, with input from the Chief Investigator, who is a clinical psychologist. The limits of confidentiality will be made clear to participants. There will be a standard protocol for assessing and managing safeguarding and/or risk disclosures, which will follow safeguarding relevant policies and procedures of SWLSTG Mental Health NHS Trust.

Those not eligible to take part in the research due to not endorsing experiencing any adverse events or experiencing high identity disturbance, and who are interested in the option of attending psychological therapy, will be offered an appointment with the CI (who is also the team's lead clinical psychologist) to discuss treatment options local to them or within the service. This will mean if non-eligible participants are distressed by non-inclusion, the RA will be able to offer them an appointment with a clinical member of staff.

Anonymised information on participants who are not randomised will include demographic data, diagnoses, type and duration of FND symptoms, the reason not eligible for trial participation, or if they are eligible but declined.

Consent

Participants will consent by one of the following:

Face to face with the RA. The participant and RA will sign the consent form. The RA will scan the original and save a copy in the electronic site file, upload a copy to the patient's electronic notes, and give a copy of the form to the participant. The paper form will be shredded.

Remote consent, whereby the consent form is shared electronically and the participant signs electronically and returns the form to the RA, who will then sign the form themselves and send a fully signed copy to the participant. The RA will file the original in the Trial Master File (TMF) and upload a copy to the patient's electronic notes.

The RA who will be responsible for taking written informed consent will have received training in Good Clinical Practice (GCP) alongside internal additional training by the study team.

Additional consent provisions for collection and use of participant data

All trial participants will be asked whether they would be willing to be contacted at a later stage for participating in further studies related to the trial. This consent to contact will include the qualitative interview. It will be made clear that this is optional and that declining consent to be contacted for participation in additional studies will not prevent them from taking part in the trial.

For the qualitative interview, it will also be made clear that not everyone will be contacted, since we will only be recruiting a sub-sample. If a participant indicates willingness, the RA will contact them directly once all participants have completed the study period, and all

For peer review only - http://bmjopen.bmj.com/site/about/guidelines.xhtml

quantitative data has been collected. Written consent will be obtained from those consenting to interview, and the form will be stored as above.

EMDR Therapists will also provide written informed consent to participate in interviews. The signed form will be filed in the site file, and a copy of the form will be given to the therapist.

Additional details regarding blinding

Participants will be reminded, when the RA meets with them to complete measures, that the RA must not be told which arm they have been randomised to. The TM will input which arm (Group 1 or Group 2) each participant was allocated to after all participants have completed their time in the study and the data has been inputted, but they will not disclose whether Group 1/2 refers to EMDR+NPC or NPC, so that the statistician will remain blind to treatment allocation whilst carrying out data analysis.

Long-term follow-up

As part of informed consent, participants will be asked to consent to open-label follow-up to potentially provide additional data for a prospective substantive RCT.

Withdrawal criteria

It will be made clear to participants that they have the right to withdraw from the study at any time for any reason, without the need to justify their decision, and that it will not affect their routine care. The investigator also has the right to withdraw participants from the study in the event of clinical contra-indications. Should a participant withdraw from therapy only but not from the study, efforts will be made to continue to obtain follow-up data, with the permission of the participant. Should a participant withdraw from the study, we will still use any previous data collected from that participant up to the point of withdrawal, but will make no further attempt to contact or collect data

Data protection and patient confidentiality

Clinical confidentiality

During the screening interview, potential participants will be advised of the limits of confidentiality (i.e. that the researcher will have a duty to inform health professionals if the participant discloses information any safeguarding or risk issues). It is also possible that disclosure of significant criminal acts potentially requiring action will occur during assessment and therapy sessions. The research team will follow the protocol for Assessing and Managing Safeguarding and Risk Disclosures. The RA and trial therapists will seek supervision and ensure appropriate action is taken as soon as possible. The limits of confidentiality and possibility of action arising from certain disclosures will be noted in the Participant Information Sheet (PIS). Potential participants will be offered at least 24 hours to consider all the information provided before written consent is obtained. Therapists will discuss the limits of confidentiality with participants allocated to the EMDR+NPC at the start of therapy, and at any appropriate subsequent points during the therapy.

For peer review only - http://bmjopen.bmj.com/site/about/guidelines.xhtml

Data confidentiality

Research data will be confidential unless a participant discloses information that indicates that they or another person are at risk of harm (see clinical confidentiality section above). All participants will be informed of this during the written informed consent process.

All data will be pseudonymised. Each participant will be assigned a unique participant number when they are referred to the trial, which will remain the same for the duration of their involvement in the trial. This number will be recorded on all eligibility measures, forms and databases used to record data on participants. An electronic record sheet linking participant identity, contact details, and trial identification number will be created and saved on the secure Trust drive. The password will only be shared with the research team. All data will be kept secure at all times and maintained in accordance with General Data Protection Regulation (GDPR, 2018) requirements and archived according to clinical trial GCP regulations. Participant consent forms will be retained electronically, kept confidential and stored securely.

Only the RA, TM and chief investigator will have access to the trial database. The statistician will receive a copy of the database once data collection is complete and the database is locked.

Therapy notes will be recorded on Rio, the electronic clinical record system used by SWLSTG, in line with Trust procedures. The RA will access the system when a participant referral is received, but will not need to access the system thereafter, so will not view participants' clinical records after recruitment to the study.

Recordings

All therapy sessions (with participant consent) will be recorded to monitor the fidelity of the intervention delivery. When sessions are held online via MS Teams, recording will take place via the platform. When sessions are held in-person, clinicians will record each session by using MS Teams too (they will position their laptop so both participant and clinician can be viewed). Each recording file will be named with participant identifier and date, and will be stored on the secure SWLSTG NHS server. Recordings of the therapy will be accessible to the participant's therapist, the CI, and excerpts will be shown in clinical supervision. A random selection of processing sessions will be shown to an EMDR Consultant in order to rate the sessions for fidelity. The video recordings are only being used to enhance fidelity to the therapy protocol, and they will be deleted after a random selection of them has been rated for fidelity.

Interviews will be held remotely via MS Teams or in-person. Recordings will take place via MS Teams in both instances with the video recording function turned off (only audio will be captured). All recordings will be labelled and stored securely on the Trust server. All audio recordings will be deleted after transcription has been completed. The transcriptions will be stored in the TMF, and stored for a minimum of 10 years in line with the other essential data.

For peer review only - http://bmjopen.bmj.com/site/about/guidelines.xhtml

Additional details regarding Analysis and Statistical Methods

Per-protocol analyses of outcome measures

Exploratory analyses using mixed effect models will examine the rate of change in intervention and control groups on outcome measures across four time-points, adjusting for relevant baseline scores and variables of interest (predominant symptom, presence of PTSD, levels of dissociation), and investigate changes on a per-protocol basis (focused on intervention adherence; i.e. including only participants who attended at least 8 sessions and with post-treatment data).

EMA data

The potential value of the (intensively) collected EMA data on FND symptoms will also be considered. EMA completion rates in each 2-week assessment period will be calculated with respect to daily assessment (total days with a completed assessment divided by total number of scheduled assessments); rates will be calculated for all assessments combined and for each assessment separately (symptom frequency, severity, and symptom-associated interference, preoccupation and distress) and the proportion of participants completing at least 33% of the total number of EMAs will be recorded.

Within participant day-to-day variability of repeated FND symptom ratings (e.g., frequency, severity) and Cronbach's alphas for EMA items (symptom severity, interference, distress, preoccupation) will be calculated at each time point to explore reliability and internal consistency of the EMA App, while exploratory mixed effect modelling analyses will adopt a three-level structure (repeated measurements at each EMA in addition to time point and participant). To explore relationships of EMA App data with other clinical measures, bivariate correlations (Pearson r or Spearman rho, according to data distribution) will be completed between 2-week mean EMA symptom ratings and corresponding WHODAS scores and between change in 2-week mean EMA symptom ratings and patient and clinician-rated CGI-I scales/change in WHODAS across corresponding time points. These analyses are expected to inform the analytic plan for a future larger study.

Procedure(s) to account for missing or spurious data

All available data will be included in the data listings and tabulations. If data exhibit missing or spurious values, aspects related to their collection, recording, and analysis will be investigated. The frequency (percentage) of participants with missing outcome values at each time point (retention rates), in addition to the reasons for missingness (where available), will be summarised. For (continuous/categorical) secondary outcomes, intention to treat (ITT) (linear/logistic) mixed effect models with maximum likelihood estimation will examine the rate of change in intervention and control groups on outcome measures across three post-treatment time-points, accounting for data missing at random. Given the feasibility status of the study, a full sensitivity analysis testing various missing data assumptions is not viable. Nevertheless, we will administer additional analyses imputing values for missing data using a conservative last observation carried forward (LOCF) procedure (in these analyses, where data is missing from assessment, a first observation carried backwards (FOCB) procedure will also be adopted). Safety and adverse/unwanted effects will be reported on an

For peer review only - http://bmjopen.bmj.com/site/about/guidelines.xhtml

as-treated basis (as opposed to an ITT approach), as these are best considered using the most accurate information.

For peer review only

For peer review only - http://bmjopen.bmj.com/site/about/guidelines.xhtml

Recording and Reporting of Events

The recording and reporting of AEs will follow the “Recording and reporting of (S)AEs protocol”.

Operational definitions for AEs and SAEs

Adverse Events (AEs) are defined by the Health Research Authority (HRA) as any untoward medical occurrence, unintended disease or injury, or untoward clinical signs in participants, whether or not related to the treatment. In addition, issues specific to psychological therapies will also be monitored, namely: clinically significant increases in mental health or physical health problems; risky or problematic behaviours; harm to self/others, including suicide attempts; harm from others; and emergency room visits or crises. Clinically significant increases will be operationalised as an unresolved exacerbation requiring increased involvement from the care team, e.g. alterations to treatment plan. Distress or complaints associated with therapy, completion of assessment measures, or any other trial procedure would also constitute AEs.

The causes for the AEs will also be recorded and monitored. For each AE, the following potential reasons will be identified:

- Victimisation: aggressive behaviour, sexual abuse/assault, physical abuse/assault, emotional abuse/psychological maltreatment, exploitation, and other victimisation
- Mental health/psychological difficulties: excessive use of substances, exacerbation of mental health difficulties, suicidal ideation, and other psychological difficulties
- Trial procedures: group allocation, assessments, or therapy
- Physical health, including exacerbation of presenting FND symptoms, new neurological or other physical symptoms, and COVID-19 infection.
- Accidents or natural disasters
- Other.

AEs will be initially assessed at three levels of severity; mild, moderate and severe, which reflect the impact of the event on the person at the time. Please note there is a distinction between “severe” and “serious”. Seriousness is the criteria for defining regulatory reporting obligations:

Serious Adverse Events (SAEs) are defined as:

- Death and life-threatening events (Category A)
- Incidents which acutely jeopardise the health or psychological wellbeing of the individual, resulting in immediate hospital admission and/or persistent or significant disability or incapacity (category B)
- Resulting in injury requiring immediate medical attention (category C).

For peer review only - http://bmjopen.bmj.com/site/about/guidelines.xhtml

All AEs and SAEs will be reported to the TM and CI. A summary of (S)AEs will be presented at each Trial Management Group (TMG) and Trial Steering Committee (TSC) meeting. Urgent actions concerning participant and staff safety, communication with others, and clinical care will be immediately addressed by the CI and reported to the TMG.

AEs will be categorised for severity and seriousness by the TM and CI. SAEs will be further reviewed for relatedness to trial procedures and unexpectedness by the CI initially, and additionally by the chair of the TSC.

Relatedness and unexpectedness of an event to the intervention will be judged based on the following:

1. Related: the event resulted from administration of any of the research procedures, judged according to a temporal relationship (i.e., Serious Related Events; SREs);
2. Unexpected: the event is unexpected or unexplained given the participant's clinical course, previous conditions and history, and concomitant treatments (i.e., Unexpected Serious Related Events; USREs)

Recording and reporting of (S)AEs

Best practice, professional guideline and local NHS policies for monitoring mental state and risk will be followed throughout the participants' involvement in the trial and will be facilitated by the trial being embedded in the Neuropsychiatry Service. The safety of the intervention will be monitored closely during therapy sessions and through contacts with the Neuropsychiatry Service.

The occurrence of AEs will be monitored actively and systematically and recorded by the RA, therapists, and clinical team. All AEs and participant withdrawals will be recorded and monitored by the trial team. If indicated, the TM and CI will review the clinical notes and contact clinicians for any important additional information. At the completion of the trial, all clinical notes will additionally be checked, for the total duration of enrolment, for any previously undisclosed record of AEs. This extra procedure is to ensure completeness of records and to address the possibility of an increased likelihood of disclosure of AEs in the EMDR+NPC condition, as a result of greater frequency of contact and the therapeutic relationship. In the final reports of the trial, the numbers, types and severity of AEs by trial condition, as well as discontinuations, will be reported.

Notification of deaths

All deaths will be reported to the sponsor within one month regardless of whether the death is related to the trial, disease or an unrelated event.

For peer review only - http://bmjopen.bmj.com/site/about/guidelines.xhtml

Monitoring, Audit & Inspection

A trial manager is in post to coordinate the day-to-day running of the trial.

Trial Management Group (TMG):

Dr Sarah Cope – Chief investigator

Prof. Mark Edwards – Co-investigator

Dr Sharif El-Leithy – Co-investigator

Dr Jared Smith – Co-investigator

Dawn Golder – PPI representative

Dr Patricia Hogwood – PPI representative

Kati Jane Turner – PPI representative

Dr Serena Vanzan – Trial Manager

Caitlin Pentland – Research Assistant

A Sponsor representative will observe each meeting.

TMG Terms of Reference

1. To review and provide feedback on the study protocol, outcome measures and recruitment documents (including any amendments)
2. To support the progress of the trial towards its interim and overall objectives and against the study timescale
3. To agree on a trial monitoring plan
4. To advise on participant recruitment and retention
5. To facilitate data analysis
6. To support the troubleshooting of any hindrances to the timely completion of the trial
7. To review the study outcomes
8. To discuss future developments of the study
9. To report to the Trial Steering Committee (TSC)

Trial Steering Committee (TSC)

The TSC will oversee the study on behalf of the of the trial Sponsor and Funder and ensure that the study is conducted within appropriate NHS and professional ethical guidelines. It will provide advice on all appropriate aspects of the project; will oversee progress of the trial, adherence to the protocol, participant safety and the consideration of new information of relevance to the research question; will ensure the rights, safety and well-being of the

For peer review only - http://bmjopen.bmj.com/site/about/guidelines.xhtml

participants are given the most important considerations; will ensure appropriate ethical and other approvals are obtained in line with the project plan; will agree proposals for substantial protocol amendments and provide advice to the Sponsor and Funder regarding approvals of such amendments.

The TSC comprises of:

Dr Tim Nicholson– Independent Chairman

Prof Richard Brown – Independent Expert

Dr Sarah Cope – Chief investigator

Dr Serena Vanzan – Trial Manager

Steve Portelly – Independent PPI representative

Kirsty Griffin – Independent PPI representative

A Sponsor representative will observe each TSC meeting.

TSC Terms of Reference

1. To review and approve the study protocol and recruitment documents (including any amendments)
2. To monitor and supervise the progress of the trial towards its interim and overall objectives and against the study timescale
3. To ensure all trial activities are conducted in line with GCP guidelines
4. To inform the funder annually on the progress of the trial
5. To advise the funder on publicity and the presentation of all aspects of the trial

Protocol compliance

A study related deviation is a departure from the ethically approved study protocol or other study document or process (e.g. consent process or administration of study intervention) or from Good Clinical Practice (GCP) or any applicable regulatory requirements. Any deviations from the protocol will be documented in a protocol deviation form and filed in the study master file.

The reporting procedure for any potential protocol breaches will be reported to the CI and sponsor. Any potential breach will be assessed by the CI and their supervisor(s), and reported as below where this may be a potential serious breach.

Notification of Serious Breaches to GCP and/or the protocol

A “serious breach” is a breach of the protocol or of the conditions or principles of Good Clinical Practice which is likely to affect to a significant degree: (a) the safety or physical or mental integrity of the trial subjects; or (b) the scientific value of the research.

For peer review only - http://bmjopen.bmj.com/site/about/guidelines.xhtml

In the event that a serious breach is suspected, the Sponsor will be contacted within 1 working day. In collaboration with the CI, the serious breach will be reviewed by the Sponsor and, if appropriate, the Sponsor will report it to the approving REC committee and the relevant NHS host organisation within seven calendar days.

Amendments

In the event of the sponsor wishing to make substantial amendments to the REC application or any supporting documentation, they will submit a valid notice of amendment to the REC for consideration.

The SWLSTG NHS and R&D departments will be notified.

The sponsor in collaboration with CI and investigators who contributed to the protocol will determine whether an amendment to the protocol is required and is substantial.

The CI will seek guidance from the local REC office. Changes that are deemed to be substantial will be communicated to the REC and SWLSTG NHS R&D department via the notification system in IRAS.

Any amendments will constitute a new approved version and will have a new label and date in order to distinguish it from the past protocol.

For peer review only

Data Management Plan (DMP)

Randomised feasibility study of eye movement desensitisation and reprocessing therapy (EMDR) for functional neurological disorder (FND) (MODIFI)
COPS1001

DMP SIGN-OFF SHEET

Trial Forms	Version Number	Version Date
MODIFI_Research protocol_v3.1_23Nov2022	3.1	23/11/2022
MODIFI_Participant invite letter_v1.2_23Nov2022	1.1	23/11/2022
MODIFI_Consent Form_v1.3_23Nov2022	1.3	23/11/2022
MODIFI_PIS_v3.1_23Nov2022	3.1	23/11/2022
MODIFI_GP letter_v2.0_07Sep2022	2.0	07/09/2022

I have reviewed the trial's DMP and approved the use of the above documents

Name	Position	Signature	Date
Sarah Cope	Chief Investigator		04/11/2022
Serena Vanzan	Trial Manager		03/11/2022
Caitlin Pentland	Research Assistant	Caitlin Pentland	09/11/2022
Jared Smith	Trial Statistician		31/01/2023

Comments

For peer review only - http://bmjopen.bmj.com/site/about/guidelines.xhtml

Randomised feasibility study of eye movement desensitisation and reprocessing therapy (EMDR) for functional neurological disorder (FND) (MODIFI)

COPS1001

1. Trial information

Trial type	Non-CTIPM Intervention - Feasibility
Study Sites	Single-site (SWLSTG)
Total sample size	50
Total duration of study (months)	Trial start date _01/06/2022__<br>Planned duration for recruitment (months)___12____<br>Planned duration of follow-up (months)___9____<br>Total Duration ___36____Months
Study objective and design	The study aims to evaluate the possibility of delivering, and potential benefit, of EMDR for FND.<br>We will recruit 50 participants who have specific functional neurological symptoms: weakness, walking difficulties, jerks, shaking, and/or seizures from a Neuropsychiatry Service. Participants will be randomly allocated to EMDR, and routine medical appointments, or routine medical appointments alone. Those allocated to EMDR will be offered 8-16 weekly therapy sessions, completed within 6 months, and a follow-up session 1 month after therapy has ended. Participants will be able to choose whether they attend therapy in-person or via an online video conferencing platform. Participants will complete questionnaires regarding their health-related functioning, FND, mental health, and healthcare utilisation. These questionnaires will be completed at the beginning, and at 3 months, 6 months, and 9 months.<br>Some participants will attend interviews about their experiences of treatment.
Primary outcome measure	This is a feasibility study so no primary outcome measure has been chosen, as one of the study's aims is to investigate the value of a range of outcome measures, to determine the outcome measure with greatest symptom improvement and the required sample size, for a

For peer review only - http://bmjopen.bmj.com/site/about/guidelines.xhtml

	substantive RCT.
Secondary outcome measures	Health-Related Quality of Life/Functioning<br>FND<br>Depression and anxiety<br>PTSD<br>Dissociation<br>Service utilisation and other cost variables<br>Improvement<br>Beliefs about diagnosis and intervention<br>Measure of satisfaction
Interim analysis	Yes/**no**, if yes when____________(Months from baseline)

2. Trial personnel and contact details

This section details the name, their position in the trial, email address, telephone/fax number for all staff involved in the trial including the sponsor. The trial coordinator/trial manager, the investigators, study staff involved in the data management (including computing staff responsibilities for maintaining hardware and software), the monitors and anyone else associated with the trial at each site.

2.1 Sponsor site personnel (add or remove accordingly)

Role	Name	Organisation	Contact Details
Sponsor	SWLSTG R&D	SWLSTG	ResearchDevelopment@swlstg.nhs.uk
Chief Investigator	Sarah Cope	SWLSTG	Sarah.Cope@swlstg.nhs.uk
Trial Manager	Serena Vanzan	SWLSTG	Serena.Vanzan@swlstg.nhs.uk
Research Assistant	Caitlin Pentland	SWLSTG	Caitlin.Pentland@swlstg.nhs.uk
Co-Investigator and Trial statistician	Jared Smith	SGUL	jasmith@sgul.ac.uk
Co-Investigator	Sharif El Leithy	SWLSTG	Sharif.Elleithy@swlstg.nhs.uk
Co-Investigator	Mark Edwards	KCL	mark.j.edwards@kcl.ac.uk
Primary contact for DM issues	Serena Vanzan	SWLSTG	Serena.Vanzan@swlstg.nhs.uk
Secondary contact for DM issues	Sarah Cope	SWLSTG	Sarah.Cope@swlstg.nhs.uk

For peer review only - http://bmjopen.bmj.com/site/about/guidelines.xhtml

3. Milestones

3.1 Study Milestones

Milestone	Date/Estimated Date
Date funding confirmed	22.03.2021
Date and version number of approved protocol	05.10.2022, v3.0
Date and version number of final protocol amendment(s)	03.11.2022, Am01
Date/version number of final approved CRF	No approval required
Release date/version number of final database	01.12.2022
Date DMP signed off	31.01.2023
Date of first participant first visit (FPFV)	19.12.2022
Date last participant last visit (LPLV)	

3.2 Proposed Data Milestones

Milestone	Date/Estimated Date
Data entry to commence	w/s 07.11.2022
Date of interim data partial lock if applicable	n/a
Data entry completed	Est Jun 2024
Last query resolved in the system	Est Aug 2024
Date of database final lock	Est Aug 2024

4. Data collection & data entry system

4.1 *Detail how data will be collected and entered from each site, whether to* ***complete the paper CRFs*** *or how to enter data electronically from each site.*
CRFs and outcome measures (questionnaires) will be completed electronically via the software REDCap, either by the RA (CRFs) or the participant (questionnaires). Where the participant will prefer paper questionnaires, these will be provided and returned via post, and the RA will enter the responses onto REDCap. If the participant requires face-to-face appointments, the RA will complete the CRFs and questionnaires either directly on REDCap or, if this is not possible, on paper and will then transfer the data onto REDCap.
Participants will also answer a few questions about their symptoms via the m-Path app.

4.2 *Provide details on the system used for data entry*
The REDCap online software will be used for collecting CRF and quantitative data. The m-Path app will be used for collecting information about participants' main symptoms (participant-completed).

4.3 *Outline who will be conducting the data entry from each site?*
The RA and the participants themselves.

4.4 *Outline whether single or double data entry will be carried out for all sites*
Single.

For peer review only - http://bmjopen.bmj.com/site/about/guidelines.xhtml

4.5 If double data entry is required describe the process and whether a 100% of CRFs or a sample of CRFs will be double entered. Outline how the two entries will be compared, who will carry out the comparison and how the results will be dealt with.
N/a

4.6 Outline the checks undertaken for outcome measures
The TM will perform regular self-monitoring visits in which the data entered for a proportion of participants will be checked for completeness, accuracy and timely completion. The dataset from the m-Path app will be downloaded and backed up weekly by the RA, and will be inspected by the TM during monitoring visits.

4.7 Provide details on how the data will be centralised
N/a – one site.

5. Data checks & data validation for each site

5.1 *Outline who will perform data cleaning, missing data checks, consistency checks, range checks and logic, and how these are checked at each site?*
The TM will perform regular self-monitoring visits in which the data entered for a proportion of participants will be checked for completeness, accuracy and timely completion. In addition, value range restrictions will be applied in REDCap and the m-Path app so that no values outside the available range can be entered when completing closed questions.

5.2 Describe how regularly the data will be checked
Monthly.

5.3 Provide contact person for each site for data queries if data managed centrally
N/a

5.4 *Describe the data flow from each site to central data centre, and who will conduct the overall data check*
N/a – one site.

*5.5 Detail the **flow of the data** from the field to the final storage for each site*
Data will be collected directly on study database (REDCap) or m-Path app. Any data from paper version of the CRFs and questionnaires will be entered on REDCap by the RA within 24 hours of collection.

6. AE and SAE data handling

Outline how the AE data will be collected by each site and collated for all sites at the end of the trial.
N/a – one site.

7. Partial and/or final data check and database lock

Detail if an interim analysis is planned in the trial protocol, stating the time point and whether the database will be partially locked for the interim analysis.
N/a

Detail the process for partial and final data checks and data lock, outline who checks the data and who will sign off for partial/final data lock form(s)?

For peer review only - http://bmjopen.bmj.com/site/about/guidelines.xhtml

The database will be locked after the following actions have been confirmed as completed by the TM:

1. All CRF data have been collected and entered onto REDCap.
2. All queries identified during regular data checks and self-monitoring visits have been resolved/clarified.
3. All missing information has been confirmed as being not available (as opposed to not entered).
4. All monitoring visits (incl. close-out) have been performed, and outstanding actions completed.
5. A final data quality check has been performed.

The TM will be responsible for locking the REDCap database (notifying the CI and Sponsor), send the REDCap and m-Path datasets to the trial statistician (with treatment allocation coded to prevent unblinding), and save a copy of the full databases in the TMF.

8. Data security and transmission between sites

Provide details on the data security procedures for transmitting data between sites
To comply with SWLSTG's Information Governance regulations, the datasets will be sent via an encrypted email to the trial statistician.

9. Data export & analysis

Explain how data will be exported and who the data should be sent to for data analysis.
The REDCap database with demographic and questionnaire data and data from m-Path app will be exported in CSV format and sent via encrypted email to Jared Smith, Trial Statistician and Co-investigator.

10. Data Back-up and archiving

Describe procedures in place to ensure data protection including back-up system (if you don't do this you could lose the data!)
The REDCap database and m-Path data will be downloaded and backed up weekly on a Trust external hard drive. The TMF will be saved on the Trust's shared drive, which is backed up every night, in a restricted folder dedicated to the study. In addition, the TMF will also be backed up once a week on the Trust encrypted external hard drive.
The study will be archived by the R&D team on the SWLSTG R&D shared drive.

For peer review only - http://bmjopen.bmj.com/site/about/guidelines.xhtml

NHS
South West London and
St George's Mental Health
NHS Trust

Participant Information Sheet

MODIFI: A feasibility study of eye MOvement DesensItisation and reprocessing therapy (EMDR) for FunctIonal neurological disorder (FND)

We would like to invite you to take part in this research. Before you decide whether you want to, it is important for you to read the following information carefully and discuss it with others if you wish. Please ask us if there is anything that is not clear or if you would like more information.

Brief summary

This research aims to evaluate the possibility of delivering, and potential benefit, of eye movement desensitisation and reprocessing therapy (EMDR) as a psychological therapy for functional neurological disorder (FND). This is a feasibility study which means that it will not have enough participants to demonstrate statistically whether EMDR is helpful or not, but it will test out the study procedures (such as recruitment and therapy attendance) and look at whether it seems to benefit participants or not. If the study shows that it is feasible and potentially beneficial, a larger trial will be designed. A larger trial will be able to find out whether receiving EMDR, in addition to routine neuropsychiatric care, is more helpful to patients than neuropsychiatric care alone. In order to receive funding for a larger trial, this feasibility study needs to be successful.

The feasibility study will recruit 50 participants with specific functional neurological symptoms (weakness, walking difficulties, jerks, shaking, and/or seizures) from the Neuropsychiatry Service at St. George's Hospital. Participants will be allocated randomly (i.e. by chance, using a computer programme) to either EMDR and routine neuropsychiatry appointments; or to routine neuropsychiatry appointments alone.

Background

Functional Neurological Disorder (FND) is a problem with the functioning of the nervous system and how the brain and body send and receive signals, rather than due to neurological disease or injury. FND causes a range of neurological symptoms, such as seizures, shaking, weakness, and paralysis. The symptoms are associated with significant distress and disability. Treatment for FND in the United Kingdom is limited, and the evidence-base for treatment is poor, despite it being a common condition.

A psychological therapy called cognitive-behavioural therapy (also commonly known as CBT) has been found to be beneficial, but it does not help everyone. EMDR is a psychological therapy that has combined ideas from several therapies, including cognitive-behavioural, psychodynamic, and experiential. The basic idea behind EMDR is that psychological distress originates from upsetting memories in a person's past, and that targeting those key memories will result in a reduction of distress. EMDR is a well-established treatment for PTSD, but there is increasing evidence that is also helpful for many other difficulties, including depression, anxiety disorders, tinnitus, and chronic pain. There is a small amount of case study evidence that EMDR can be useful for treating FND, but proper scientific testing is needed.

EMDR should only be delivered by suitably qualified psychologists or psychological therapists, who have undergone the EMDR training required and who receive EMDR supervision. Within EMDR, key memories or images are identified that could be targeted in therapy. Each EMDR processing session follows a standardised protocol. As part of this protocol, a patient is asked to bring the target image to mind, with an identified negative cognition, notice where they are feeling the distress in their body, and follow the clinician's fingers with their eyes. After each set of eye movements, they are asked what they noticed, and importantly, without discussion, they are told to "go with that" alongside the eye movements. It may involve multiple sessions for the distress to reduce sufficiently. It should be

For peer review only - http://bmjopen.bmj.com/site/about/guidelines.xhtml

noted that alternative "bilateral stimulation" tasks, instead of eye movements, can be used, such as alternate tapping or audio-tones. There are several theories regarding why the eye movements, or dual-attention on another task, may facilitate emotional processing, but like other psychological therapies, the specific mechanisms of how it works are not clear. It may be that the eye movements (or other task) allow the person to stay in an optimal zone for emotional processing (i.e. not too distressed). Another theory is that engaging in two simultaneous tasks taxes working memory, which has limited capacity. Therefore "bilateral stimulation" whilst focusing on the target memory/image results in less space for the distressing memory/image, and it is stored back in memory less vividly with reduced disturbance.

Why have I been chosen?

We are recruiting people diagnosed with FND by a neurologist, who have been referred to the Neuropsychiatry Service. During the appointment you attended with a neuropsychiatrist, they identified that you experienced specific functional neurological symptoms, such as weakness, walking difficulties, jerks, shaking, and/or seizures, and that you were likely suitable to take part in this study. They asked you whether you would be willing to be contacted about the study, and following your consent, passed your contact details on to the research team.

What will I have to do?

If you are interested in taking part in the study, please let the research team know by emailing modifi.recruitment@swlstg.nhs.uk uk or telephoning +44 20 3513 5191. The research assistant will contact you to arrange a convenient time to attend a screening assessment in order to check you are able to participate. The screening assessment can be carried out in-person at St. George's or Springfield Hospital or remotely via MS Teams, whichever is best for you.

If you choose to attend the screening assessment, it will last between 30 minutes and 2 hours. During the appointment, you will be able to ask any questions. You will be asked to sign a consent form indicating that you agree to take part in the study. Agreement to take part will include you agreeing to therapy sessions to be video recorded if you are allocated to the group of participants attending EMDR alongside your routine neuropsychiatry appointments (please see below section on data protection and confidentiality for details).

You will then be asked to complete a questionnaire that asks you whether you have experienced any specific potentially traumatic life events in your lifetime, such as being assaulted, experiencing a life-threatening illness, being in an accident or being bullied. To take part in this study, you will need to have experienced at least 1 potentially traumatic event in your lifetime. You will not be asked to discuss the event(s) in the screening assessment, only indicate whether or not any have happened to you. Following this you will be asked to complete another questionnaire that examines any instances of dissociation you may have had over the previous 4 weeks. Dissociation is a broad term that refers to experiences of disconnection or lack of integration in your experience, such as your body feeling like it was someone else's, things around you suddenly seeming strange, or feeling like two or more people were fighting or arguing inside of yourself. There is a particular type of dissociation called identity disturbance and we will test for this as part of the assessment, as we will not be inviting anyone with this condition to take part in the research.

If the screening assessment tells us that you are eligible to take part in the study, we will ask you to complete additional questionnaires.

If you are not eligible to take part in the study and are interested in attending psychological therapy, you will be offered an appointment with Dr Cope, Principal Clinical Psychologist in the Neuropsychiatry Service, who will be able to discuss your current difficulties and psychological therapy options with you.

For peer review only - http://bmjopen.bmj.com/site/about/guidelines.xhtml

What would taking part involve?

Completing questionnaires

Once it is confirmed you are eligible to take part in the study, you will be asked to complete additional questionnaires regarding your health-related functioning, mental health, and use of healthcare services. You will also be asked to complete questions on your demographics (e.g. relationship status, living arrangements, employment status), your medical history, current medication, and any previous psychological therapy you have attended, e.g. type, number of sessions, and focus of intervention (if known).

You will also be asked to identify the FND symptoms, e.g. seizures, tremor, limb weakness, tingling/numbness, gait disturbance (maximum two symptoms) that have the worst impact on your quality of life. You will then be given instructions regarding rating each symptom daily for 2 weeks. This can be done via an App or on paper if you prefer.

These are called "baseline measures", and you will be asked to complete questionnaires at 3 more time points: 3 months, 6 months and 9 months after your screening appointment. The research assistant will contact you at these time points to arrange completion of the questionnaires.

Randomisation (Allocation to treatment group)

Following your consent and completion of the baseline measures, you will be allocated to either EMDR plus routine appointments with your allocated neuropsychiatrist (EMDR+NPC), or routine neuropsychiatric appointments alone (NPC). You will be allocated at random by a computer programme: the research team will not be able to predict or change which group you will be assigned to. You will be told which group you have been randomly allocated to by the Trial Manager. The research assistant will not be told which group you have been allocated to, and it is important that you do not let them know which group you are in, at any point in the study. This is because if they know which group you are allocated to, it may influence how they support you in the completion of the questionnaires completed at different time points in the research.

Treatment groups

You will be invited to routine appointments with your assigned neuropsychiatrist (NPC). It is likely you will be invited to 1-3 routine neuropsychiatric appointments during the research. Each of these routine medical appointments lasts for 30 minutes. Your neuropsychiatrist may refer you to educational FND groups run in the service or for treatments for any other difficulties you may have.

If you are allocated to EMDR+NPC, you will be contacted by a psychological therapist to arrange your first EMDR appointment shortly after completing your screening. Your EMDR appointments can be held in-person at St. George's Hospital or virtually via MS Teams (your choice). You will be offered up to 16 sessions of EMDR, with each session lasting between 60 and 90 minutes (treatment duration will depend on your individual needs). The sessions will need to be completed within 6 months, so it is important that you are available and willing to meet with your therapist weekly. A minimum number of 8 attended EMDR sessions will count as "completed treatment". An optional follow-up session will be offered 1 month after treatment completion. Each of your sessions will be video recorded. This is to check the therapist is following the treatment protocol and for the therapist to review sessions in their clinical supervision.

Whether you are allocated to EMDR+NPC or NPC alone, we ask that during the study period (9 months), you do not start attending any other individual psychological therapies focused on FND or specific FND treatments, such as inpatient treatment or specialist intensive physiotherapy for FND. Your neuropsychiatrist or other doctors involved in your care can still refer you to these treatments during the trial period (if needed); we just ask you not to start any of them during the time you are part of the study if possible. If you do start attending individual psychological therapy for FND which is not part of the study, or the specific FND treatments mentioned, you will not be able to continue to take part in the study, and any data collected up until that point will be used for analysis. This is because

For peer review only - http://bmjopen.bmj.com/site/about/guidelines.xhtml

South West London and St George's Mental Health NHS Trust

we would like to compare EMDR+NPC to NPC alone, and if you receive another specific FND treatment in the same time period, we will not be able to know whether any changes are due to the treatment we are offering or the treatments you are receiving elsewhere.

Travel expenses and payment for participation

You will be reimbursed for research-related travel costs, up to the value of £20 per appointment (if you choose to attend appointments in-person, rather than virtually). Receipts/proof of travel will need to be provided.

If you are enrolled onto the trial, you will be offered a £25 incentive 9-months after consenting to participate, irrespective of whether you complete the trial or not.

Do I have to take part?

No. It is up to you to decide whether to take part. If you choose not to take part, it will not affect any routine care that you receive.

How long does the study last?

If you take part, your participation in the trial will end after 9 months, once the final assessment measures have been completed. If you take part, you will also be asked whether you would be willing to be contacted after completion of the study to potentially provide additional data for a prospective full-scale study, and/or be invited to participate in future studies.

When all participants have completed the study, some people will be invited to attend interviews regarding their experiences of the study. If you are invited to attend an interview, this will be entirely voluntary, and you will be given further information beforehand so that you can decide whether you would like to be interviewed or not.

Following your participation in the trial, your clinical care will continue to be provided by the neuropsychiatry service, if needed, and will be assessed on your individual basis.

What are the possible benefits of taking part?

We cannot guarantee any specific treatment benefits, but it is hoped that those allocated to EMDR + NPC will benefit from the therapy. Taking part in this research will contribute to the evidence-base regarding treatments for FND. Contributing to research on FND treatments has the potential to help many people with FND, as the results of this study will likely inform treatment provision for FND.

What are the possible disadvantages and risks of taking part?

Attending mental health treatments like neuropsychiatric appointments and/or psychological therapy like EMDR may result in an increase in distress and/or deterioration in physical or mental health. However, these potential changes are usually temporary. They may occur as a result of discussing difficult events, emotions and symptoms in the appointments. If you are randomly selected for EMDR+NPC, it is possible that during your EMDR sessions, focusing on a distressing memory may cause you to experience an increase in the physical sensations or emotions associated with that memory. However, this is likely to be temporary and it is hoped that any potential discomfort will be outweighed by the potential benefits of EMDR. Irrespective of which treatment group you are allocated to, if you experience an increase in distress and/or deterioration in your physical or mental health, you will be supported by the neuropsychiatry healthcare professional(s) involved in your care, and if needed, signposted or referred to relevant organisations or support resources.

For peer review only - http://bmjopen.bmj.com/site/about/guidelines.xhtml

South West London and St George's Mental Health NHS Trust

What if I don't want to carry on with the research project?

It is up to you to decide whether to take part or not; choosing not to take part will not disadvantage you in any way or affect any treatment you are receiving outside of the study. You are free to withdraw at any time without giving a reason. If you withdraw, it will not affect any routine care that you receive. If you withdraw early from the study, any data you provided up until that point will be used for analysis.

What if something goes wrong?

If you have a concern about any aspect of this study, you should contact the trial manager Dr Serena Vanzan or chief investigator Dr Sarah Cope [contact number 020 8725 3786, email modifi@swlstg.nhs.uk; sarah.cope@swlstg.nhs.uk]. If you are not satisfied following this, you can contact the Sponsor's Office by emailing ResearchDevelopment@swlstg.nhs.uk. If you remain unhappy and wish to complain formally, you can do this by contacting complaints@swlstg.nhs.uk. Further details can be obtained from https://www.swlstg.nhs.uk/contact-us/complaints. Or you can contact the Trust's Patient Advice and Liaison Service (PALS). Tel: 0203 513 6150 (Monday - Friday 9.30am to 4.30pm) or email pals@swlstg.nhs.uk.

In the event that something does go wrong and you are harmed during the research and this is due to someone's negligence then you may have grounds for a legal action for compensation against SWLSTG NHS Trust, but you may have to pay your legal costs. The normal National Health Service complaints mechanisms will still be available to you.

Will my taking part in this study be kept confidential?

All the information that we collect about you during the course of the research will be kept strictly confidential. The only limits to this confidentiality are that if you share information that suggests you or someone else is at risk of harm, or if you disclose a serious crime has been committed. If this happens, the researcher or clinician will need to share this information with those directly involved in your care. The researcher or clinician would try to discuss this with you before confidentiality is broken.

You will not be able to be identified in any ensuing reports or publications. All data will be pseudonymised. This means that you cannot be personally identified. Each participant will be assigned a unique screening number upon referral to the study. This number will be written on all eligibility measures and databases. A unique trial identification number will then be issued following your consent to take part. This number will be written on all clinical assessment forms/datasheets and databases used to record data on participants. All data will be kept secure at all times and maintained in accordance with General Data Protection Regulation (GDPR, 2018) requirements and archived according to clinical trial Good Clinical Practice regulations. Only researchers working with the Chief Investigator (Dr. Cope) will have access to the data. The data custodian for this project will be Dr. Cope. The data will be retained for 10 years after completion of the study.

How will we use information about you?

We will need to use information from your medical records for this research project. This information will include your:

- NHS number
- Hospital number
- Name
- Contact details
- Date of birth
- Ethnicity
- Gender
- Relationship status
- Employment status
- Presence of dependent(s)
- Presence of a carer

For peer review only - http://bmjopen.bmj.com/site/about/guidelines.xhtml

South West London and St George's Mental Health NHS Trust

- Receipt of state benefits
- Current medication
- Medical history

People will use this information to do the research or to check your records to make sure that the research is being done properly. People who do not need to know who you are will not be able to see your name or contact details. Your data will have a code number instead. We will keep all information about you safe and secure.

Once we have finished the study, we will keep some of the data so we can check the results. We will write our reports in a way that no-one can work out that you took part in the study.

What are your choices about how your information is used?

You can stop being part of the study at any time, without giving a reason, but we will keep information about you that we already have.

- We need to manage your records in specific ways for the research to be reliable. This means that we won't be able to let you see or change the data we hold about you.
- If you agree to take part in this study, you will have the option to take part in future research using your data saved from this study. If you consent to this we may:
  - Use your data already collected for this study in future research.
  - Contact you regarding taking part in future research relating to this current study.

Where can you find out more about how your information is used?

You can find out more about how we use your information

- at www.hra.nhs.uk/information-about-patients/
- by contacting the trial team at modifi@swlstg.nhs.uk
- by sending an email to SWLSTG's Data Protection Officer, John Hughes, at john.hughes@swlstg.nhs.uk
- by ringing us on +44 20 3513 5191

Informing General Practitioner (GP)

If you choose to participate, your GP will be informed that you are participating in the study.

What will happen to the results of the study?

We will disseminate the results from this study in conferences, peer-reviewed journals, social media, and the website for the patient-led charity FND Hope.

We will email you a copy of the paper that presents the findings from this study once the paper has been published.

Who is organising and funding the research?

SWLSTG is the study sponsor. This project is funded by the National Institute for Health and Care Research (NIHR) under its Research for Patient Benefit (RfPB) Programme (Grant Reference Number NIHR202277).

For peer review only

For peer review only - http://bmjopen.bmj.com/site/about/guidelines.xhtml

How have patients and the public been involved in this study?

The research design has been informed by a Public and Patient Involvement (PPI) meeting, where all participants had lived experience of FND. The research team has people with lived experience of FND. Two people are on the Trial Steering Committee and three other people are working with other members of the research team to make sure that the lived experience perspective is reflected in the research tools, methodology, analysis and dissemination. If the trial proves feasible, they will contribute to the design of a substantive study. The study has received guidance and support on PPI and lived experience involvement from the lived experience PPI lead for Research & Development at SWLSTG.

Who has reviewed the study?

All proposals for research using human subjects are reviewed by an Ethics Committee before they can proceed. This proposal was reviewed by West Midlands - Edgbaston Research Ethics Committee. This study has also been reviewed by the Health Research Authority.

Further information and contact details

Please contact modifi@swlstg.nhs.uk or telephone +44 20 3513 5191 if you require further information about this study.

The Chief Investigator is Dr Sarah Cope (Principal Clinical Psychologist) and her contact details are:

Neuropsychiatry Service

2nd Floor Grosvenor Wing,

St. George's Hospital,

Blackshaw Road,

SW17 0QT

(T) 020 8725 3786

(E) sarah.cope@swlstg.nhs.uk

Thank you for reading this information sheet and for your consideration regarding taking part in this research study.

For peer review only

For peer review only - http://bmjopen.bmj.com/site/about/guidelines.xhtml

South West London and St George's Mental Health NHS Trust

MODIFI: Randomised feasibility study of eye MOvement DesensItisation and reprocessing therapy (EMDR) for FunctIonal neurological disorder (FND)

Informed Consent Form

(Version 1.3, 23Nov2022)

Participant Number:_______________________

Chief Investigator: Dr Sarah Cope

Please initial box

1. I confirm that I have read the information sheet dated................... (version............) for the above study. I have had the opportunity to consider the information, ask questions and have had these answered satisfactorily. ☐

2. I understand that my participation is voluntary and that I am free to withdraw at any time without giving any reason, without my medical care or legal rights being affected. ☐

3. I understand that relevant sections of my medical notes and data collected during the study, may be looked at by individuals from regulatory authorities or from SWLSTG NHS Trust, where it is relevant to my taking part in this research. I give permission for these individuals to have access to my records. ☐

4. I understand my personal data (such as name and contact details) will be kept for a maximum of 5 years following the end of the study and anonymised data (gathered during the trial) could be securely shared with appropriate research teams for further analysis (only with permission from SWLSTG NHS Trust). ☐

5. I understand that the information collected about me will be used to support other research in the future and may be shared anonymously with other researchers. ☐

6. I agree to my General Practitioner being informed of my participation in the study. ☐

For peer review only - http://bmjopen.bmj.com/site/about/guidelines.xhtml

For peer review only

South West London and St George's Mental Health NHS Trust

7. I understand that the information held and maintained by South West London & St. George's Mental Health NHS Trust may be used to help contact me or provide information about my health status. ☐

8. I understand that if I am allocated to EMDR plus neuropsychiatric care, my sessions of EMDR will be video-recorded for the purposes of checking the therapist is following the treatment protocol. ☐

9. I agree to be contacted for future research. ☐

10. I agree to take part in the above study. ☐

Name of Participant	Date	Signature
Name of Person taking consent	Date	Signature

Please make sure you have initialled the boxes if you agree.

When completed - 1 copy for patient, 1 original copy for Trial Master File and 1 copy for hospital records

For peer review only - http://bmjopen.bmj.com/site/about/guidelines.xhtml

Standard Protocol Items: Recommendations for Interventional Trials

SPIRIT 2013 Checklist: Recommended items to address in a clinical trial protocol and related documents*

Section/item	ItemNo	Description	Page Number
**Administrative information**			
Title	1	Descriptive title identifying the study design, population, interventions, and, if applicable, trial acronym	i
Trial registration	2a	Trial identifier and registry name. If not yet registered, name of intended registry	ii
	2b	All items from the World Health Organization Trial Registration Data Set	
Protocol version	3	Date and version identifier	i
Funding	4	Sources and types of financial, material, and other support	i
Roles and responsibilities	5a	Names, affiliations, and roles of protocol contributors	i, 14
	5b	Name and contact information for the trial sponsor	14
	5c	Role of study sponsor and funders, if any, in study design; collection, management, analysis, and interpretation of data; writing of the report; and the decision to submit the report for publication, including whether they will have ultimate authority over any of these activities	14
	5d	Composition, roles, and responsibilities of the coordinating centre, steering committee, endpoint adjudication committee, data management team, and other individuals or groups overseeing the trial, if applicable (see Item 21a for data monitoring committee)	14, Supplementary Material (SM) p. 12-13
**Introduction**			
Background and rationale	6a	Description of research question and justification for undertaking the trial, including summary of relevant studies (published and unpublished) examining benefits and harms for each intervention	1-3
	6b	Explanation for choice of comparators	
Objectives	7	Specific objectives or hypotheses	3

For peer review only

Trial design	8	Description of trial design including type of trial (eg, parallel group, crossover, factorial, single group), allocation ratio, and framework (eg, superiority, equivalence, noninferiority, exploratory)	3

Methods: Participants, interventions, and outcomes

Study setting	9	Description of study settings (eg, community clinic, academic hospital) and list of countries where data will be collected. Reference to where list of study sites can be obtained	3
Eligibility criteria	10	Inclusion and exclusion criteria for participants. If applicable, eligibility criteria for study centres and individuals who will perform the interventions (eg, surgeons, psychotherapists)	4
Interventions	11a	Interventions for each group with sufficient detail to allow replication, including how and when they will be administered	4-6
	11b	Criteria for discontinuing or modifying allocated interventions for a given trial participant (eg, drug dose change in response to harms, participant request, or improving/worsening disease)	n/a
	11c	Strategies to improve adherence to intervention protocols, and any procedures for monitoring adherence (eg, drug tablet return, laboratory tests)	9
	11d	Relevant concomitant care and interventions that are permitted or prohibited during the trial	6
Outcomes	12	Primary, secondary, and other outcomes, including the specific measurement variable (eg, systolic blood pressure), analysis metric (eg, change from baseline, final value, time to event), method of aggregation (eg, median, proportion), and time point for each outcome. Explanation of the clinical relevance of chosen efficacy and harm outcomes is strongly recommended	6-7
Participant timeline	13	Time schedule of enrolment, interventions (including any run-ins and washouts), assessments, and visits for participants. A schematic diagram is highly recommended (see Figure)	Fig. 1
Sample size	14	Estimated number of participants needed to achieve study objectives and how it was determined, including clinical and statistical assumptions supporting any sample size calculations	10
Recruitment	15	Strategies for achieving adequate participant enrolment to reach target sample size	9

Methods: Assignment of interventions (for controlled trials)

Allocation:

For peer review only - http://bmjopen.bmj.com/site/about/guidelines.xhtml

Sequence generation	16a	Method of generating the allocation sequence (eg, computer-generated random numbers), and list of any factors for stratification. To reduce predictability of a random sequence, details of any planned restriction (eg, blocking) should be provided in a separate document that is unavailable to those who enrol participants or assign interventions	10
Allocation concealment mechanism	16b	Mechanism of implementing the allocation sequence (eg, central telephone; sequentially numbered, opaque, sealed envelopes), describing any steps to conceal the sequence until interventions are assigned	10
Implementation	16c	Who will generate the allocation sequence, who will enrol participants, and who will assign participants to interventions	10
Blinding (masking)	17a	Who will be blinded after assignment to interventions (eg, trial participants, care providers, outcome assessors, data analysts), and how	10
	17b	If blinded, circumstances under which unblinding is permissible, and procedure for revealing a participant's allocated intervention during the trial	
**Methods: Data collection, management, and analysis**			
Data collection methods	18a	Plans for assessment and collection of outcome, baseline, and other trial data, including any related processes to promote data quality (eg, duplicate measurements, training of assessors) and a description of study instruments (eg, questionnaires, laboratory tests) along with their reliability and validity, if known. Reference to where data collection forms can be found, if not in the protocol	6-9
	18b	Plans to promote participant retention and complete follow-up, including list of any outcome data to be collected for participants who discontinue or deviate from intervention protocols	9
Data management	19	Plans for data entry, coding, security, and storage, including any related processes to promote data quality (eg, double data entry; range checks for data values). Reference to where details of data management procedures can be found, if not in the protocol	12, SM p. 15
Statistical methods	20a	Statistical methods for analysing primary and secondary outcomes. Reference to where other details of the statistical analysis plan can be found, if not in the protocol	10-11, SM p.8
	20b	Methods for any additional analyses (eg, subgroup and adjusted analyses)	
	20c	Definition of analysis population relating to protocol non-adherence (eg, as randomised analysis), and any statistical methods to handle missing data (eg, multiple imputation)	SM p. 8

Methods: Monitoring

Data monitoring	21a	Composition of data monitoring committee (DMC); summary of its role and reporting structure; statement of whether it is independent from the sponsor and competing interests; and reference to where further details about its charter can be found, if not in the protocol. Alternatively, an explanation of why a DMC is not needed	12, SM p. 12-13
	21b	Description of any interim analyses and stopping guidelines, including who will have access to these interim results and make the final decision to terminate the trial	n/a
Harms	22	Plans for collecting, assessing, reporting, and managing solicited and spontaneously reported adverse events and other unintended effects of trial interventions or trial conduct	SM p. 10-11
Auditing	23	Frequency and procedures for auditing trial conduct, if any, and whether the process will be independent from investigators and the sponsor	12

Ethics and dissemination

Research ethics approval	24	Plans for seeking research ethics committee/institutional review board (REC/IRB) approval	12
Protocol amendments	25	Plans for communicating important protocol modifications (eg, changes to eligibility criteria, outcomes, analyses) to relevant parties (eg, investigators, REC/IRBs, trial participants, trial registries, journals, regulators)	SM p. 14
Consent or assent	26a	Who will obtain informed consent or assent from potential trial participants or authorised surrogates, and how (see Item 32)	9, 12
	26b	Additional consent provisions for collection and use of participant data and biological specimens in ancillary studies, if applicable	n/a
Confidentiality	27	How personal information about potential and enrolled participants will be collected, shared, and maintained in order to protect confidentiality before, during, and after the trial	12
Declaration of interests	28	Financial and other competing interests for principal investigators for the overall trial and each study site	14
Access to data	29	Statement of who will have access to the final trial dataset, and disclosure of contractual agreements that limit such access for investigators	12
Ancillary and post-trial care	30	Provisions, if any, for ancillary and post-trial care, and for compensation to those who suffer harm from trial participation	12

For peer review only - http://bmjopen.bmj.com/site/about/guidelines.xhtml

Dissemination policy	31a	Plans for investigators and sponsor to communicate trial results to participants, healthcare professionals, the public, and other relevant groups (eg, via publication, reporting in results databases, or other data sharing arrangements), including any publication restrictions	12
	31b	Authorship eligibility guidelines and any intended use of professional writers	12
	31c	Plans, if any, for granting public access to the full protocol, participant-level dataset, and statistical code	n/a
**Appendices**			
Informed consent materials	32	Model consent form and other related documentation given to participants and authorised surrogates	SM files
Biological specimens	33	Plans for collection, laboratory evaluation, and storage of biological specimens for genetic or molecular analysis in the current trial and for future use in ancillary studies, if applicable	n/a

*It is strongly recommended that this checklist be read in conjunction with the SPIRIT 2013 Explanation & Elaboration for important clarification on the items. Amendments to the protocol should be tracked and dated. The SPIRIT checklist is copyrighted by the SPIRIT Group under the Creative Commons "Attribution-NonCommercial-NoDerivs 3.0 Unported" license.

For peer review only
