# Supplementary material for: MODIFI: protocol for randomised feasibility study of eye-movement desensitisation and reprocessing therapy (EMDR) for functional neurological disorder (FND)
Source: BMJ Open. 2023 Jun 2;13(6):e073727. doi: 10.1136/bmjopen-2023-073727 (PMC10255051; doi:10.1136/bmjopen-2023-073727)
Supplement: Reviewer comments [file bmjopen-2023-073727.reviewer_comments.pdf]

PEER REVIEW HISTORY

BMJ Open publishes all reviews undertaken for accepted manuscripts. Reviewers are asked to complete a checklist review form (**http://bmjopen.bmj.com/site/about/resources/checklist.pdf**) and are provided with free text boxes to elaborate on their assessment. These free text comments are reproduced below.

ARTICLE DETAILS

**TITLE (PROVISIONAL)**	MODIFI: Protocol for randomised feasibility study of eye movement desensitisation and reprocessing therapy (EMDR) for functional neurological disorder (FND)
**AUTHORS**	Cope, Sarah; Smith, Jared; El-Leithy, Sharif; Vanzan, Serena; Pentland, Caitlin; Pick, Susannah; Golder, Dawn; Hogwood, Patricia; Turner, Kati; Billings, Jo; Edwards, Mark

This article was not externally reviewed at BMJ Open. Protocols that have been independently assessed prior to submission to BMJ Open are usually fast-tracked to publication on the grounds that further substantial changes will not be possible. This independent assessment will usually be external, independent review for both a substantial grant award from a non-commercial or government funder and ethics approval.
